# Supplementary figures and images for: Scabertopin Derived from Elephantopus scaber L. Mediates Necroptosis by Inducing Reactive Oxygen Species Production in Bladder Cancer In Vitro
Source: Cancers (Basel). 2022 Dec 2;14(23):5976. doi: 10.3390/cancers14235976 (PMC9738305; doi:10.3390/cancers14235976)

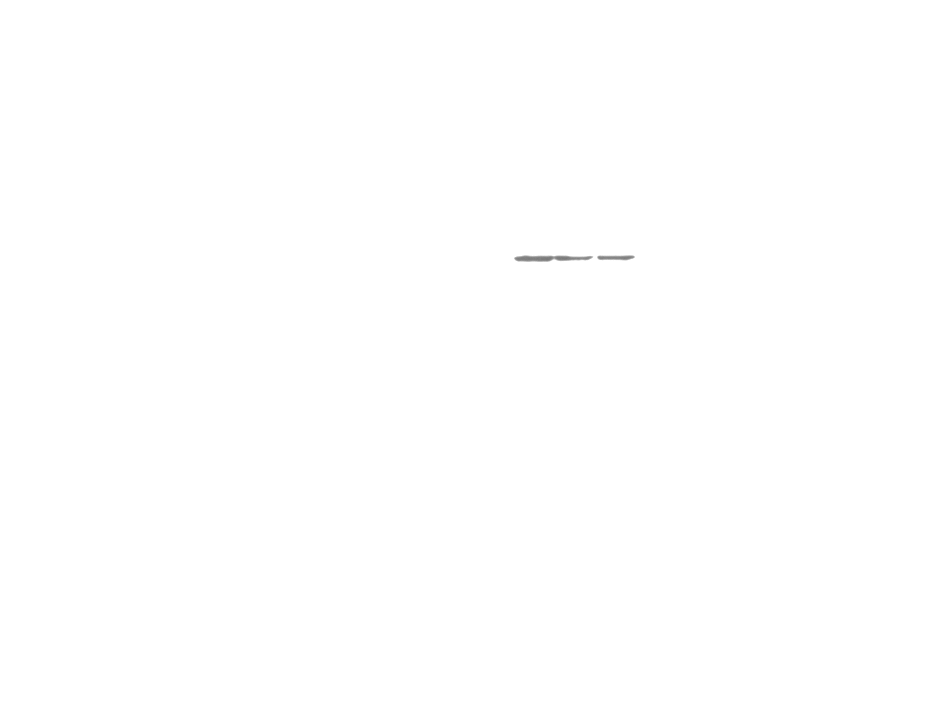

Supplement: Supplementary file 1 [file cancers-14-05976-s001.zip › Original images for blots or gels/Fig3-F/Bax-1.tif]

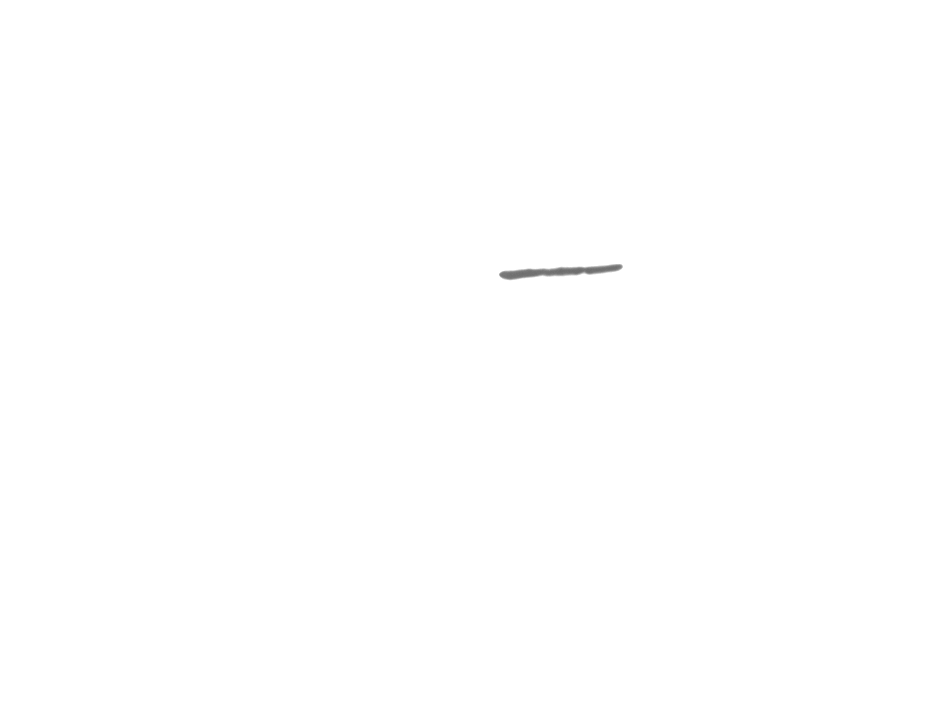

Supplement: Supplementary file 1 [file cancers-14-05976-s001.zip › Original images for blots or gels/Fig3-F/Bax-2.tif]

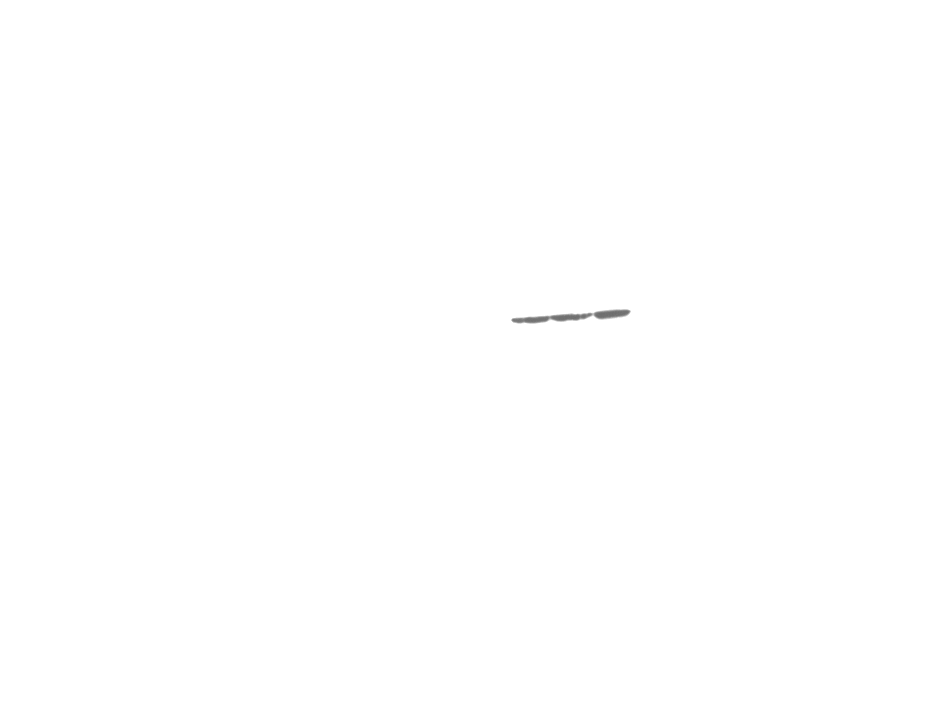

Supplement: Supplementary file 1 [file cancers-14-05976-s001.zip › Original images for blots or gels/Fig3-F/Bax-3.tif]

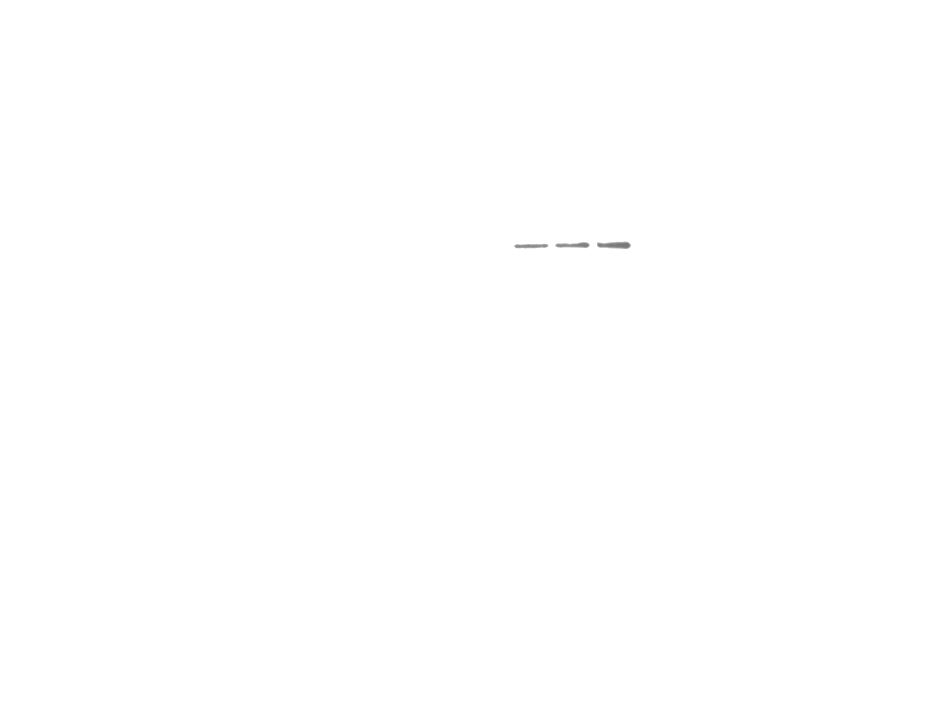

Supplement: Supplementary file 1 [file cancers-14-05976-s001.zip › Original images for blots or gels/Fig3-F/Bcl-1.tif]

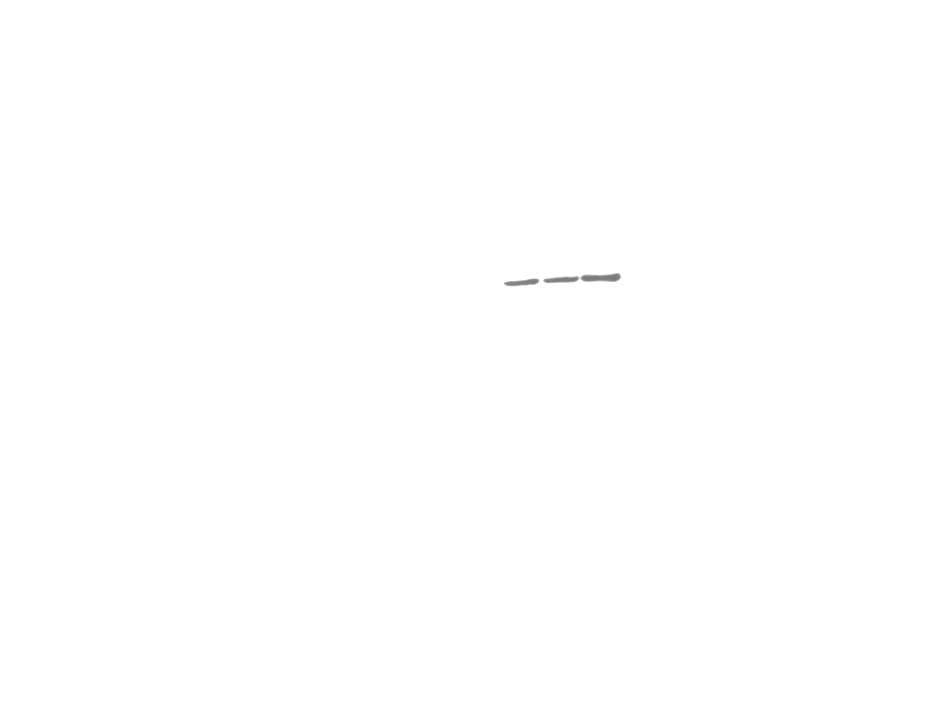

Supplement: Supplementary file 1 [file cancers-14-05976-s001.zip › Original images for blots or gels/Fig3-F/Bcl-2.tif]

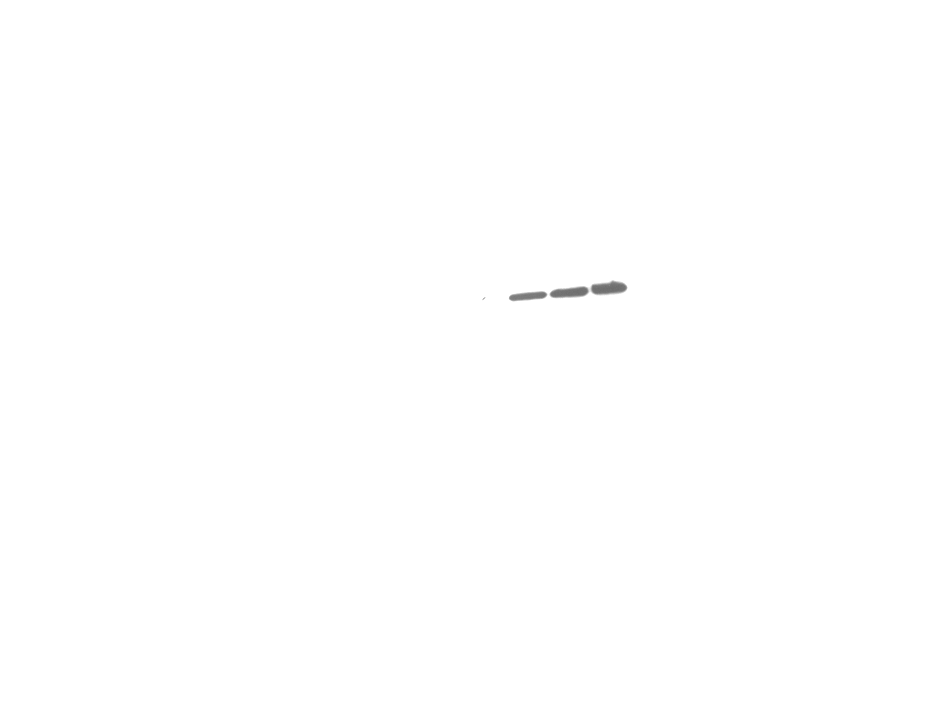

Supplement: Supplementary file 1 [file cancers-14-05976-s001.zip › Original images for blots or gels/Fig3-F/Bcl-3.tif]

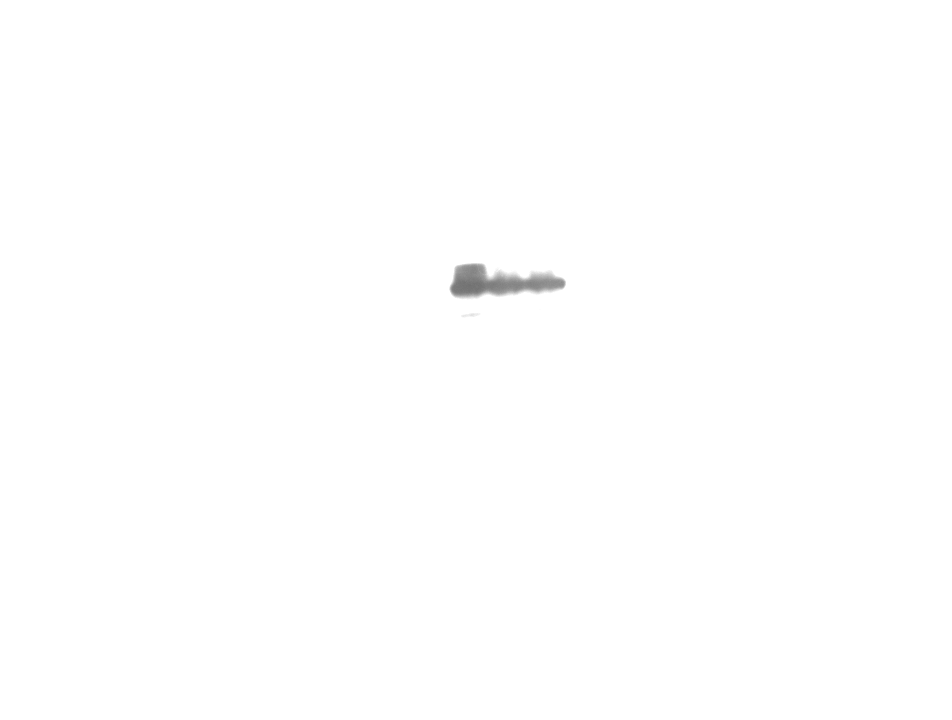

Supplement: Supplementary file 1 [file cancers-14-05976-s001.zip › Original images for blots or gels/Fig3-F/Caspase3-1.tif]

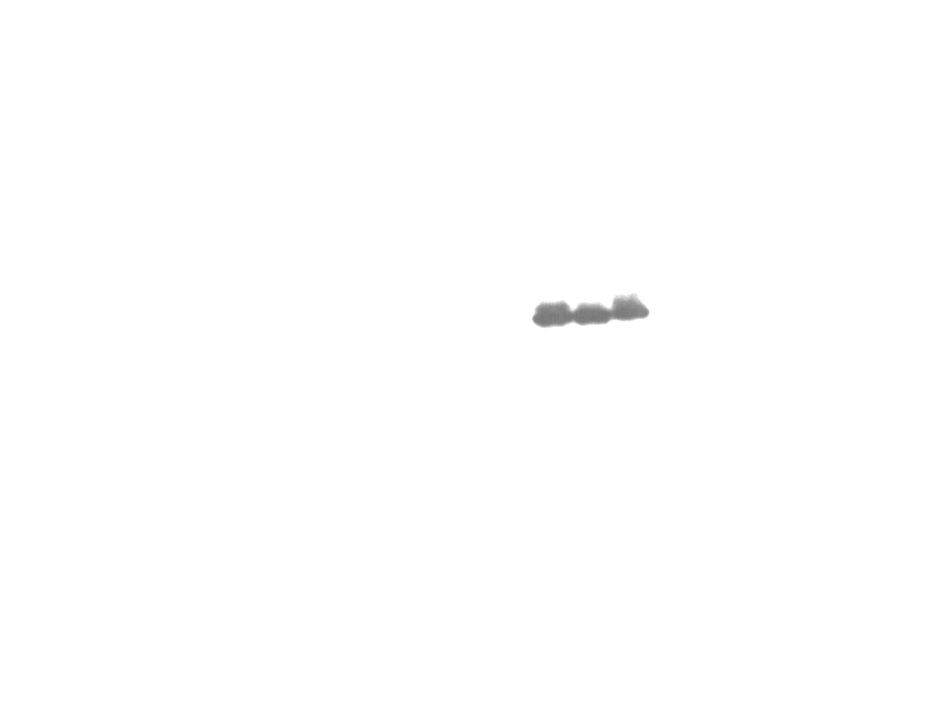

Supplement: Supplementary file 1 [file cancers-14-05976-s001.zip › Original images for blots or gels/Fig3-F/Caspase3-2.tif]

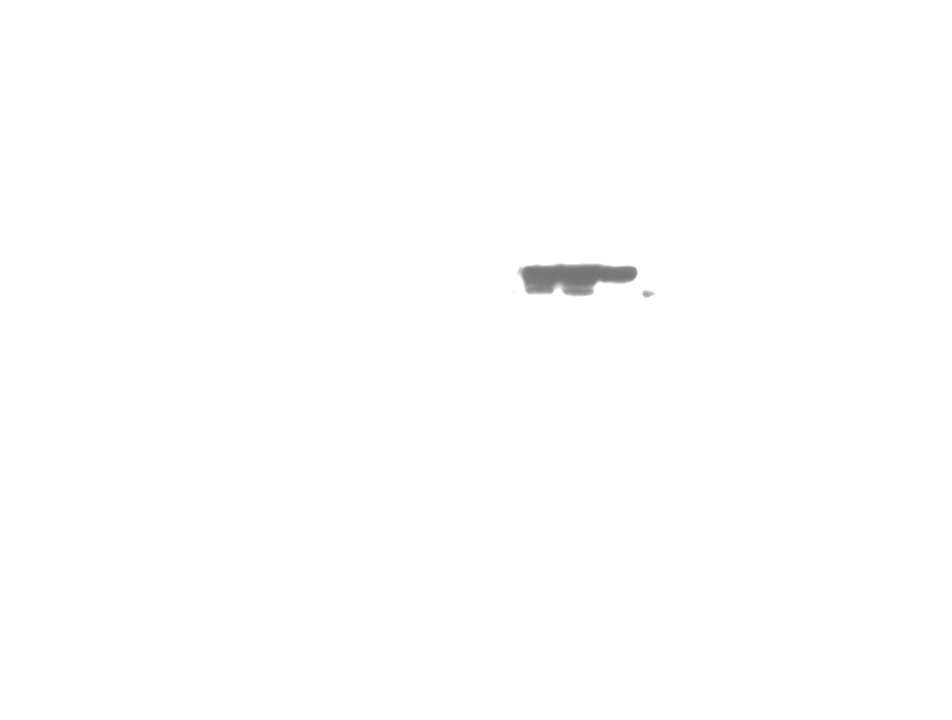

Supplement: Supplementary file 1 [file cancers-14-05976-s001.zip › Original images for blots or gels/Fig3-F/Caspase3-3.tif]

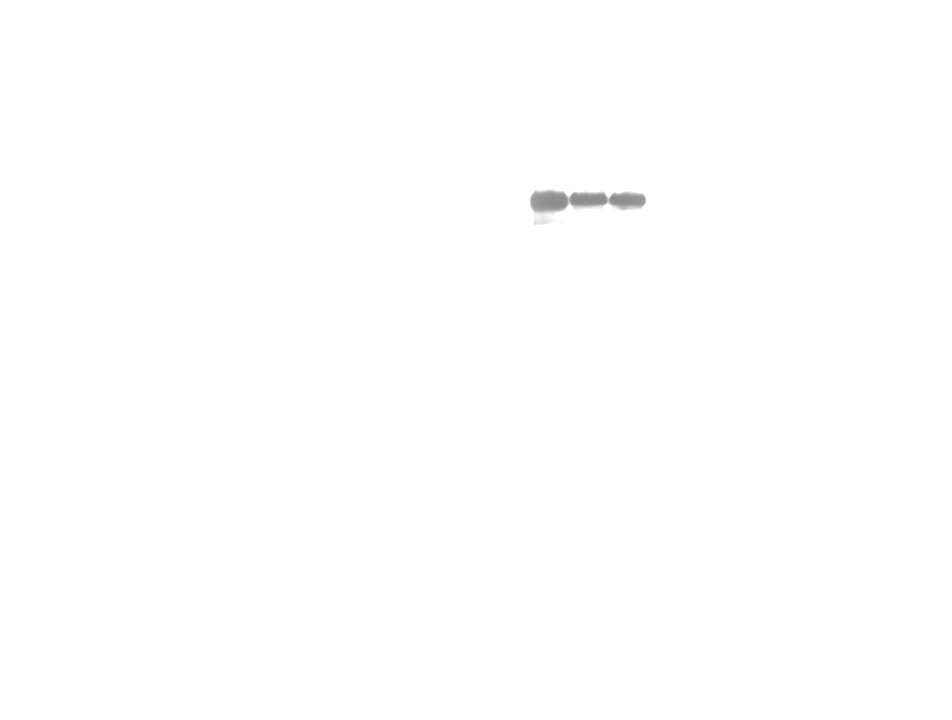

Supplement: Supplementary file 1 [file cancers-14-05976-s001.zip › Original images for blots or gels/Fig3-F/Caspase8-1.tif]

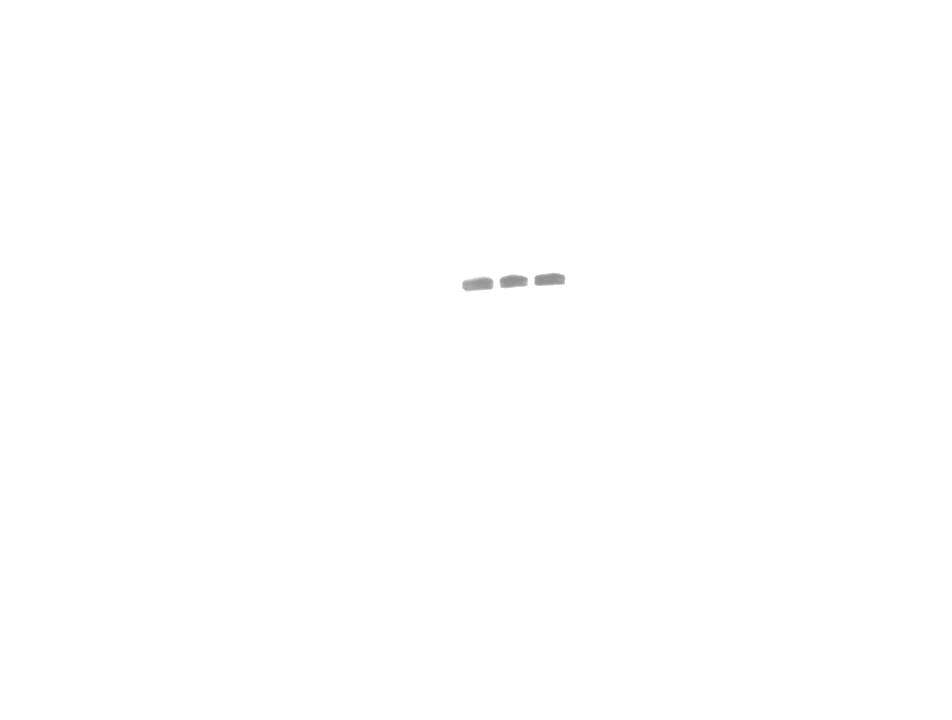

Supplement: Supplementary file 1 [file cancers-14-05976-s001.zip › Original images for blots or gels/Fig3-F/Caspase8-2.tif]

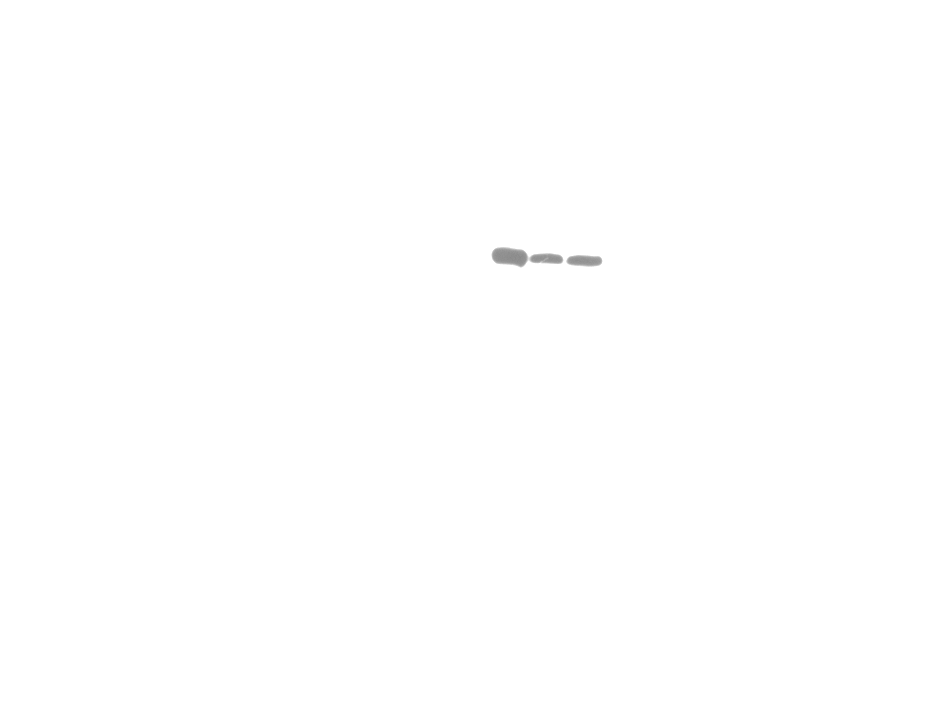

Supplement: Supplementary file 1 [file cancers-14-05976-s001.zip › Original images for blots or gels/Fig3-F/Caspase8-3.tif]

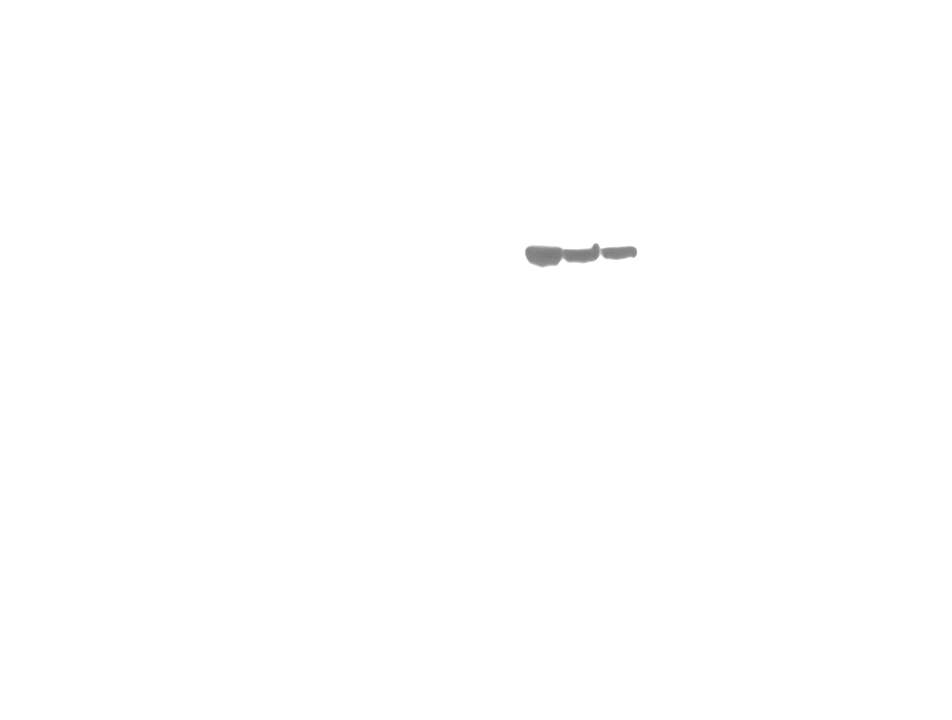

Supplement: Supplementary file 1 [file cancers-14-05976-s001.zip › Original images for blots or gels/Fig3-F/Caspase9-1.tif]

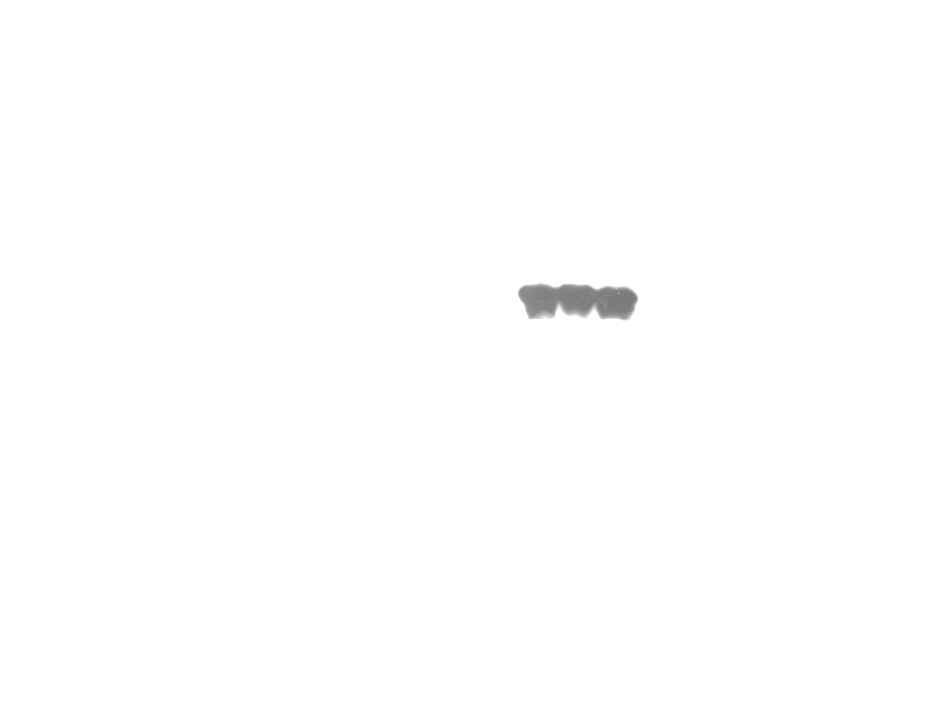

Supplement: Supplementary file 1 [file cancers-14-05976-s001.zip › Original images for blots or gels/Fig3-F/Caspase9-2.tif]

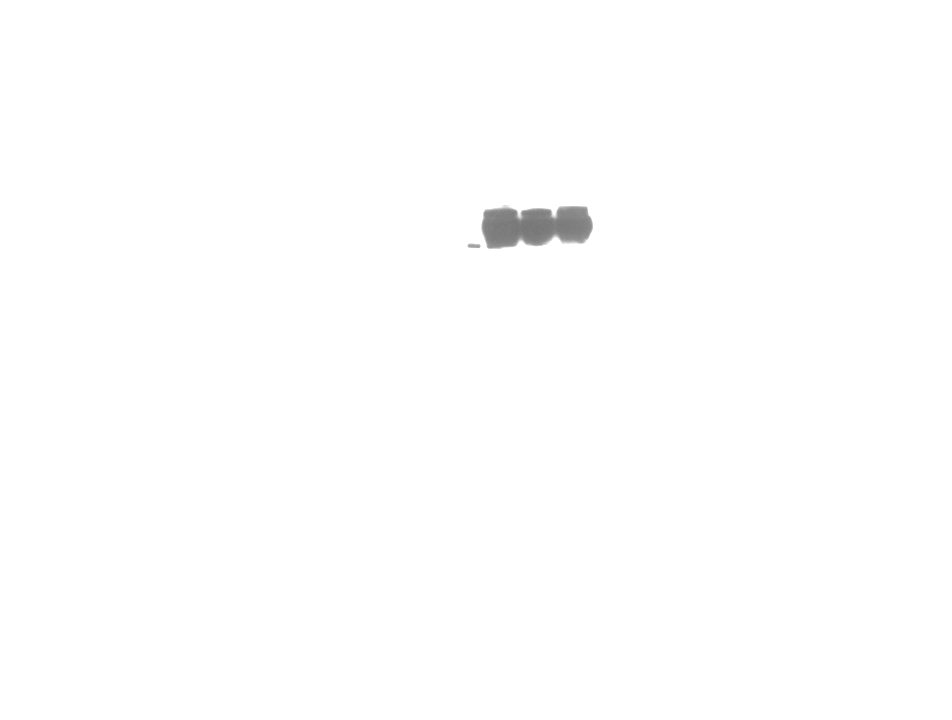

Supplement: Supplementary file 1 [file cancers-14-05976-s001.zip › Original images for blots or gels/Fig3-F/Caspase9-3.tif]

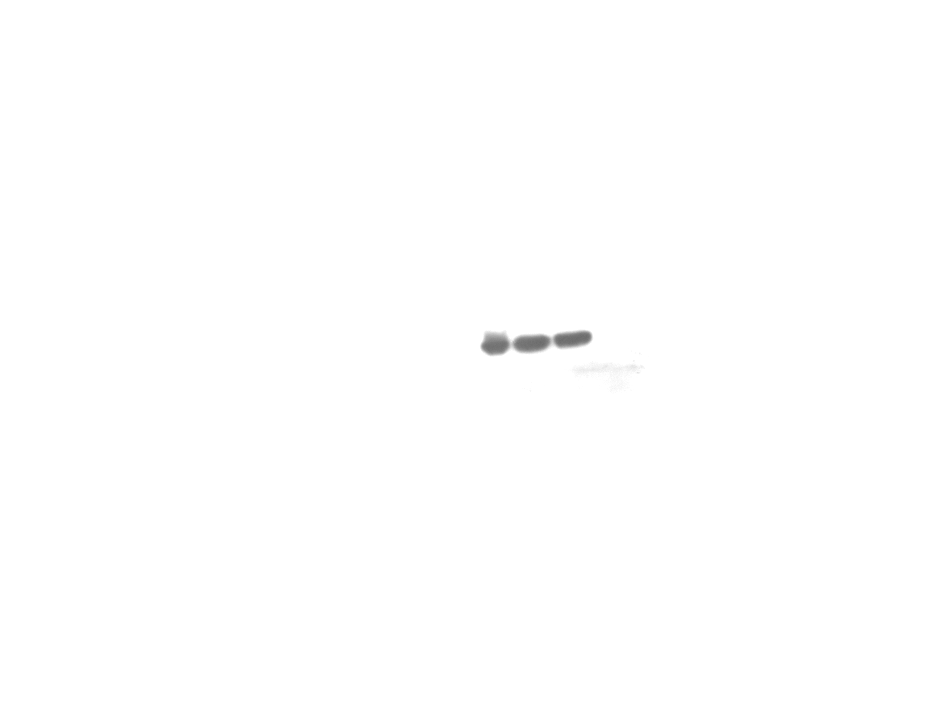

Supplement: Supplementary file 1 [file cancers-14-05976-s001.zip › Original images for blots or gels/Fig3-F/GAPDH-1.tif]

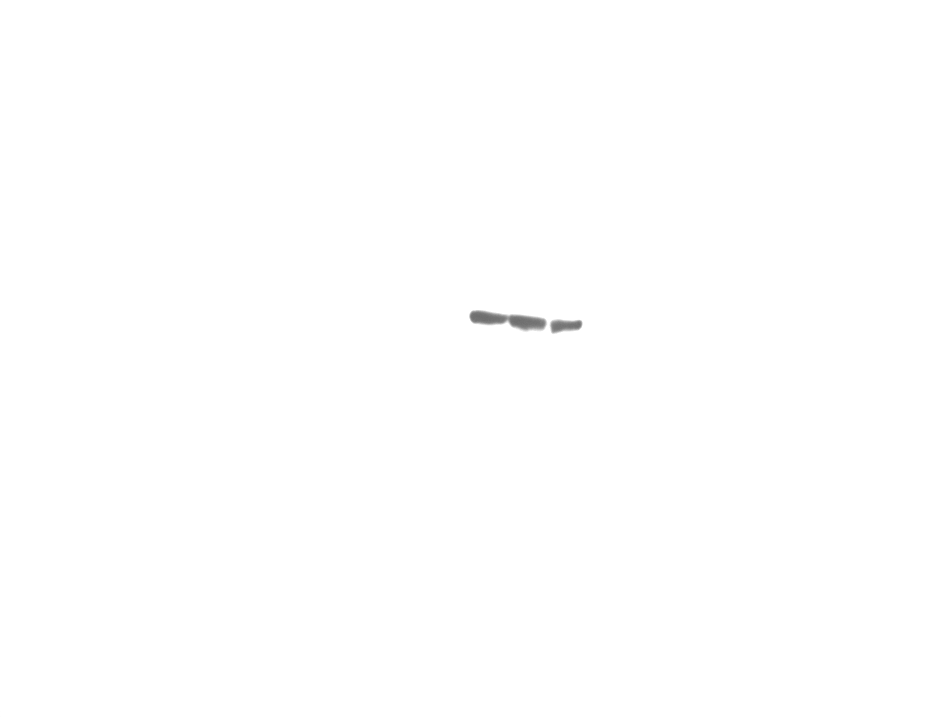

Supplement: Supplementary file 1 [file cancers-14-05976-s001.zip › Original images for blots or gels/Fig3-F/GAPDH-2.tif]

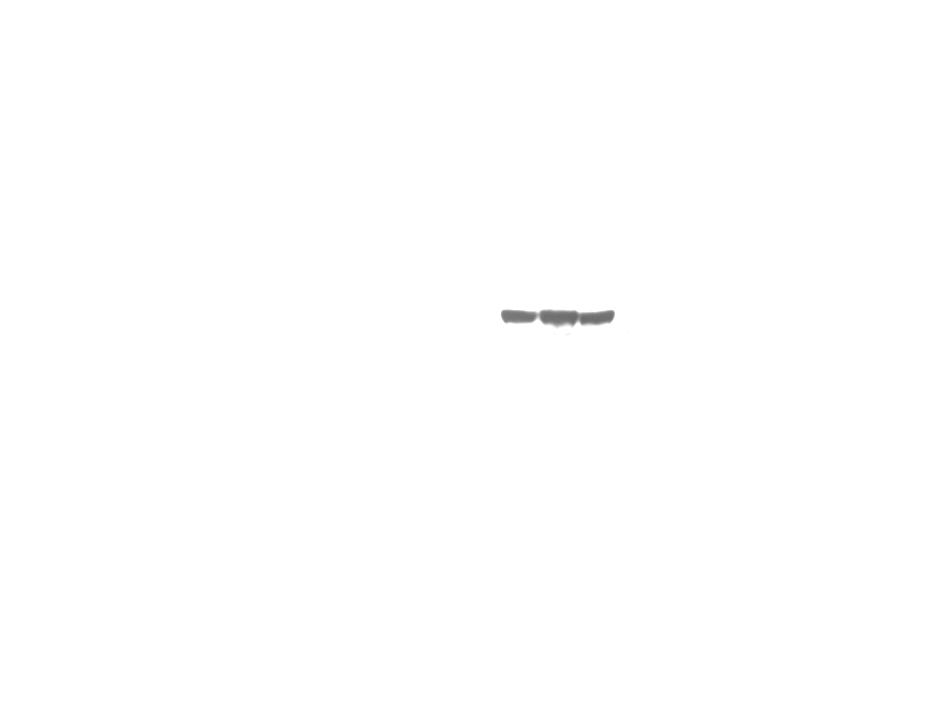

Supplement: Supplementary file 1 [file cancers-14-05976-s001.zip › Original images for blots or gels/Fig3-F/GAPDH-3.tif]

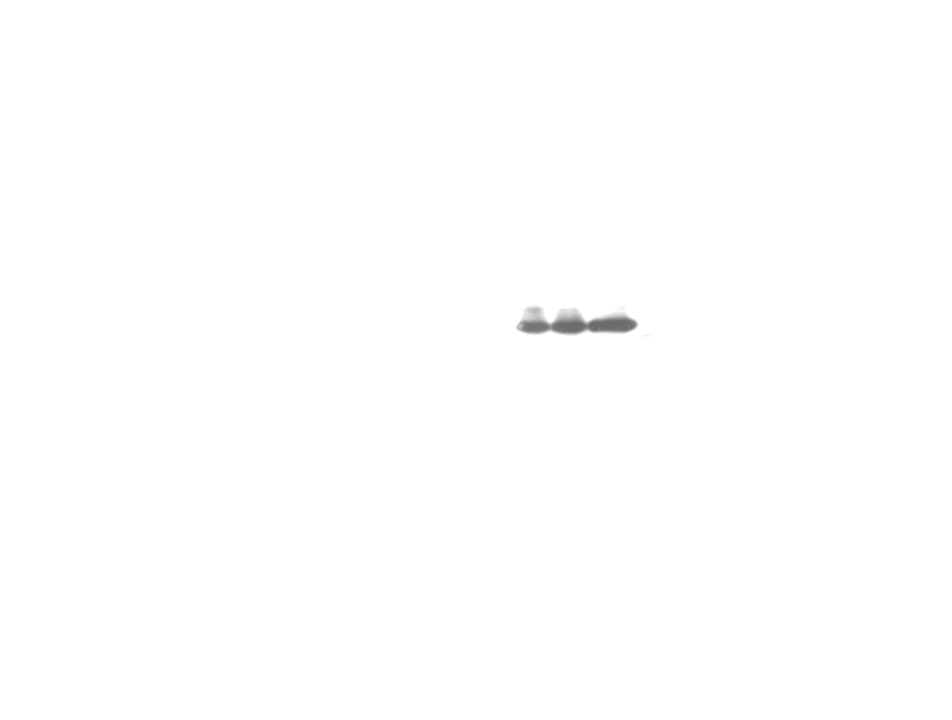

Supplement: Supplementary file 1 [file cancers-14-05976-s001.zip › Original images for blots or gels/Fig3-G/GPX4-1.tif]

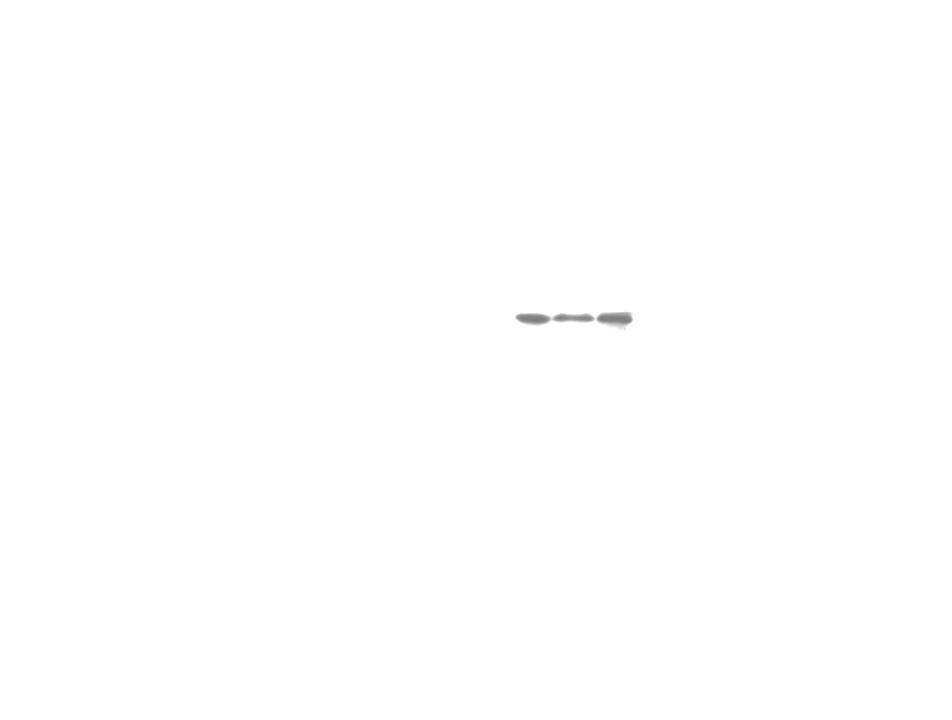

Supplement: Supplementary file 1 [file cancers-14-05976-s001.zip › Original images for blots or gels/Fig3-G/GPX4-2.tif]

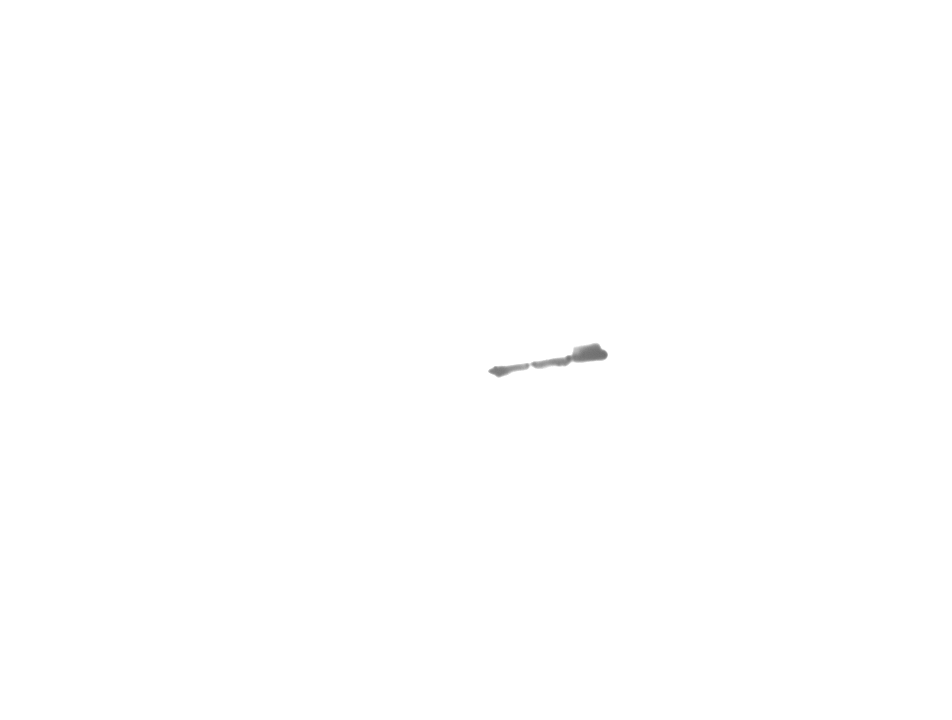

Supplement: Supplementary file 1 [file cancers-14-05976-s001.zip › Original images for blots or gels/Fig3-G/GPX4-3.tif]

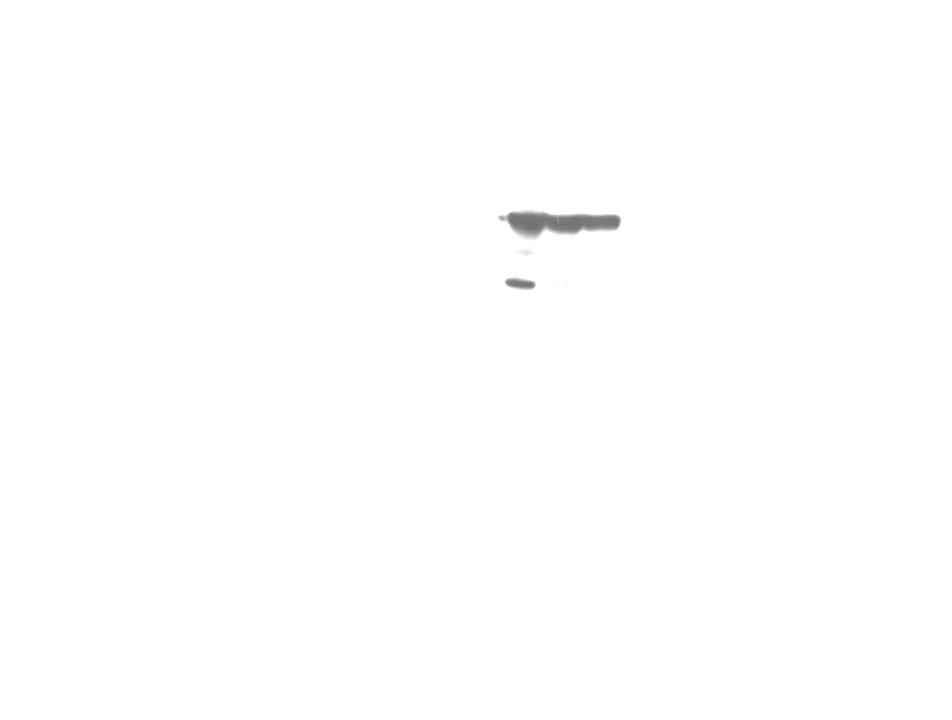

Supplement: Supplementary file 1 [file cancers-14-05976-s001.zip › Original images for blots or gels/Fig3-G/GSDMD-1.tif]

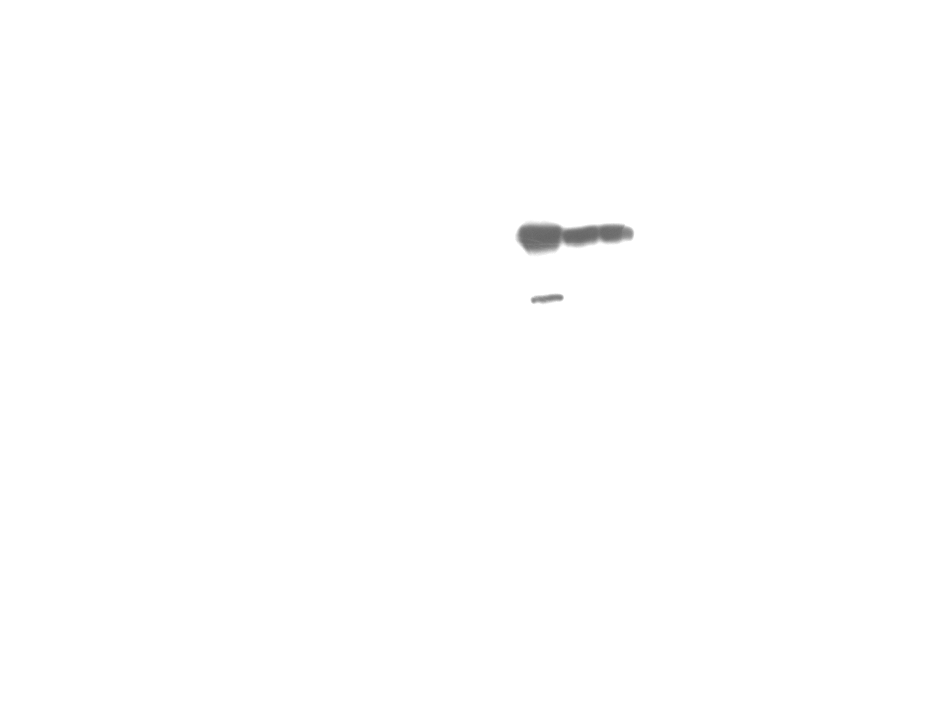

Supplement: Supplementary file 1 [file cancers-14-05976-s001.zip › Original images for blots or gels/Fig3-G/GSDMD-2.tif]

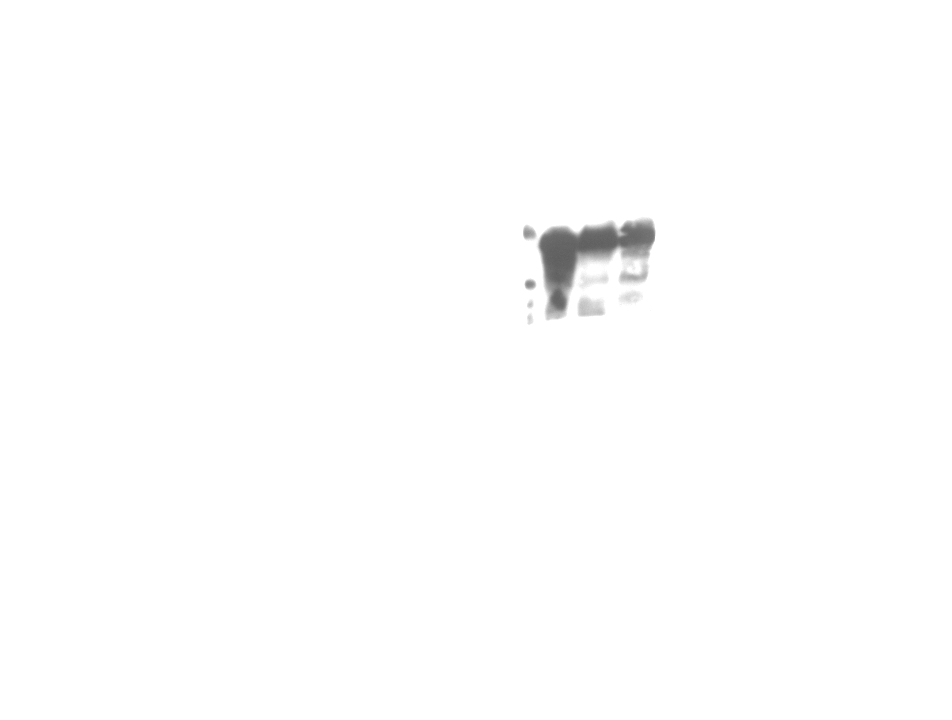

Supplement: Supplementary file 1 [file cancers-14-05976-s001.zip › Original images for blots or gels/Fig3-G/GSDMD-3.tif]

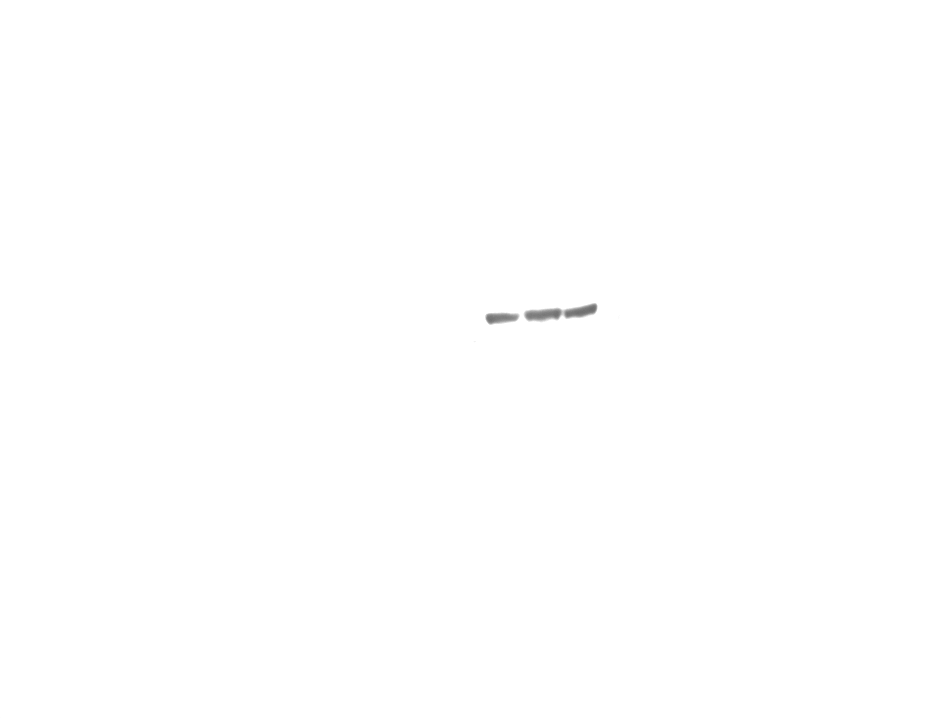

Supplement: Supplementary file 1 [file cancers-14-05976-s001.zip › Original images for blots or gels/Fig4-B/GAPDH-1.tif]

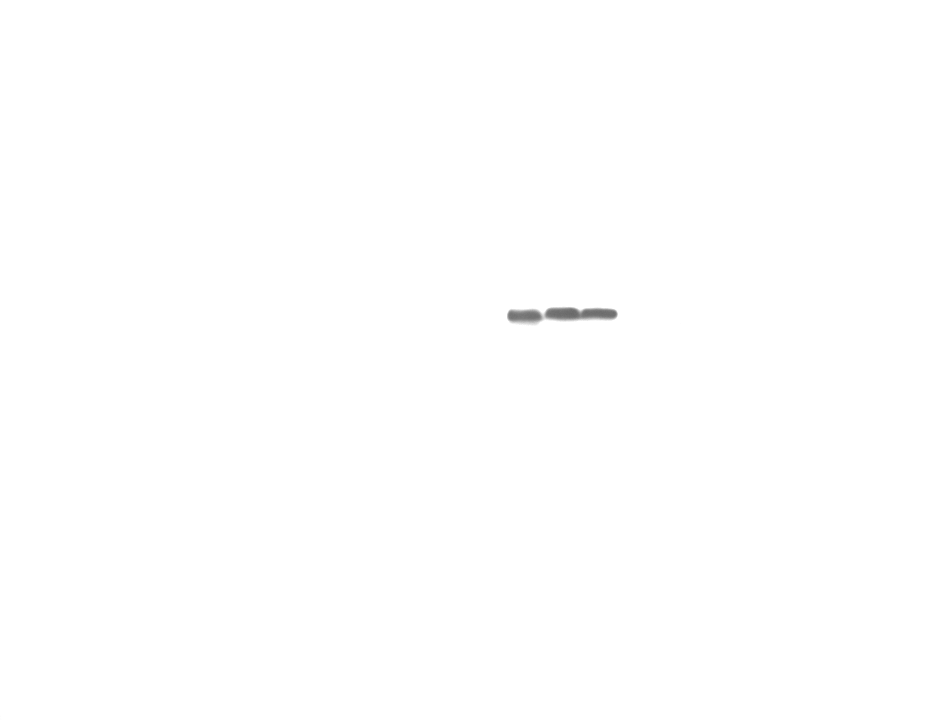

Supplement: Supplementary file 1 [file cancers-14-05976-s001.zip › Original images for blots or gels/Fig4-B/GAPDH-2.tif]

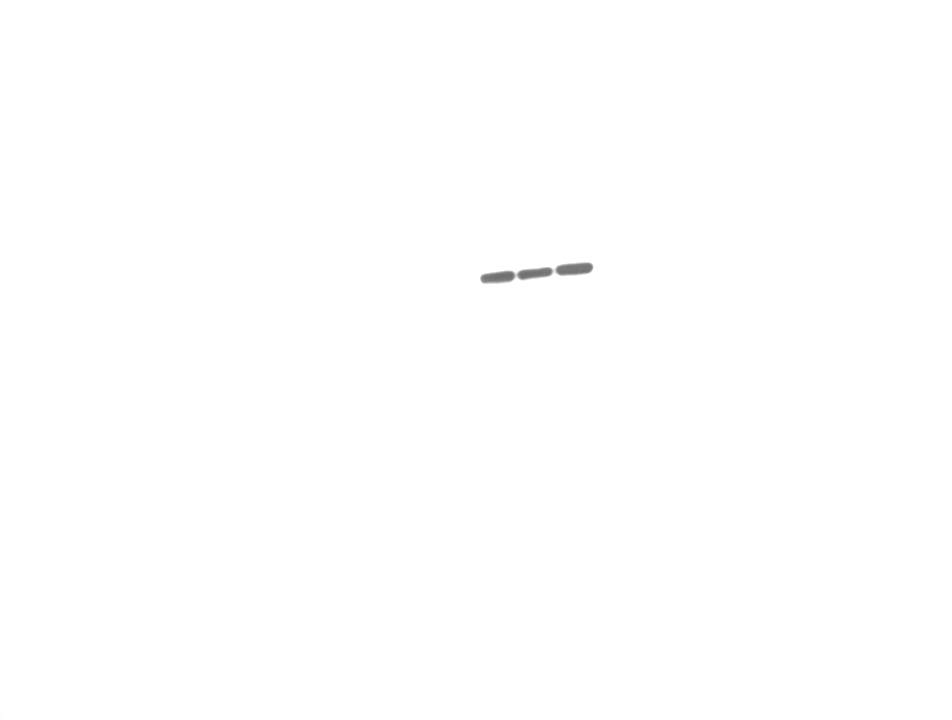

Supplement: Supplementary file 1 [file cancers-14-05976-s001.zip › Original images for blots or gels/Fig4-B/GAPDH-3.tif]

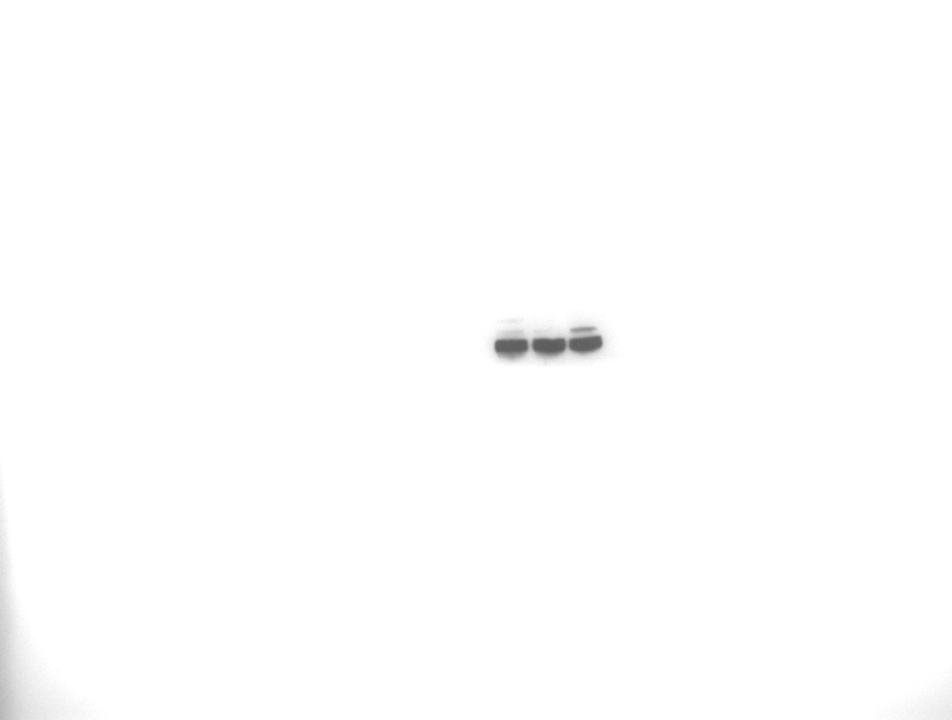

Supplement: Supplementary file 1 [file cancers-14-05976-s001.zip › Original images for blots or gels/Fig4-B/MLKL-1.tif]

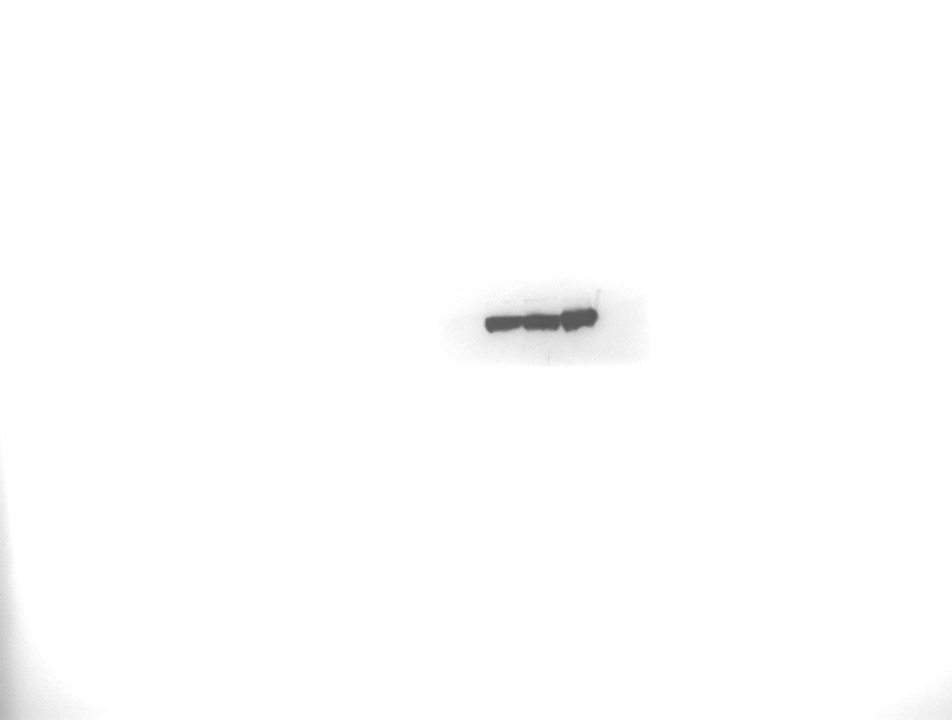

Supplement: Supplementary file 1 [file cancers-14-05976-s001.zip › Original images for blots or gels/Fig4-B/MLKL-2.tif]

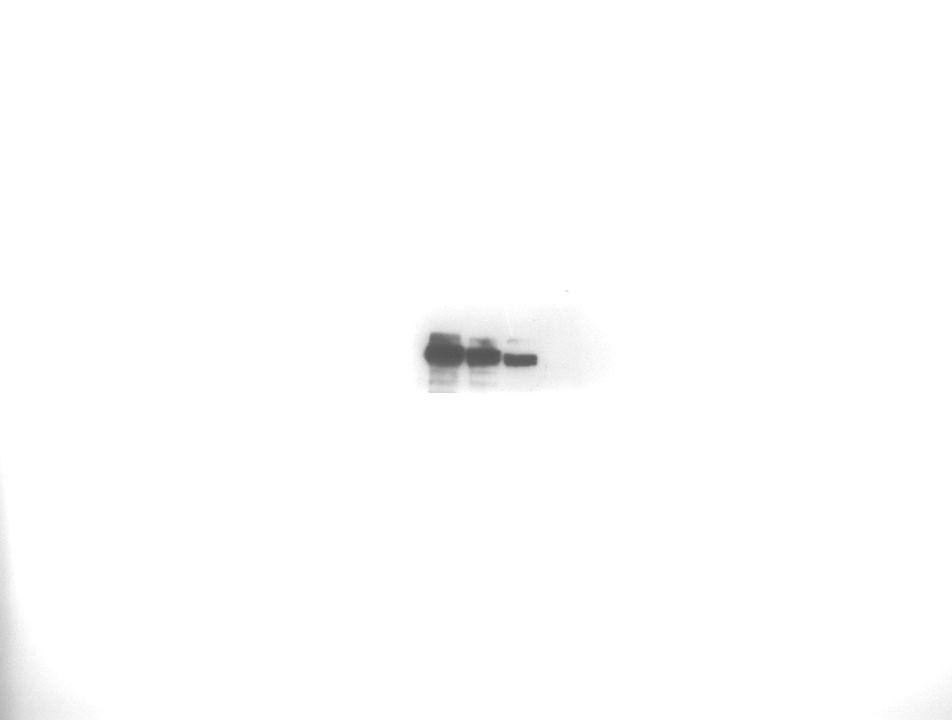

Supplement: Supplementary file 1 [file cancers-14-05976-s001.zip › Original images for blots or gels/Fig4-B/MLKL-3.tif]

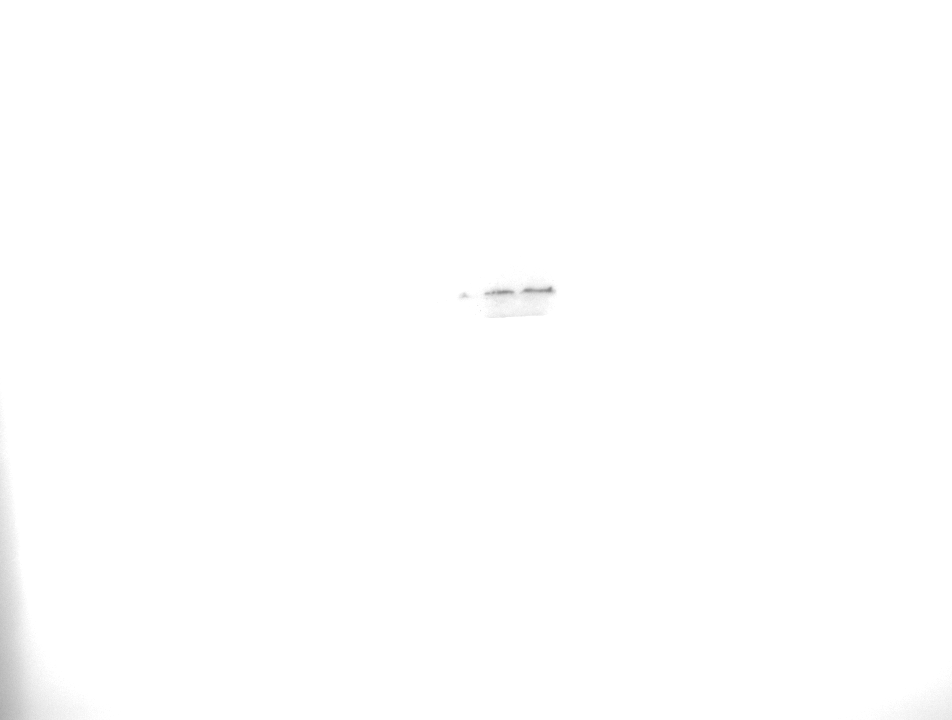

Supplement: Supplementary file 1 [file cancers-14-05976-s001.zip › Original images for blots or gels/Fig4-B/p-MLKL-1.tif]

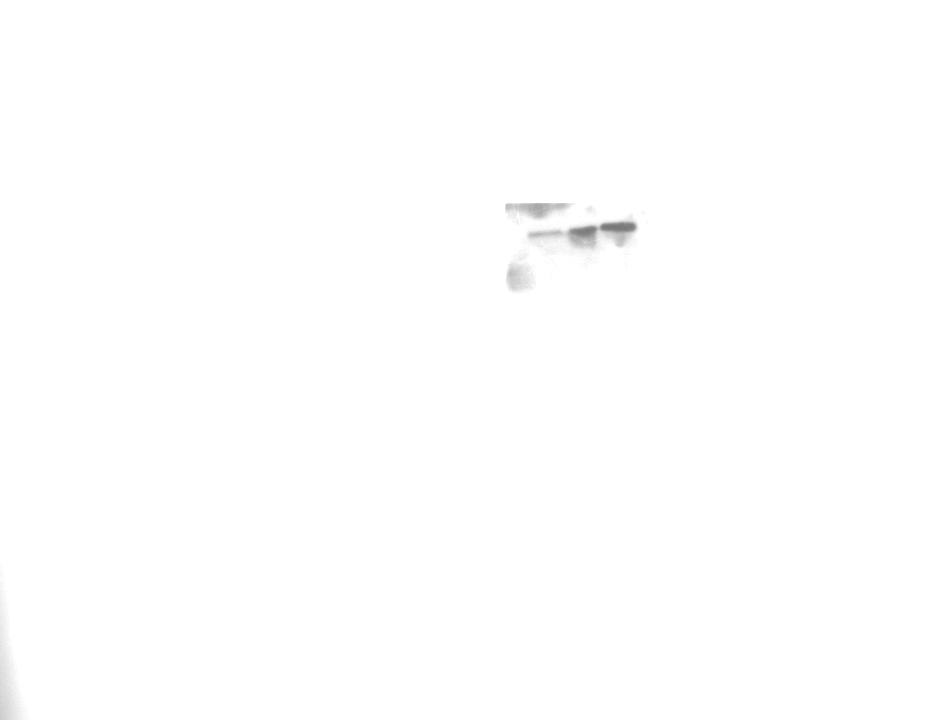

Supplement: Supplementary file 1 [file cancers-14-05976-s001.zip › Original images for blots or gels/Fig4-B/p-MLKL-2.tif]

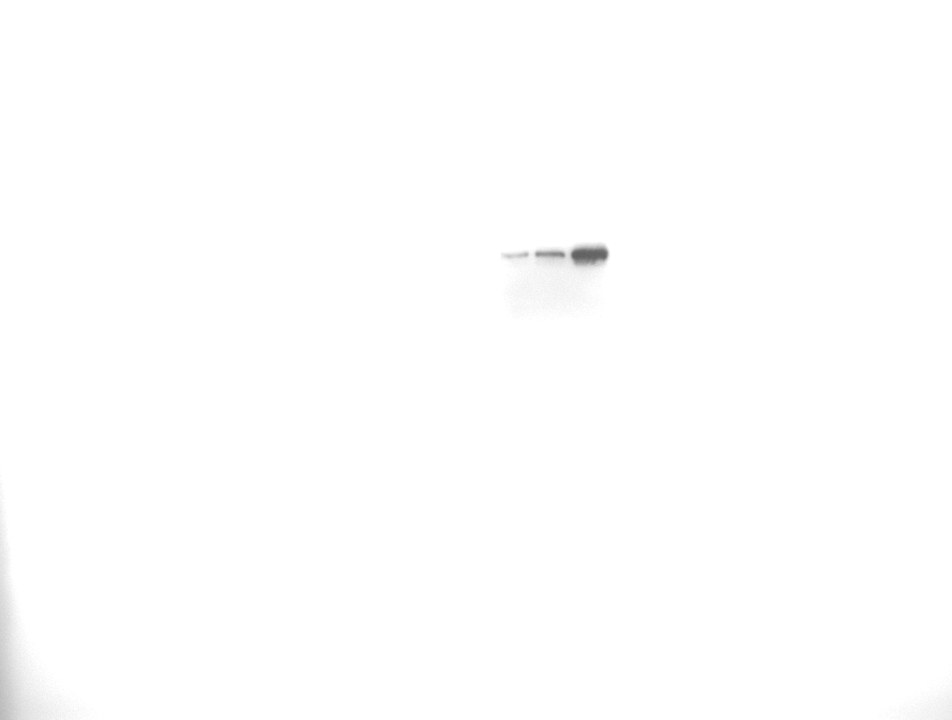

Supplement: Supplementary file 1 [file cancers-14-05976-s001.zip › Original images for blots or gels/Fig4-B/p-MLKL-3.tif]

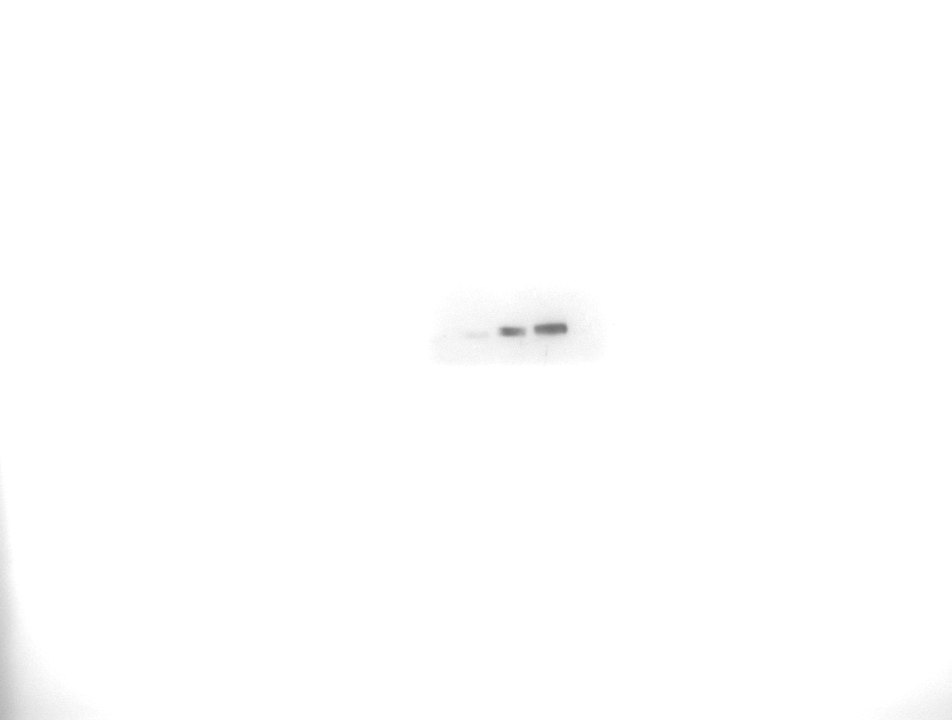

Supplement: Supplementary file 1 [file cancers-14-05976-s001.zip › Original images for blots or gels/Fig4-B/p-RIP1-1.tif]

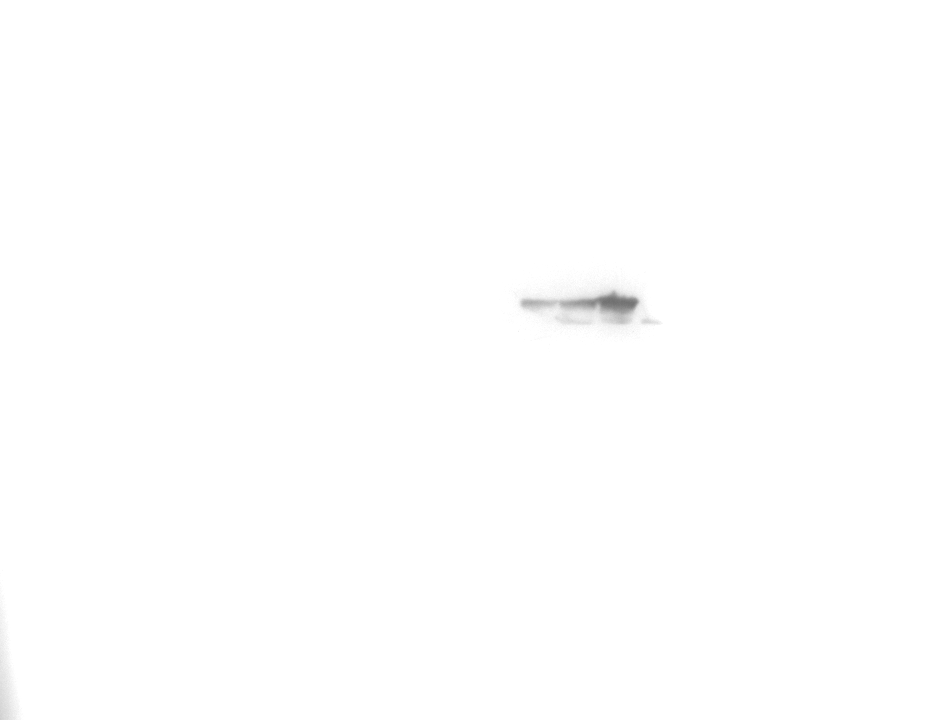

Supplement: Supplementary file 1 [file cancers-14-05976-s001.zip › Original images for blots or gels/Fig4-B/p-RIP1-2.tif]

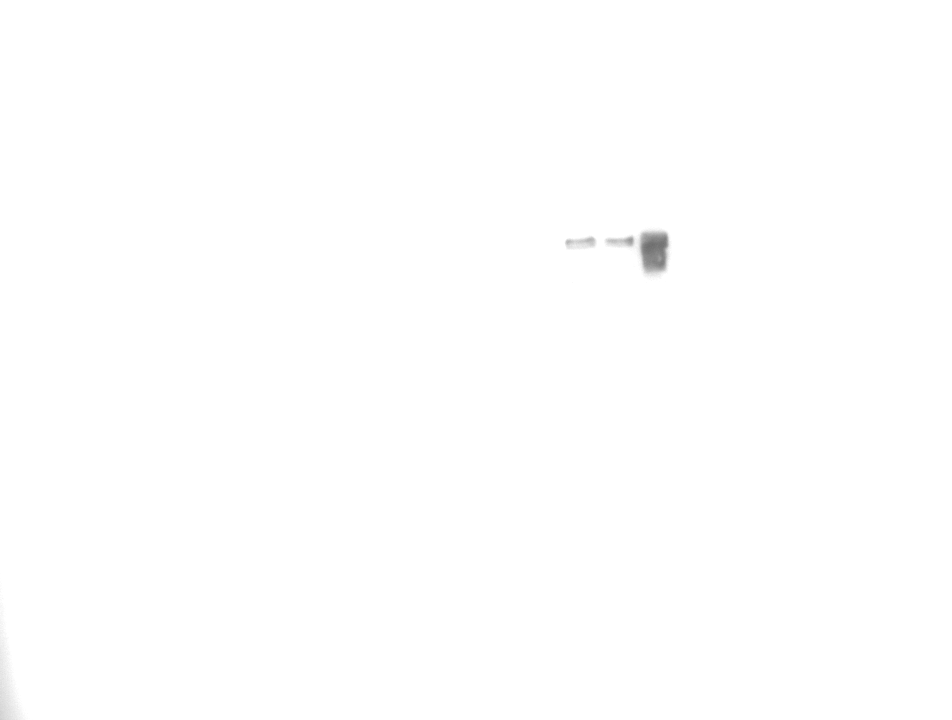

Supplement: Supplementary file 1 [file cancers-14-05976-s001.zip › Original images for blots or gels/Fig4-B/p-RIP1-3.tif]

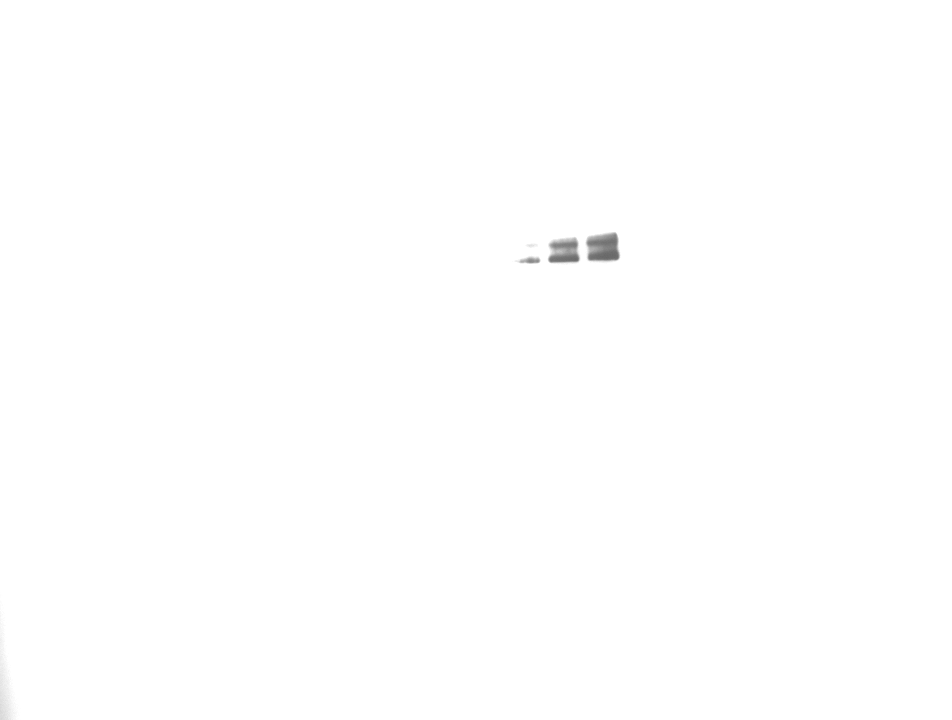

Supplement: Supplementary file 1 [file cancers-14-05976-s001.zip › Original images for blots or gels/Fig4-B/p-RIP3-1.tif]

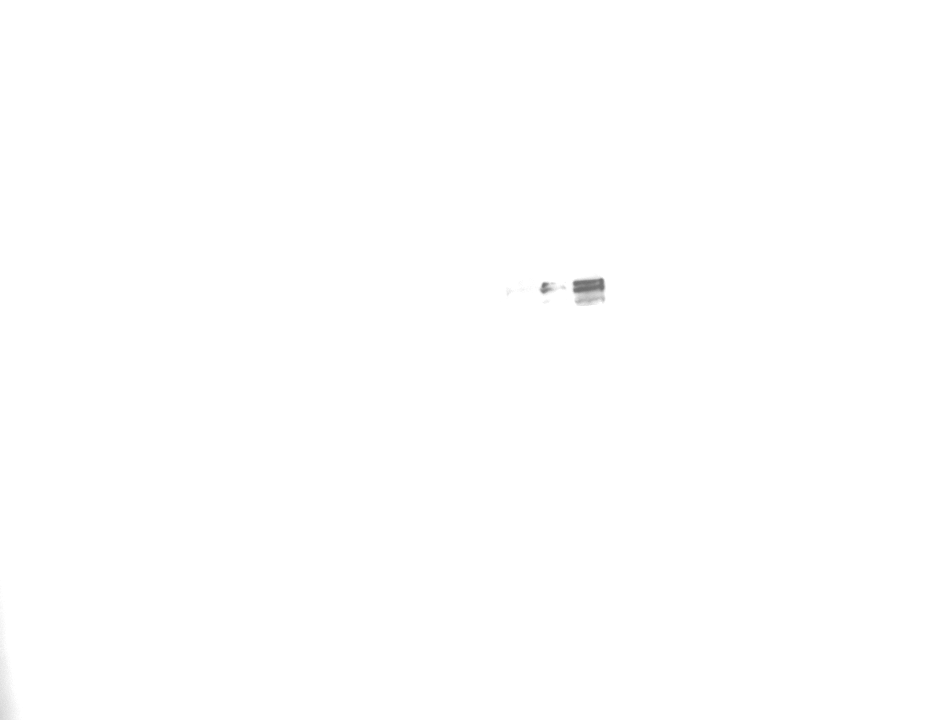

Supplement: Supplementary file 1 [file cancers-14-05976-s001.zip › Original images for blots or gels/Fig4-B/p-RIP3-2.tif]

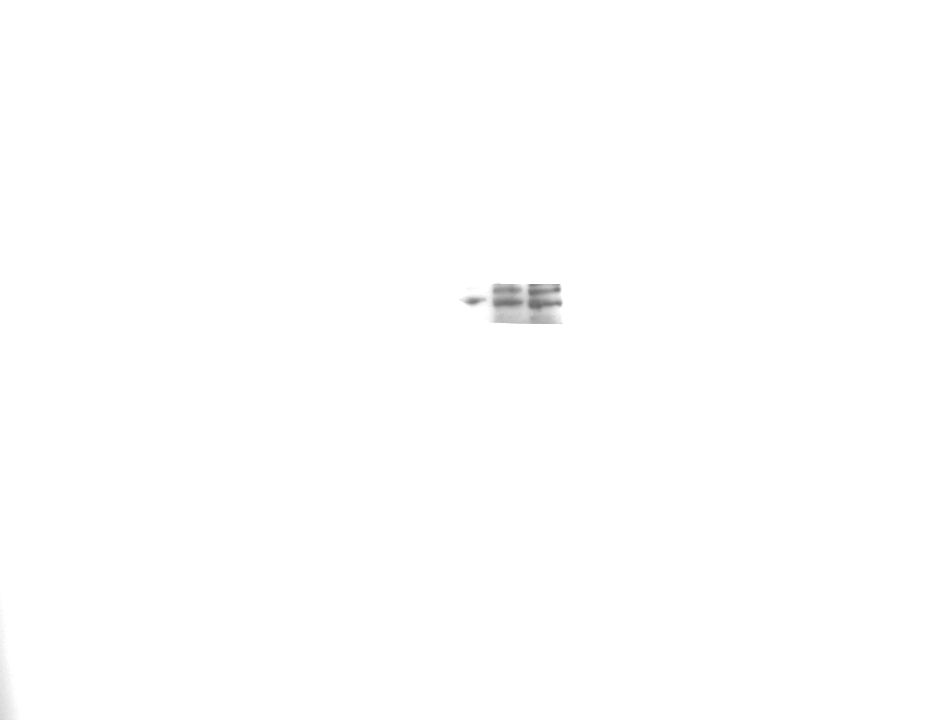

Supplement: Supplementary file 1 [file cancers-14-05976-s001.zip › Original images for blots or gels/Fig4-B/p-RIP3-3.tif]

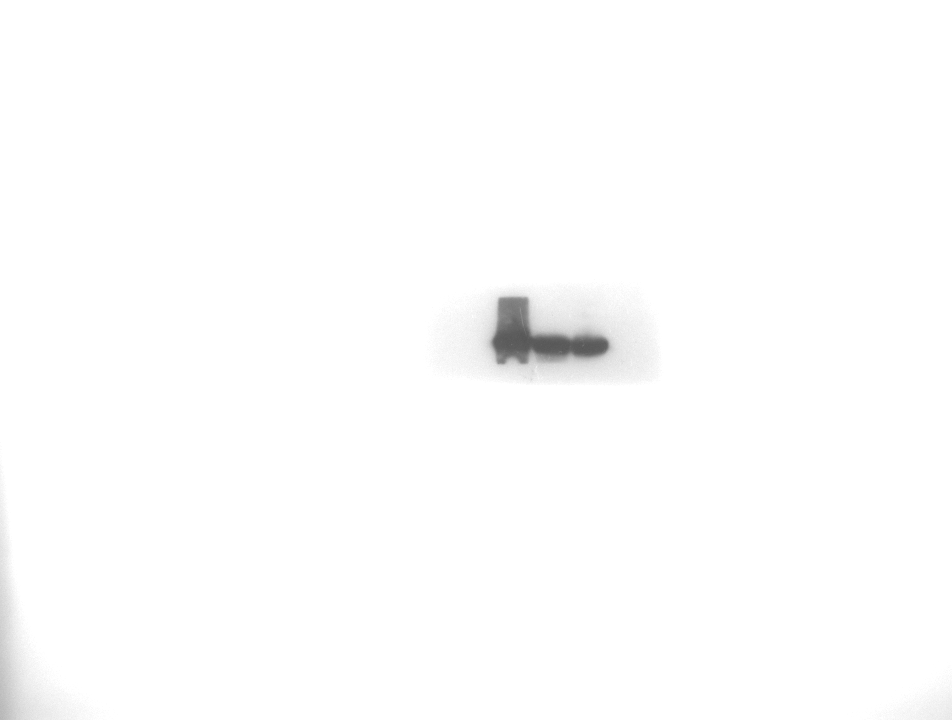

Supplement: Supplementary file 1 [file cancers-14-05976-s001.zip › Original images for blots or gels/Fig4-B/RIP1-1.tif]

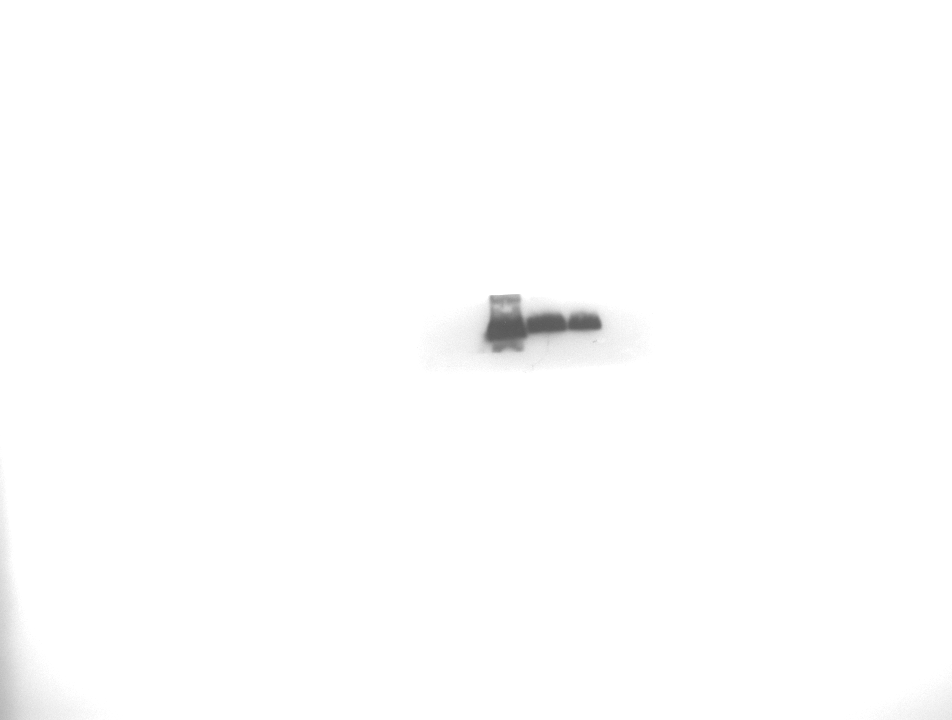

Supplement: Supplementary file 1 [file cancers-14-05976-s001.zip › Original images for blots or gels/Fig4-B/RIP1-2.tif]

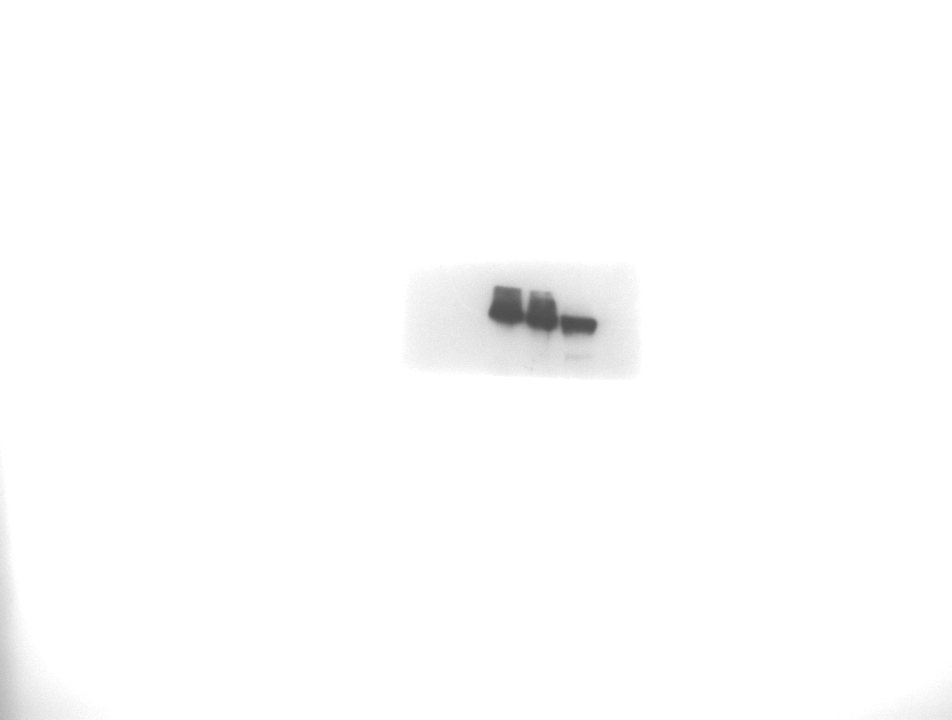

Supplement: Supplementary file 1 [file cancers-14-05976-s001.zip › Original images for blots or gels/Fig4-B/RIP1-3.tif]

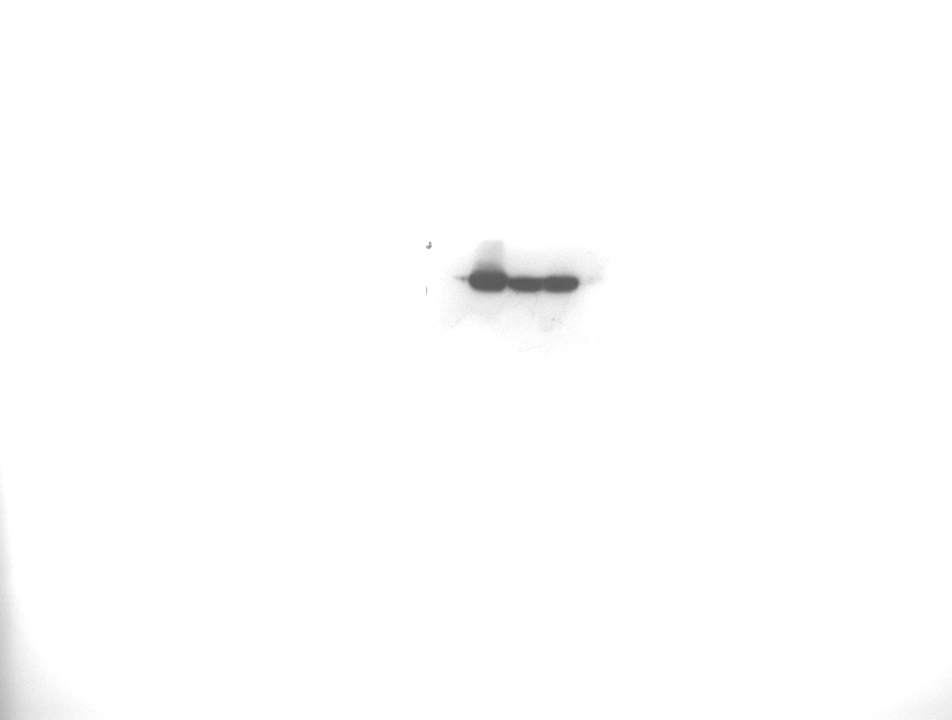

Supplement: Supplementary file 1 [file cancers-14-05976-s001.zip › Original images for blots or gels/Fig4-B/RIP3-1.tif]

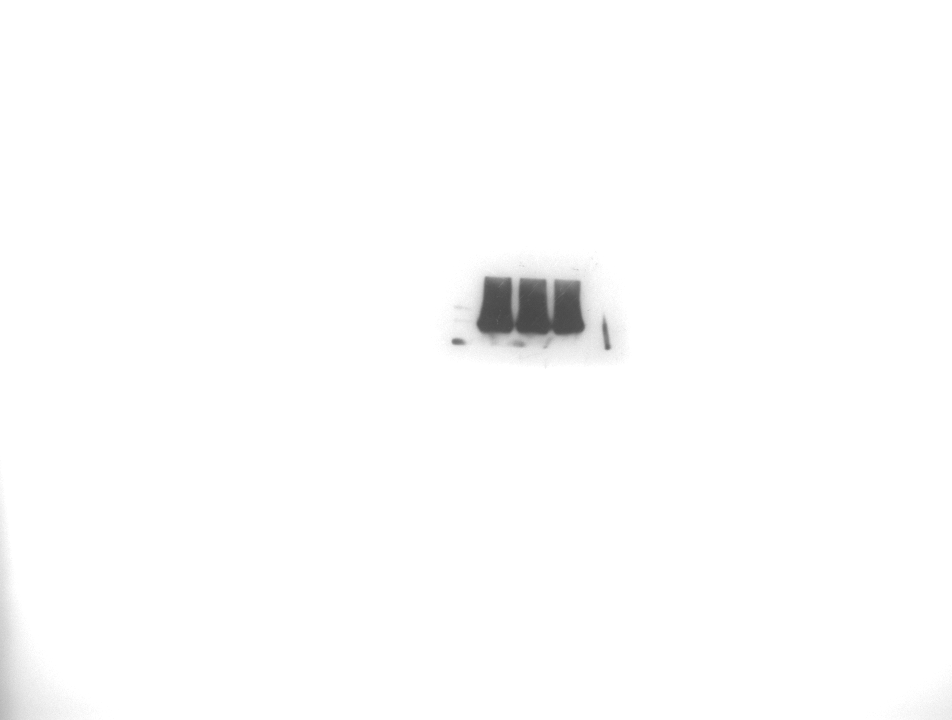

Supplement: Supplementary file 1 [file cancers-14-05976-s001.zip › Original images for blots or gels/Fig4-B/RIP3-2.tif]

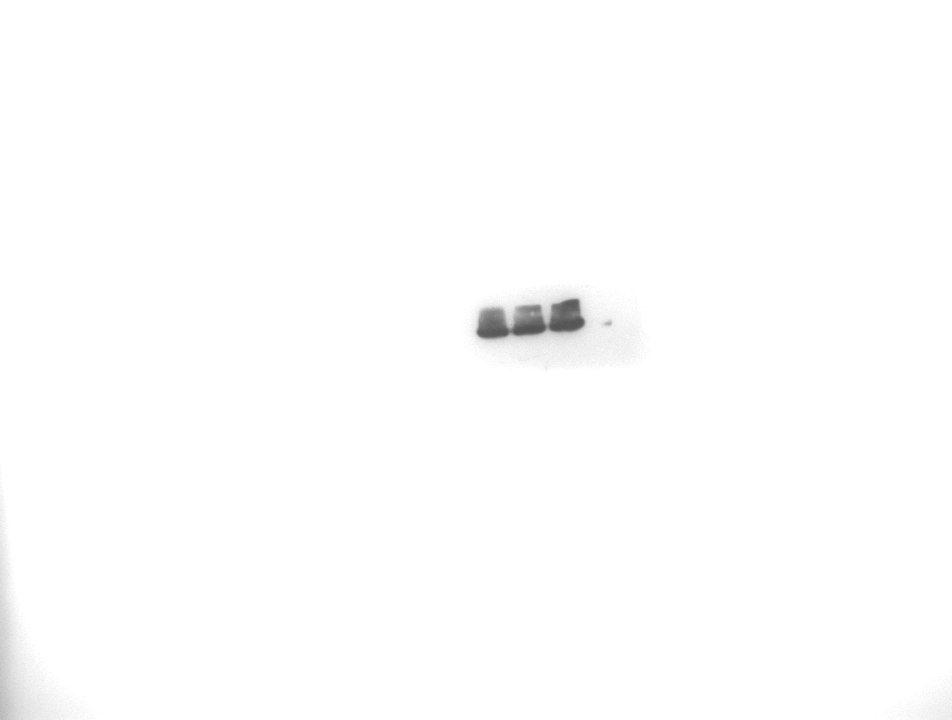

Supplement: Supplementary file 1 [file cancers-14-05976-s001.zip › Original images for blots or gels/Fig4-B/RIP3-3.tif]

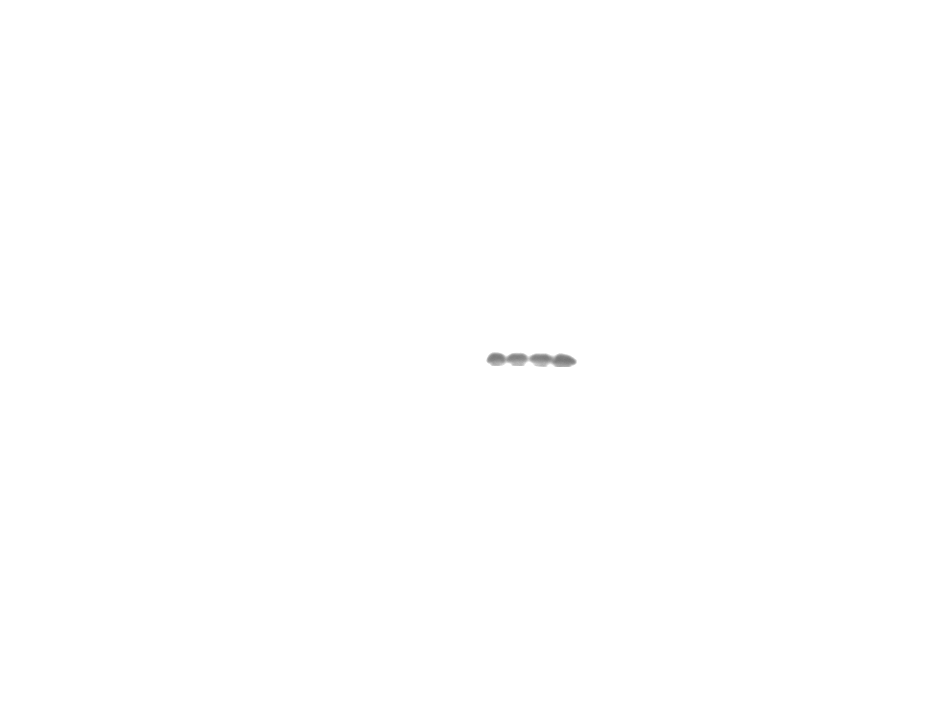

Supplement: Supplementary file 1 [file cancers-14-05976-s001.zip › Original images for blots or gels/Fig4-C/GAPDH-1.tif]

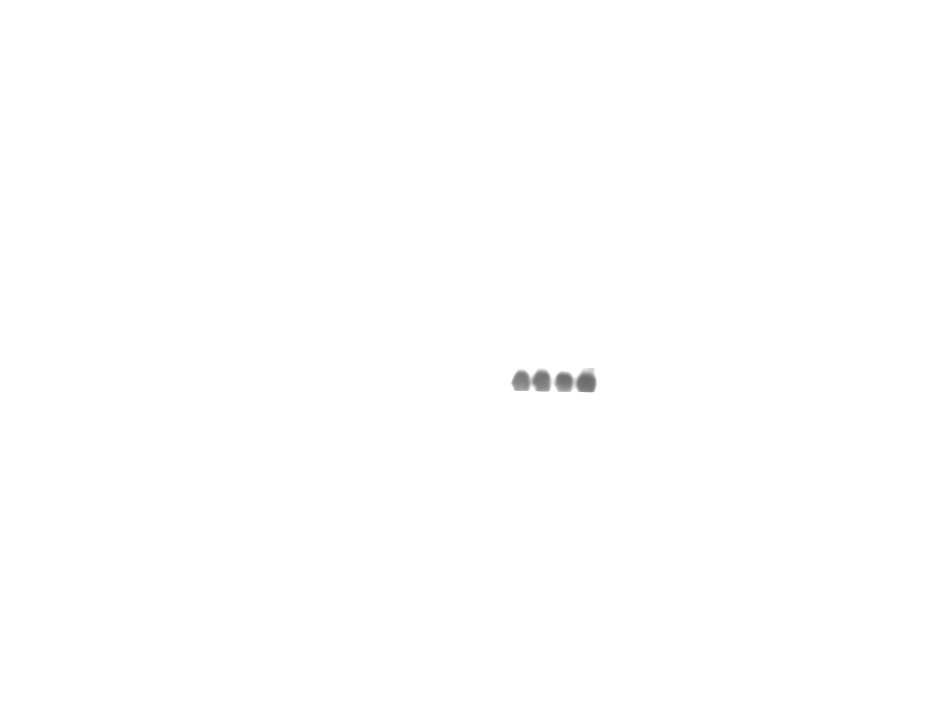

Supplement: Supplementary file 1 [file cancers-14-05976-s001.zip › Original images for blots or gels/Fig4-C/GAPDH-2.tif]

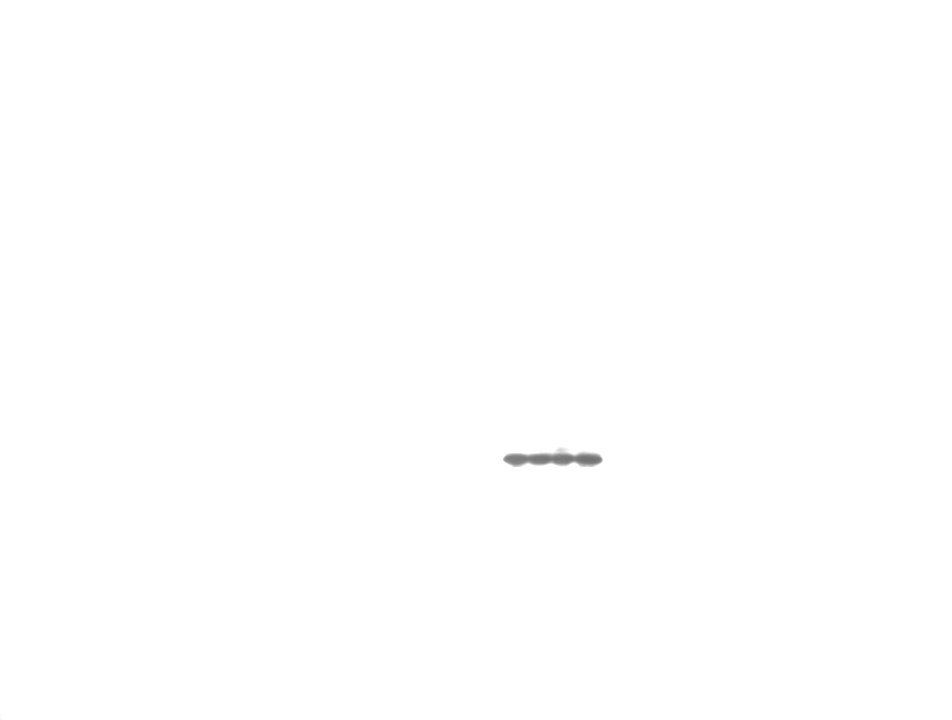

Supplement: Supplementary file 1 [file cancers-14-05976-s001.zip › Original images for blots or gels/Fig4-C/GAPDH-3.tif]

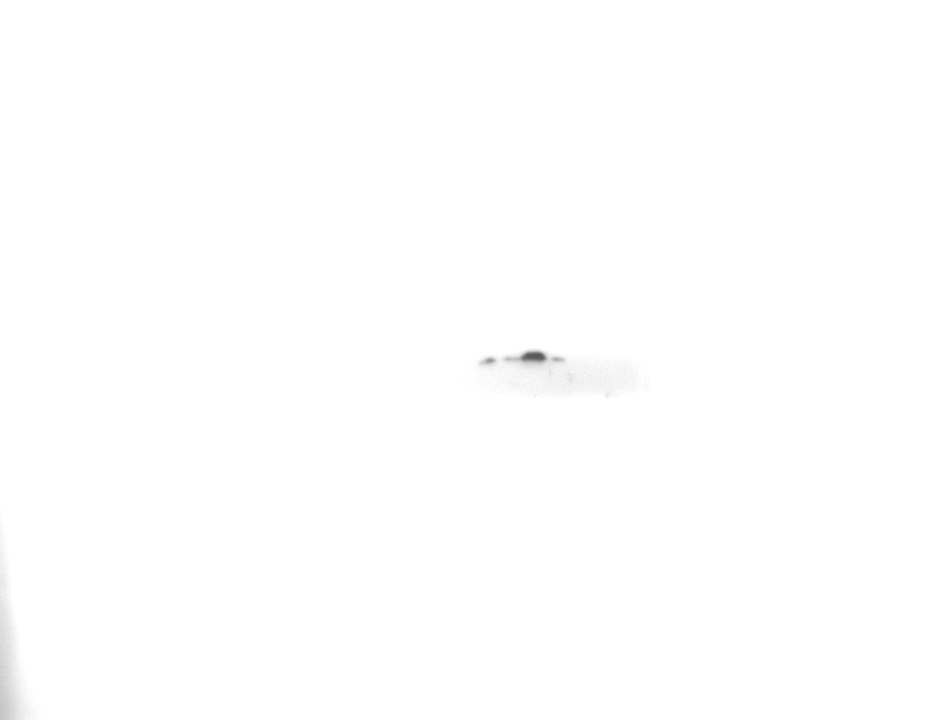

Supplement: Supplementary file 1 [file cancers-14-05976-s001.zip › Original images for blots or gels/Fig4-C/p-MLKL-1.tif]

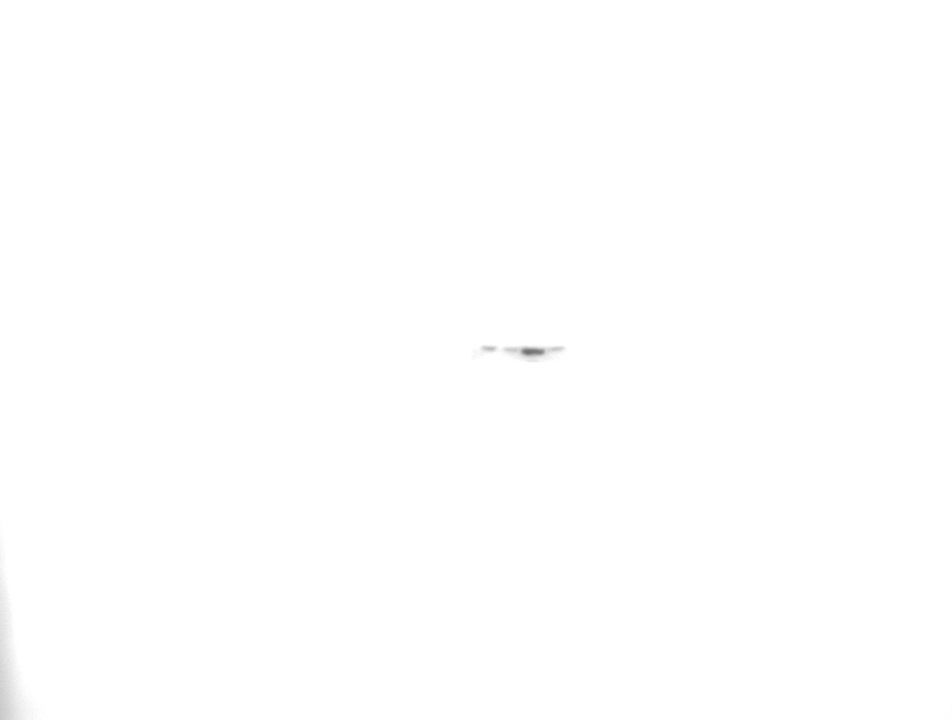

Supplement: Supplementary file 1 [file cancers-14-05976-s001.zip › Original images for blots or gels/Fig4-C/p-MLKL-2.tif]

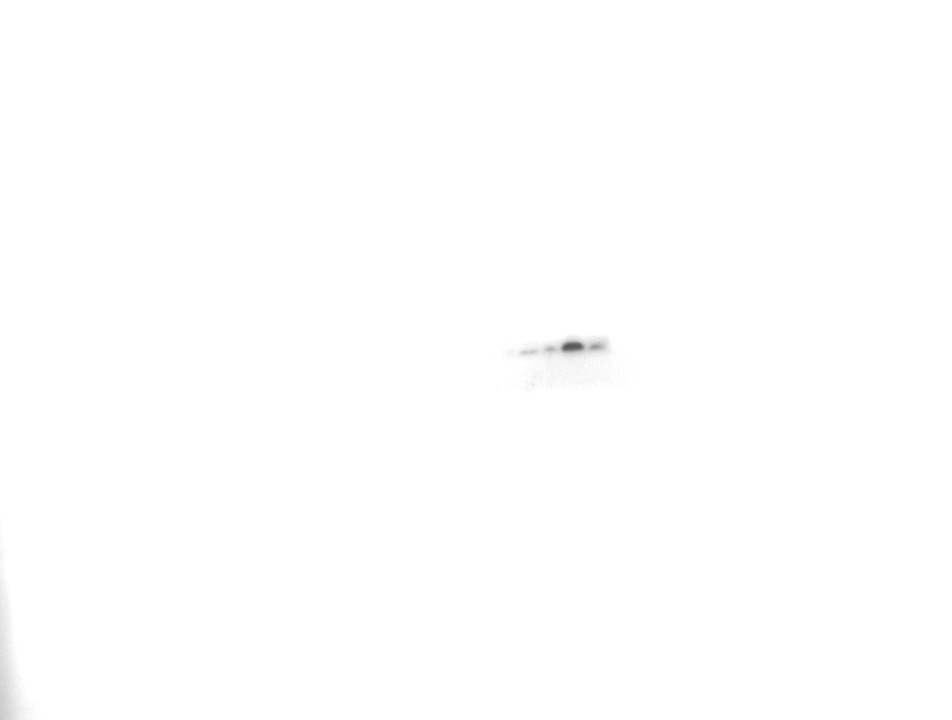

Supplement: Supplementary file 1 [file cancers-14-05976-s001.zip › Original images for blots or gels/Fig4-C/p-MLKL-3.tif]

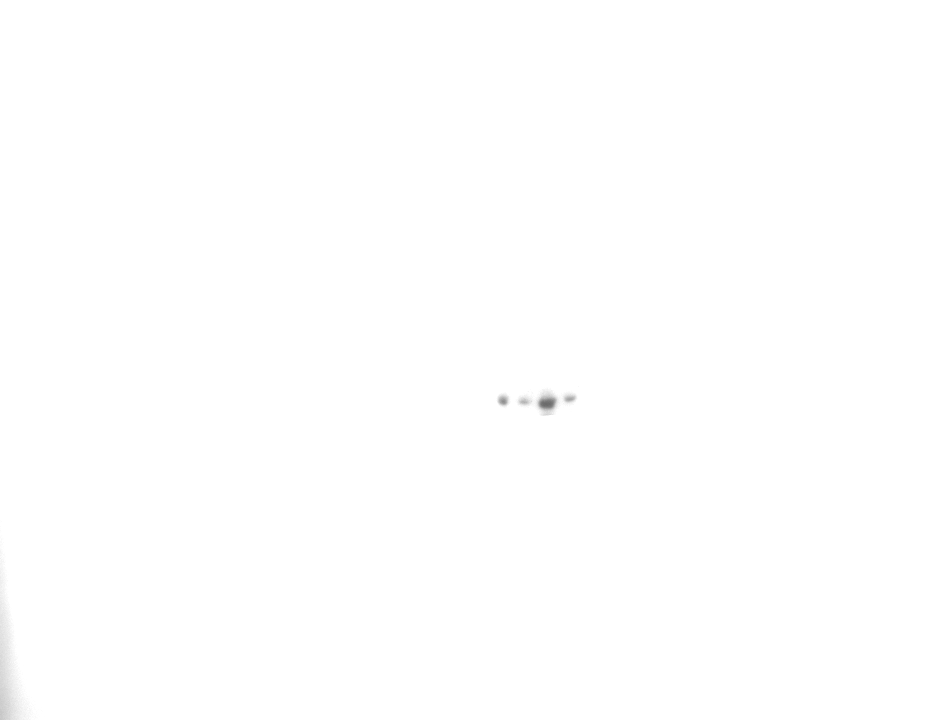

Supplement: Supplementary file 1 [file cancers-14-05976-s001.zip › Original images for blots or gels/Fig4-C/p-RIP1-1.tif]

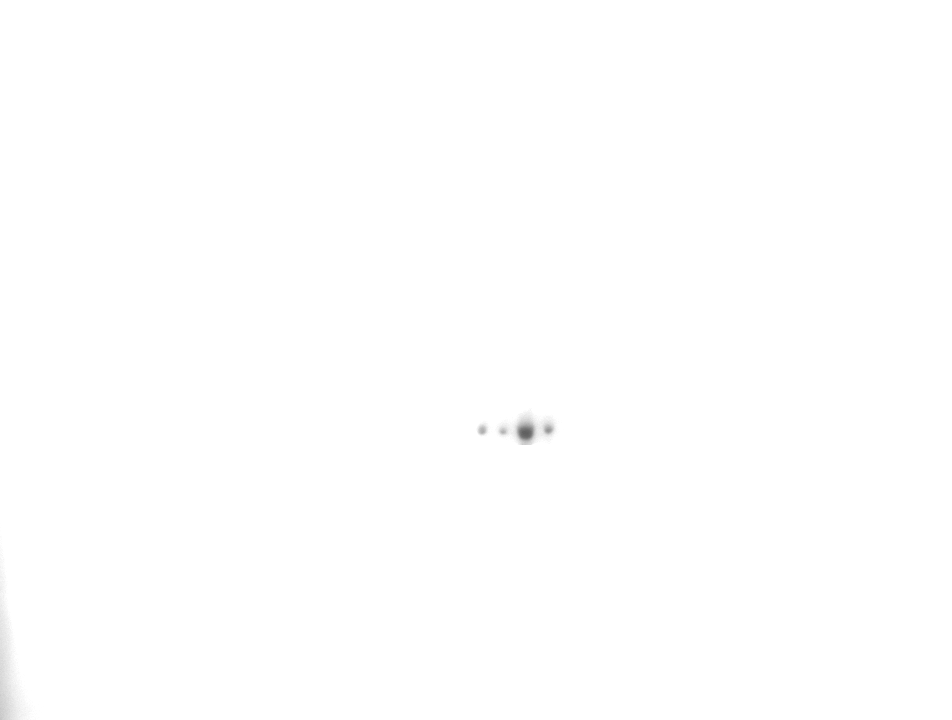

Supplement: Supplementary file 1 [file cancers-14-05976-s001.zip › Original images for blots or gels/Fig4-C/p-RIP1-2.tif]

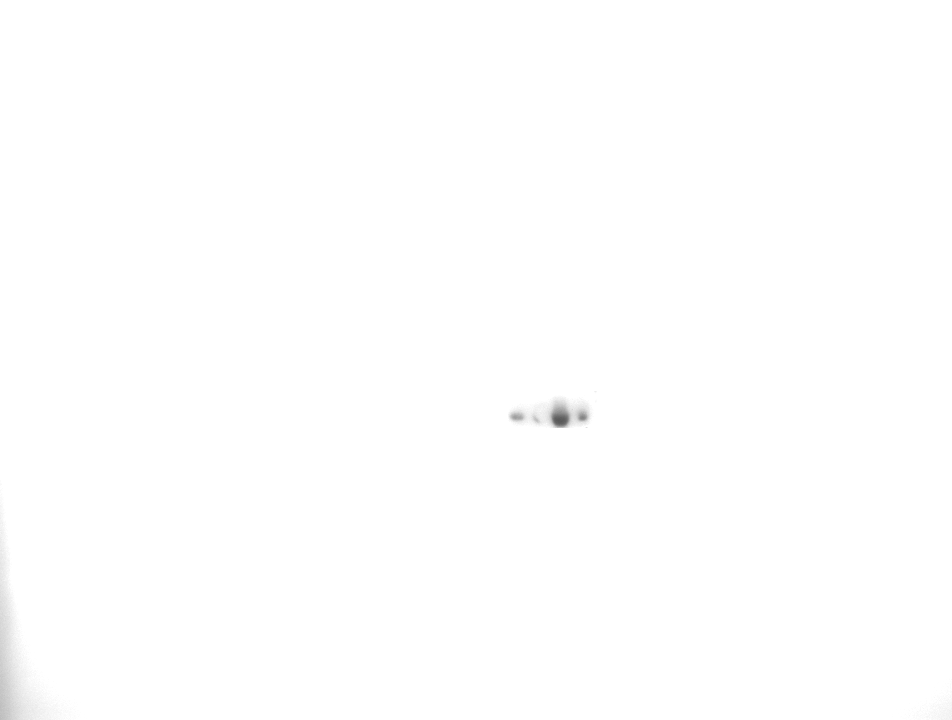

Supplement: Supplementary file 1 [file cancers-14-05976-s001.zip › Original images for blots or gels/Fig4-C/p-RIP1-3.tif]

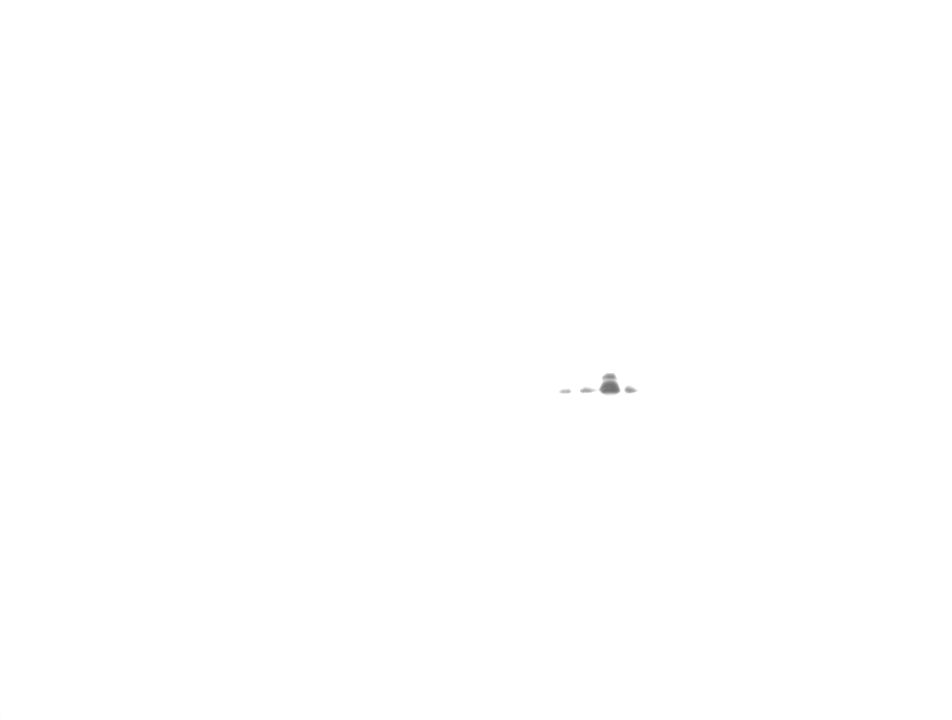

Supplement: Supplementary file 1 [file cancers-14-05976-s001.zip › Original images for blots or gels/Fig4-C/p-RIP3-1.tif]

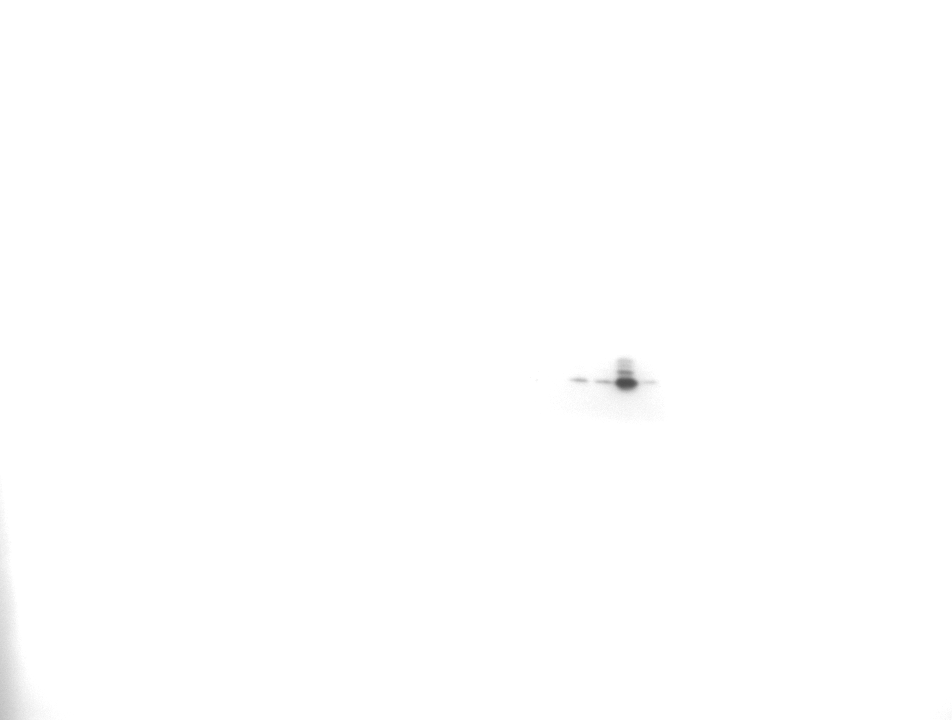

Supplement: Supplementary file 1 [file cancers-14-05976-s001.zip › Original images for blots or gels/Fig4-C/p-RIP3-2.tif]

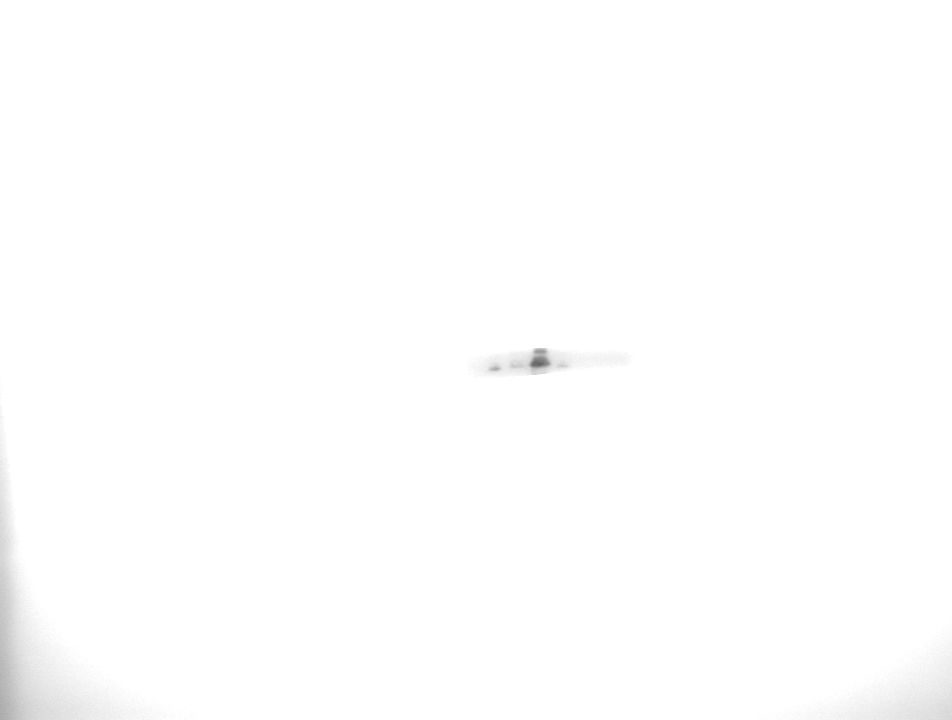

Supplement: Supplementary file 1 [file cancers-14-05976-s001.zip › Original images for blots or gels/Fig4-C/p-RIP3-3.tif]

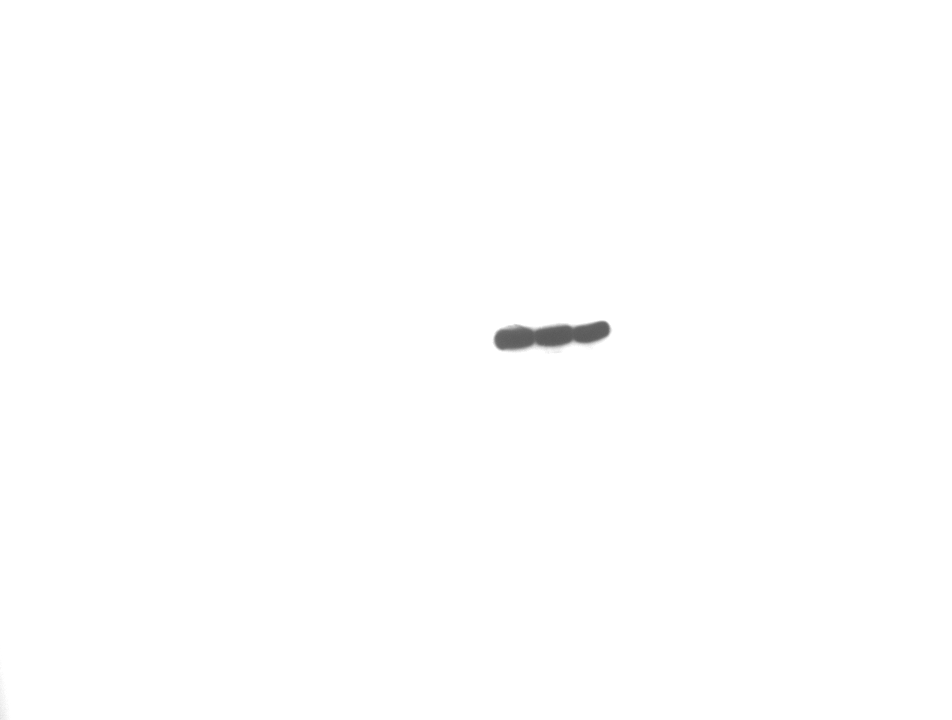

Supplement: Supplementary file 1 [file cancers-14-05976-s001.zip › Original images for blots or gels/Fig5-C/AKT-1.tif]

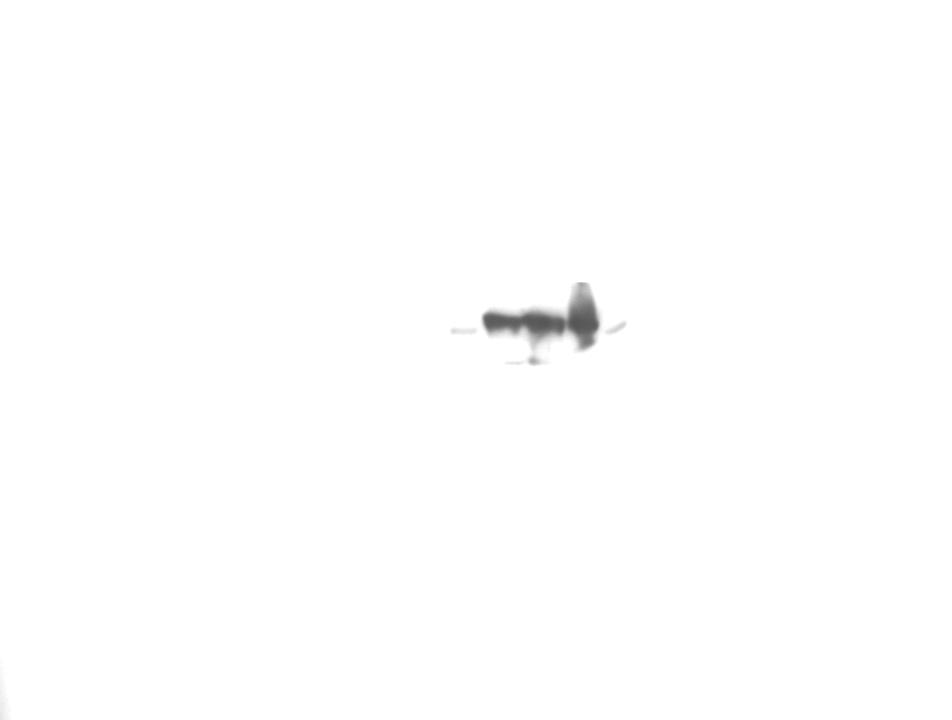

Supplement: Supplementary file 1 [file cancers-14-05976-s001.zip › Original images for blots or gels/Fig5-C/AKT-2.tif]

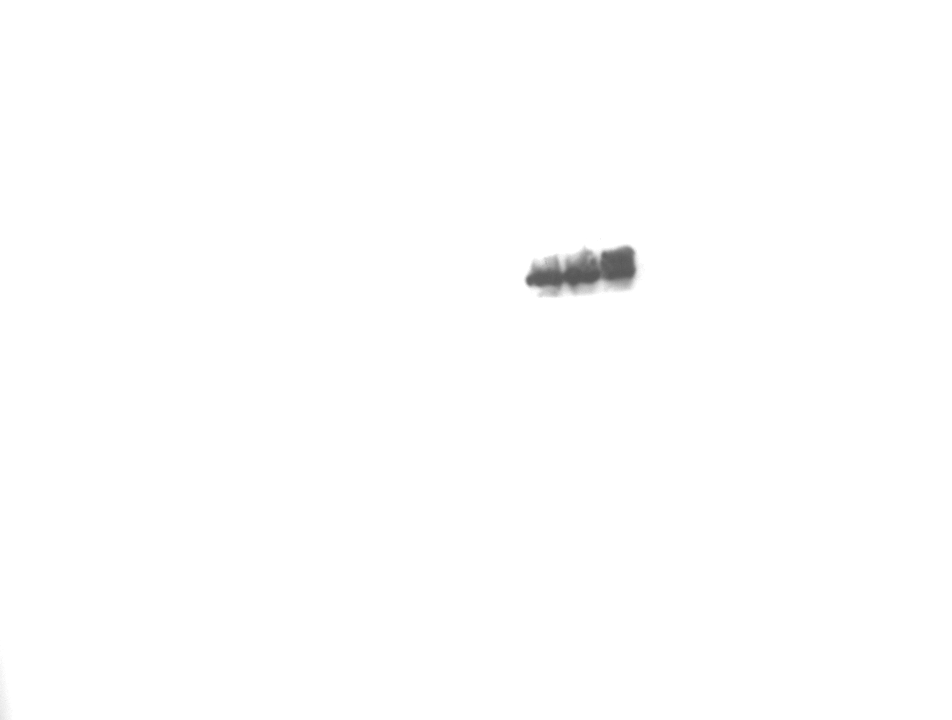

Supplement: Supplementary file 1 [file cancers-14-05976-s001.zip › Original images for blots or gels/Fig5-C/AKT-3.tif]

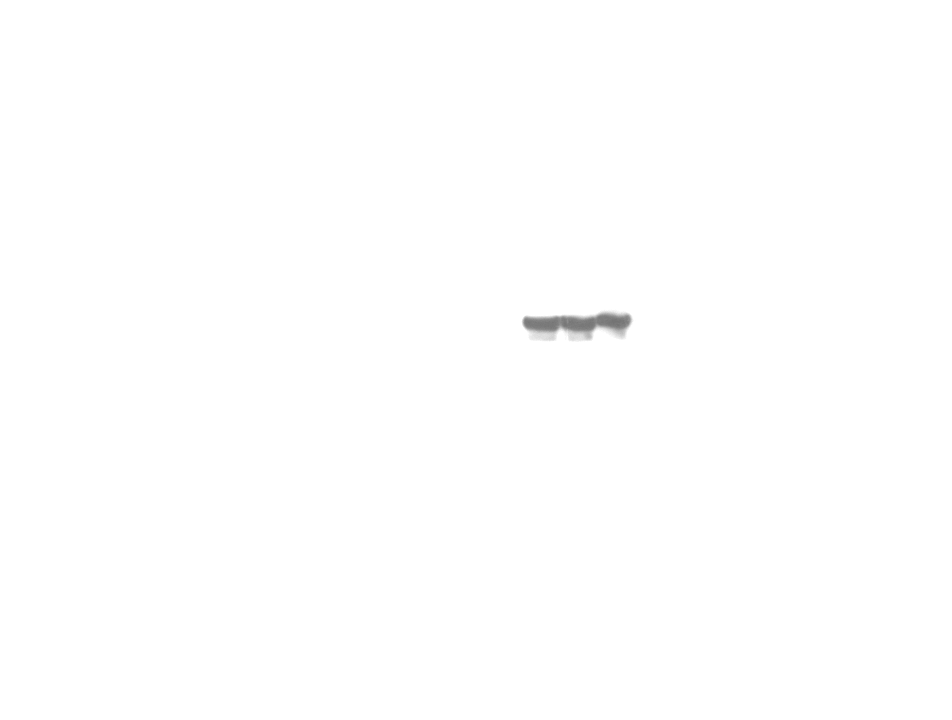

Supplement: Supplementary file 1 [file cancers-14-05976-s001.zip › Original images for blots or gels/Fig5-C/FAK-1.tif]

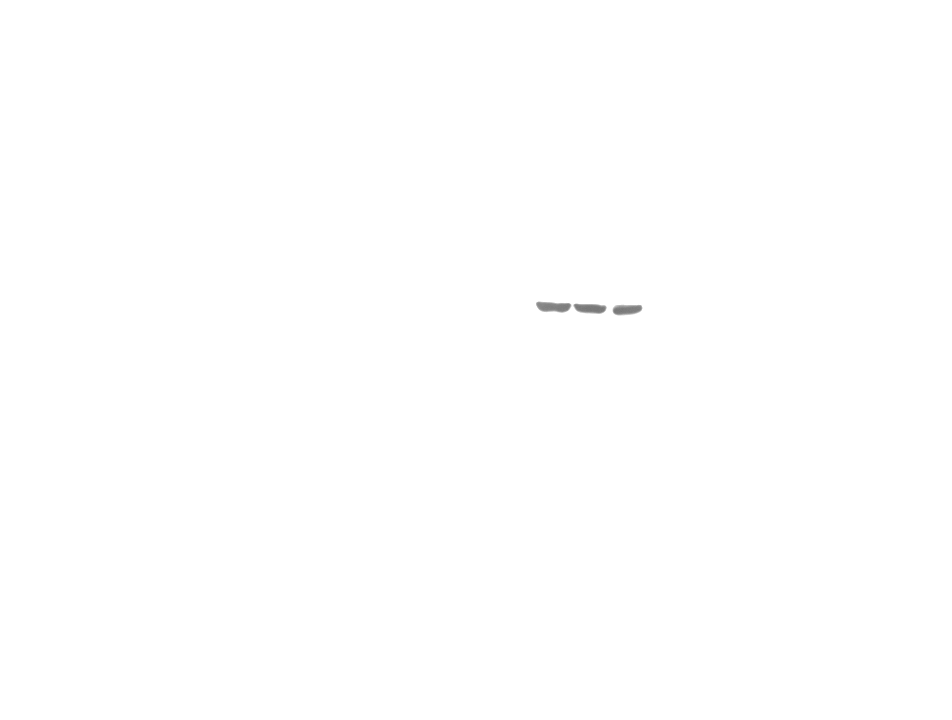

Supplement: Supplementary file 1 [file cancers-14-05976-s001.zip › Original images for blots or gels/Fig5-C/FAK-2.tif]

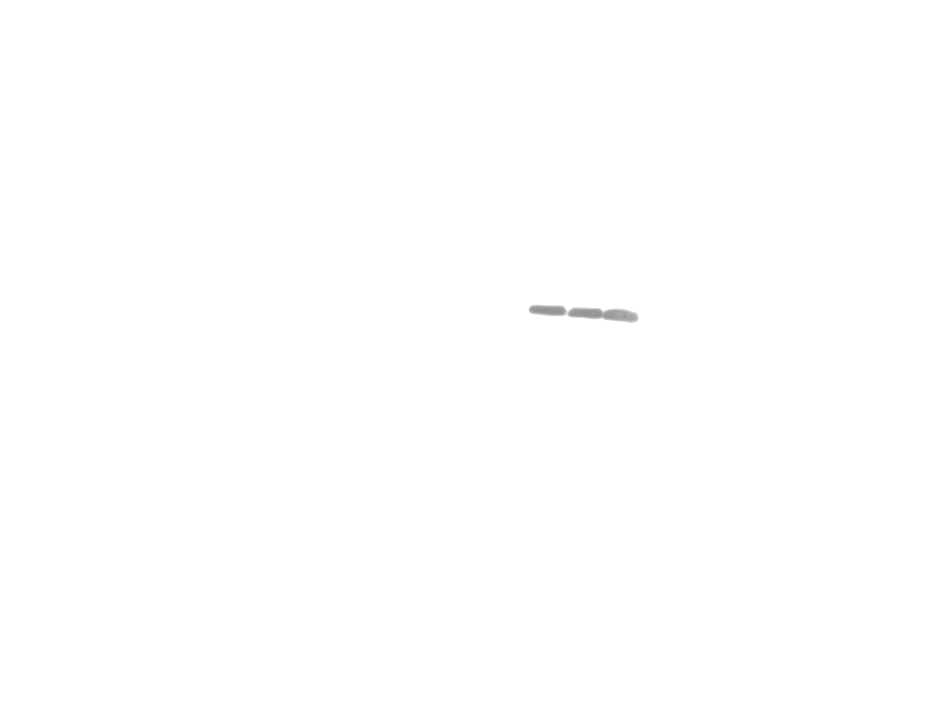

Supplement: Supplementary file 1 [file cancers-14-05976-s001.zip › Original images for blots or gels/Fig5-C/FAK-3.tif]

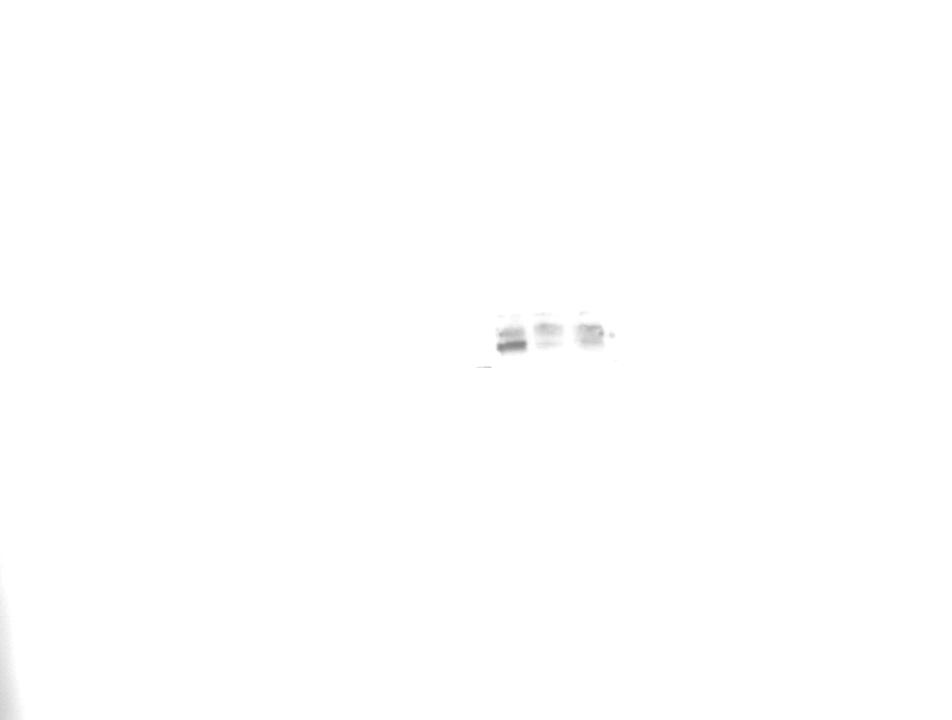

Supplement: Supplementary file 1 [file cancers-14-05976-s001.zip › Original images for blots or gels/Fig5-C/MMP-9-1.tif]

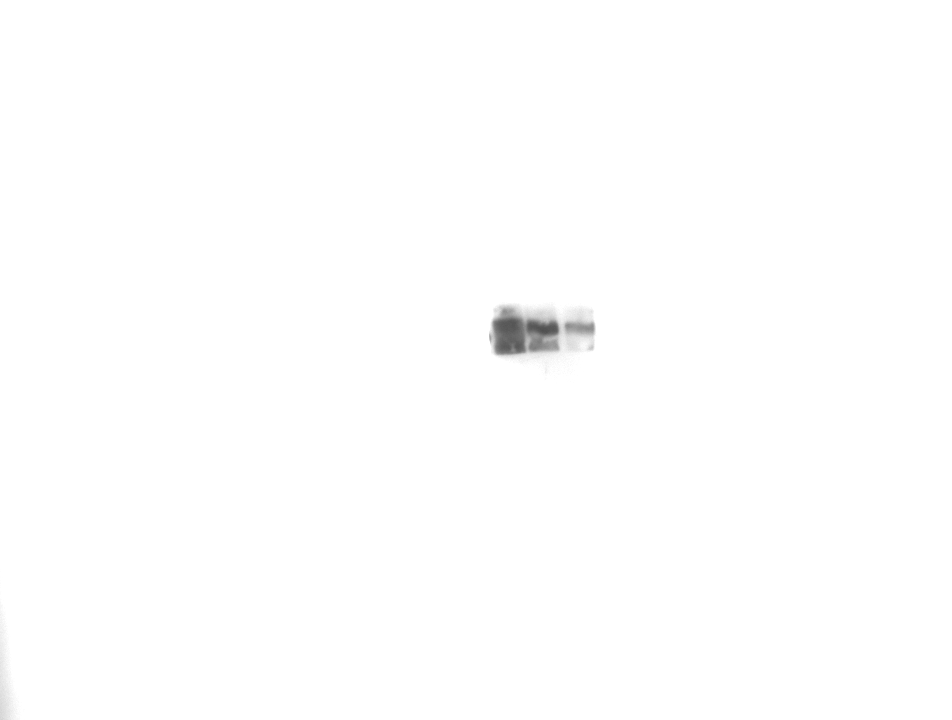

Supplement: Supplementary file 1 [file cancers-14-05976-s001.zip › Original images for blots or gels/Fig5-C/MMP-9-2.tif]

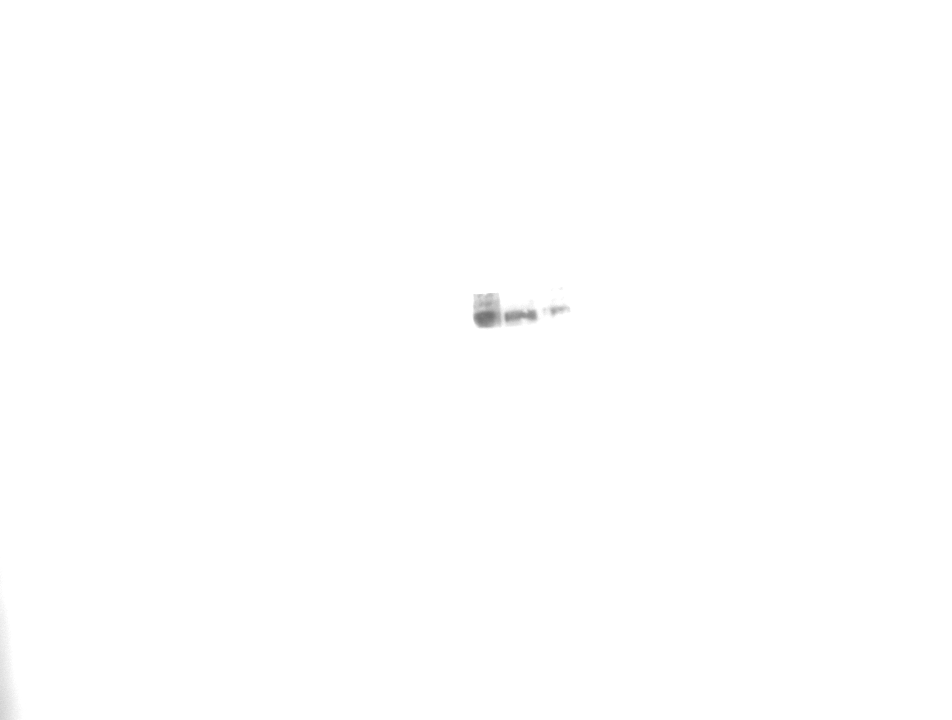

Supplement: Supplementary file 1 [file cancers-14-05976-s001.zip › Original images for blots or gels/Fig5-C/MMP-9-3.tif]

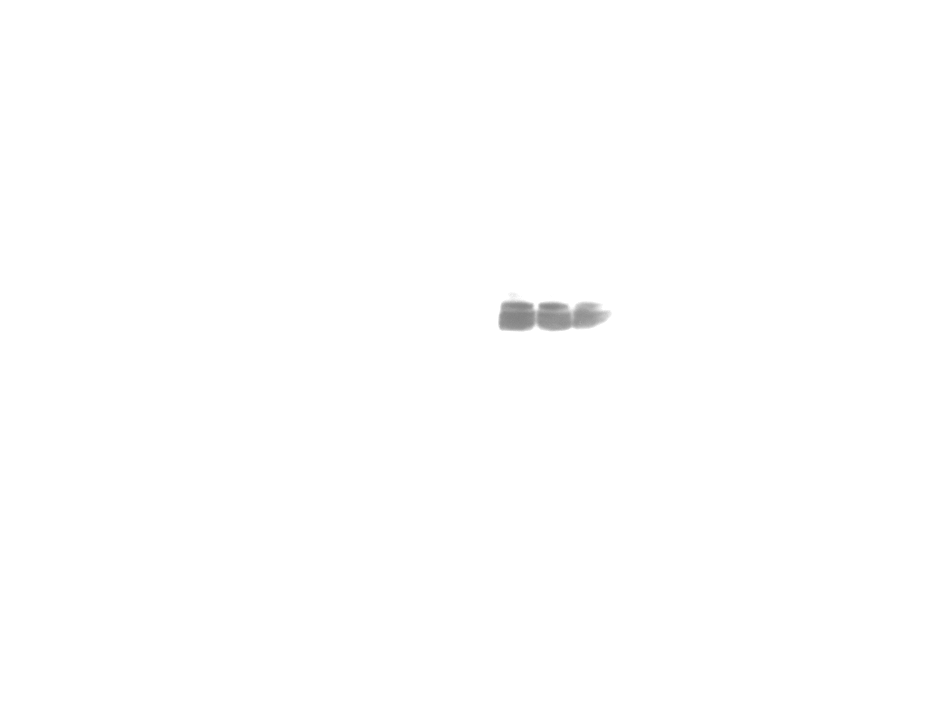

Supplement: Supplementary file 1 [file cancers-14-05976-s001.zip › Original images for blots or gels/Fig5-C/p-AKT-1.tif]

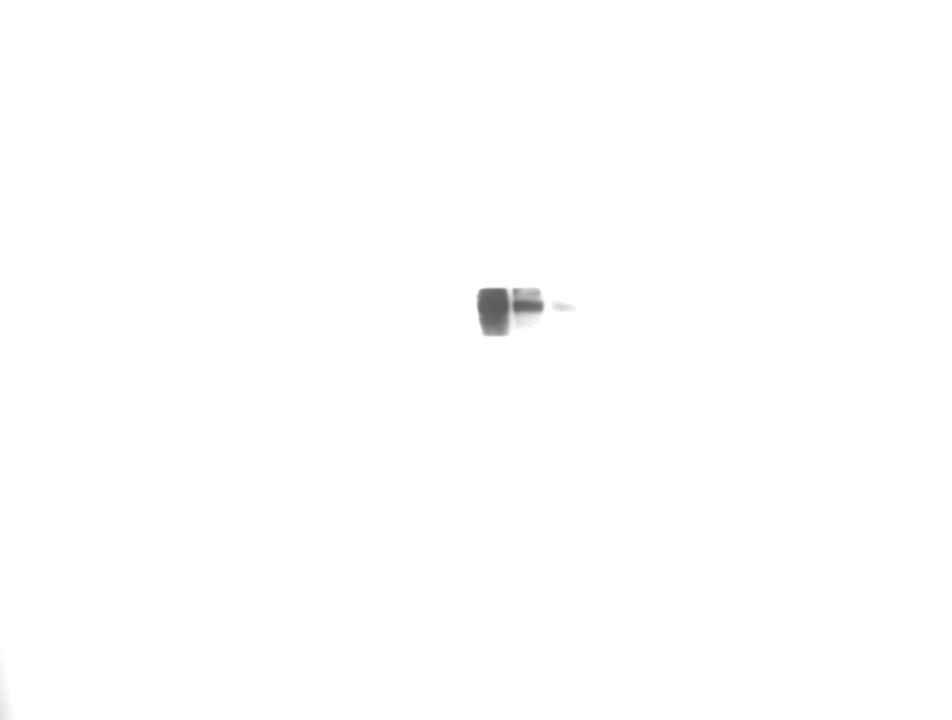

Supplement: Supplementary file 1 [file cancers-14-05976-s001.zip › Original images for blots or gels/Fig5-C/p-AKT-2.tif]

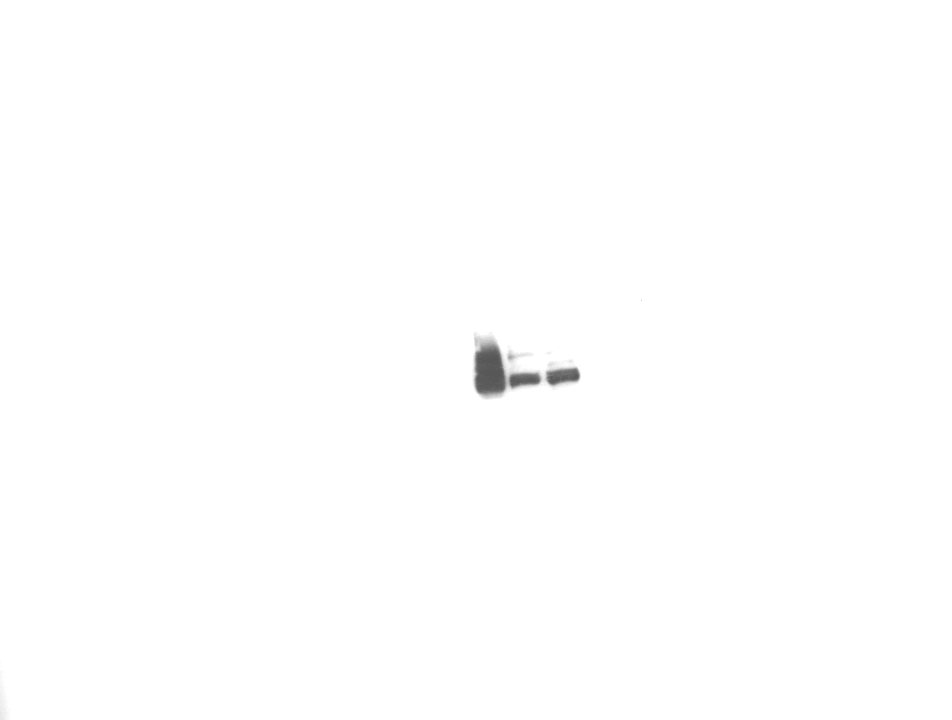

Supplement: Supplementary file 1 [file cancers-14-05976-s001.zip › Original images for blots or gels/Fig5-C/p-AKT-3.tif]

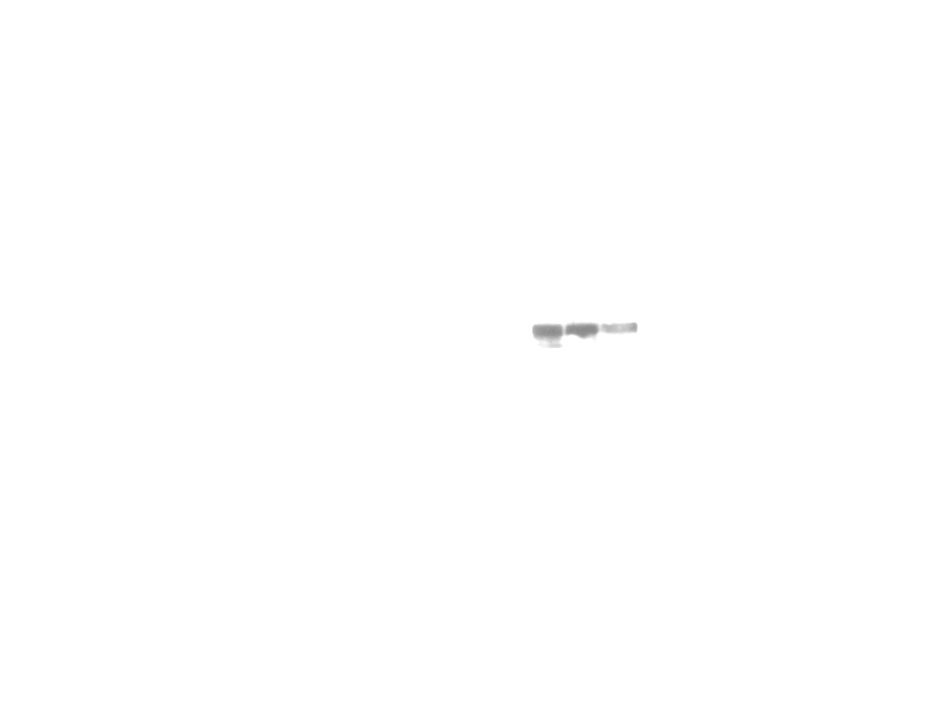

Supplement: Supplementary file 1 [file cancers-14-05976-s001.zip › Original images for blots or gels/Fig5-C/p-FAK-1.tif]

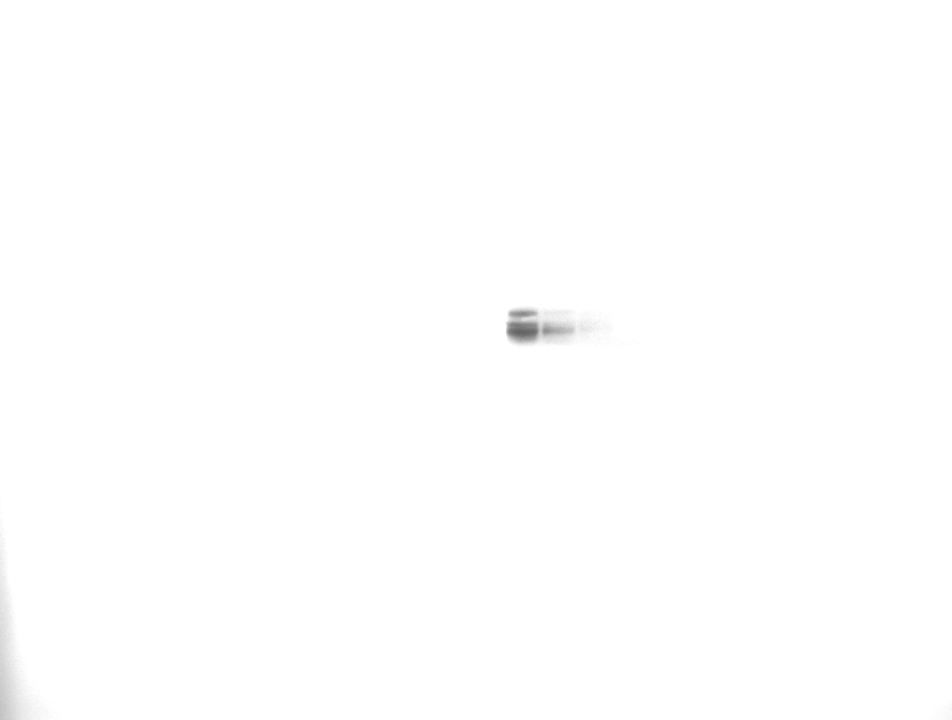

Supplement: Supplementary file 1 [file cancers-14-05976-s001.zip › Original images for blots or gels/Fig5-C/p-FAK-2.tif]

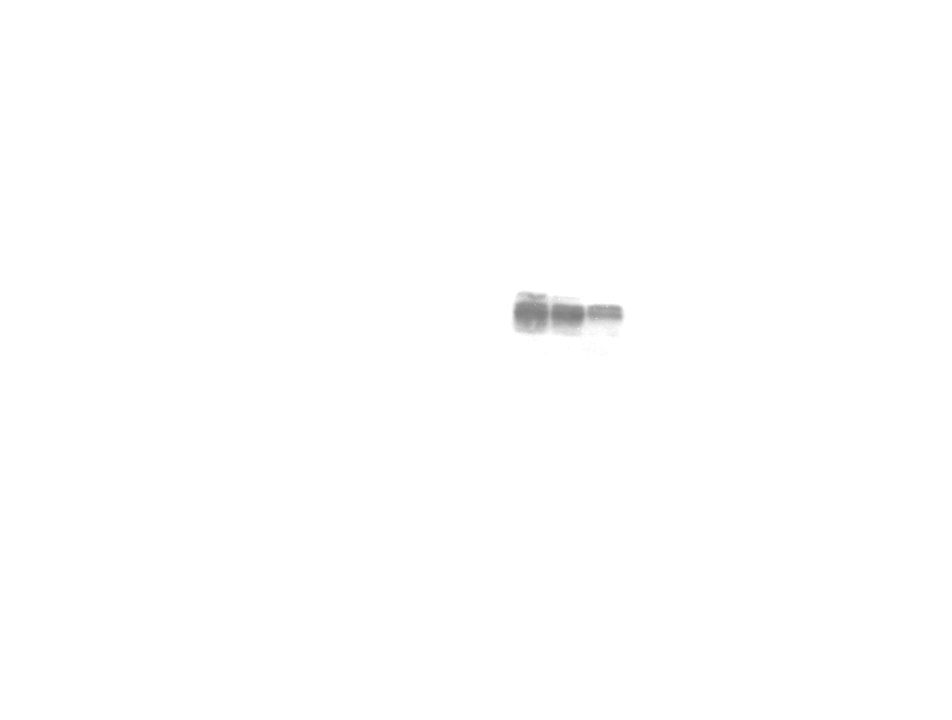

Supplement: Supplementary file 1 [file cancers-14-05976-s001.zip › Original images for blots or gels/Fig5-C/p-FAK-3.tif]

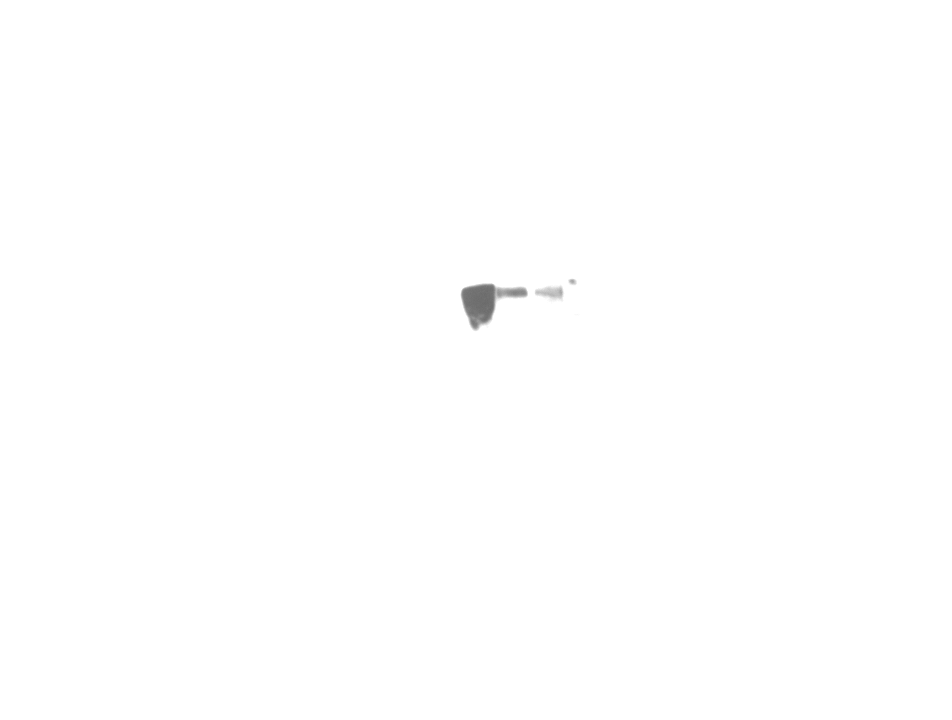

Supplement: Supplementary file 1 [file cancers-14-05976-s001.zip › Original images for blots or gels/Fig5-C/p-PI3K-1.tif]

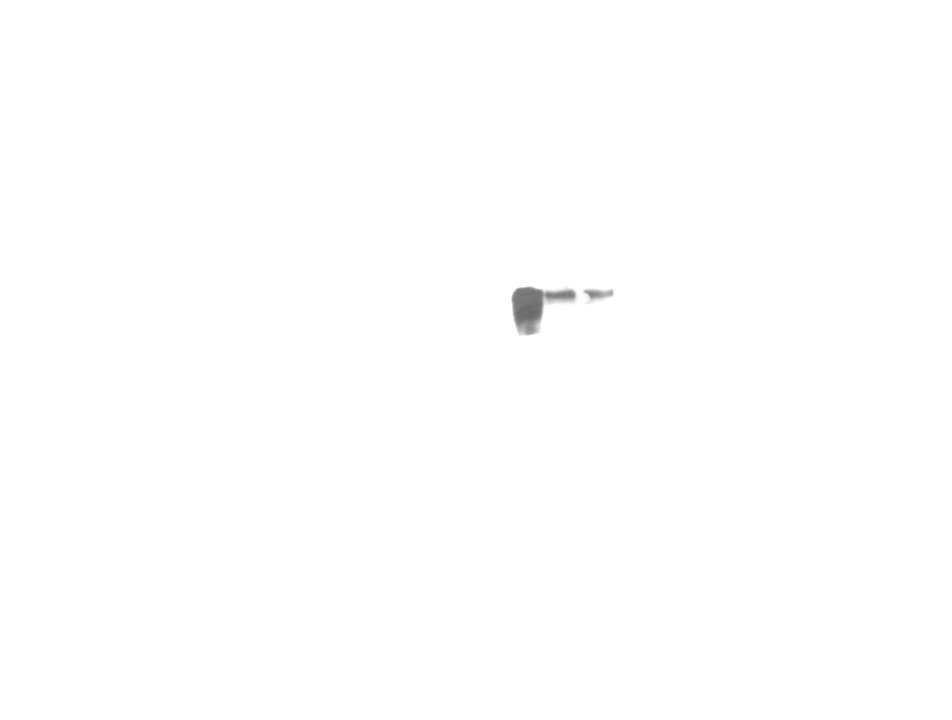

Supplement: Supplementary file 1 [file cancers-14-05976-s001.zip › Original images for blots or gels/Fig5-C/p-PI3K-2.tif]

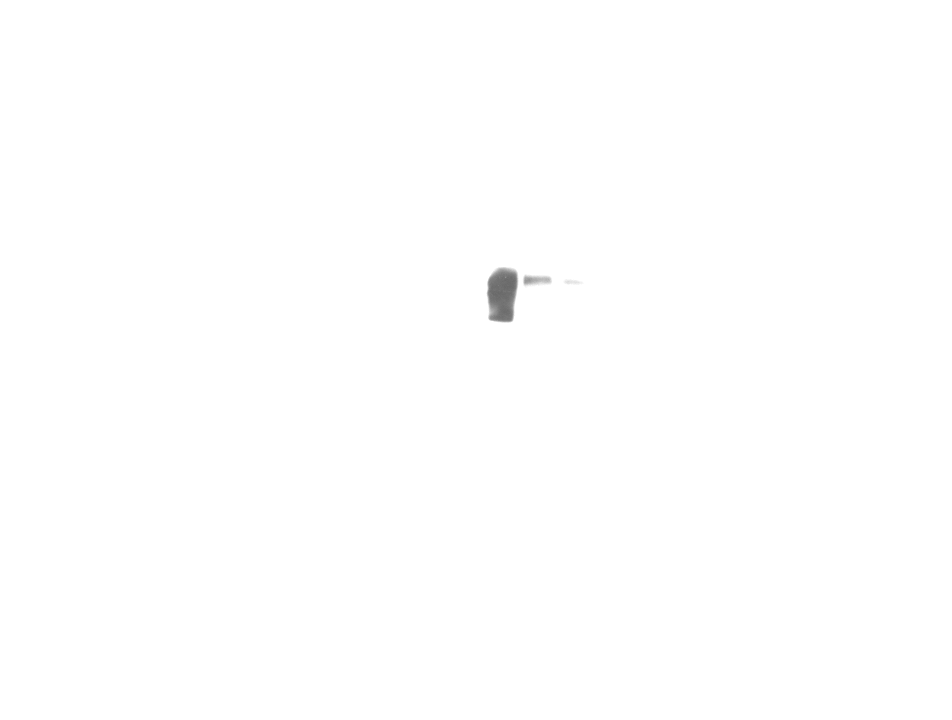

Supplement: Supplementary file 1 [file cancers-14-05976-s001.zip › Original images for blots or gels/Fig5-C/p-PI3K-3.tif]

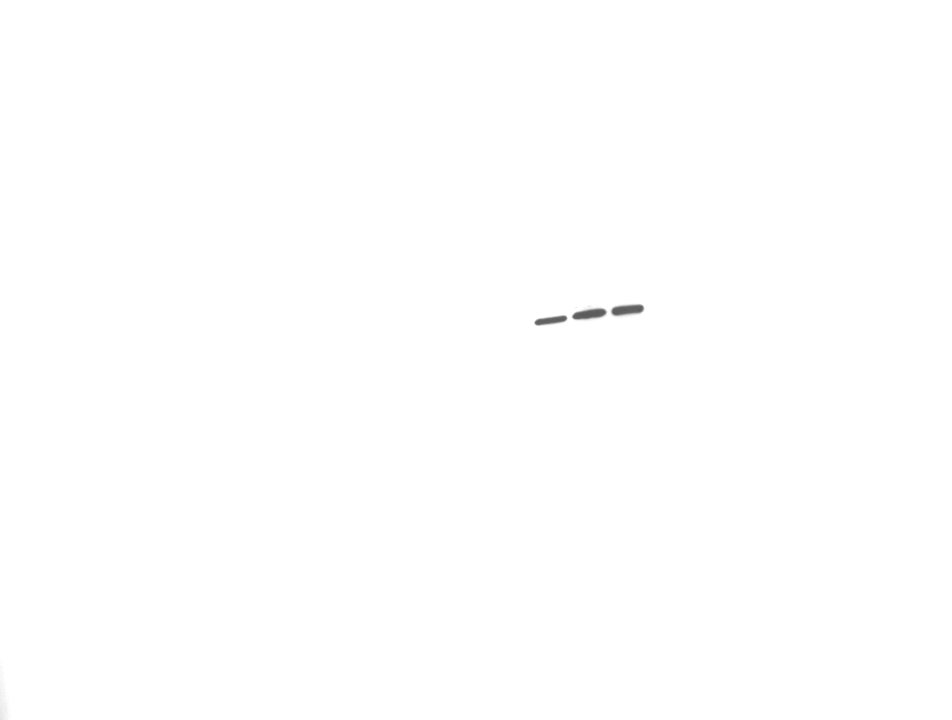

Supplement: Supplementary file 1 [file cancers-14-05976-s001.zip › Original images for blots or gels/Fig5-C/PI3K-1.tif]

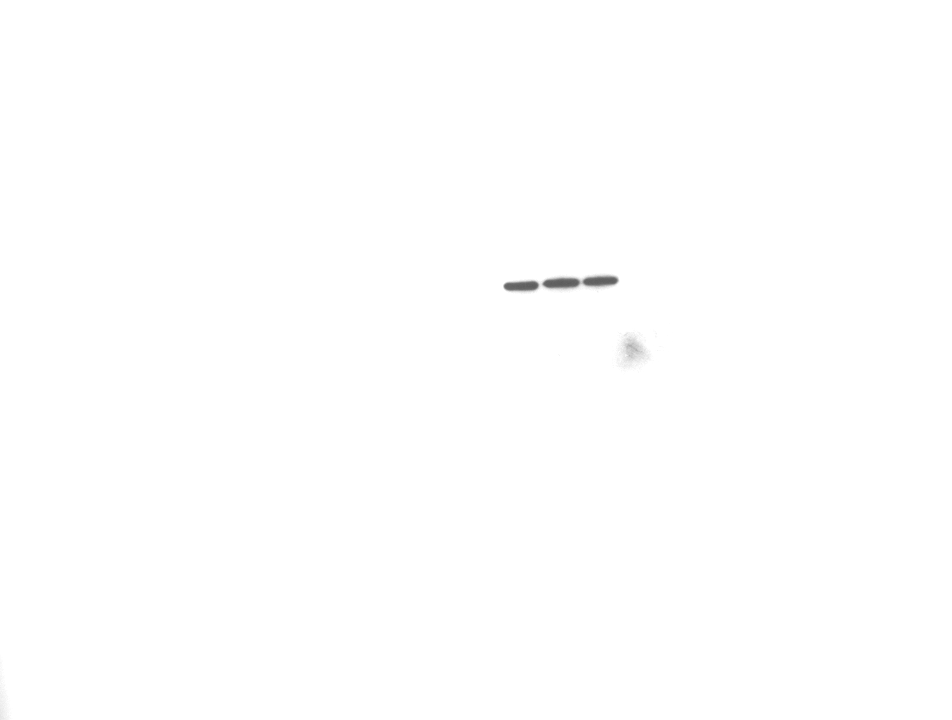

Supplement: Supplementary file 1 [file cancers-14-05976-s001.zip › Original images for blots or gels/Fig5-C/PI3K-2.tif]

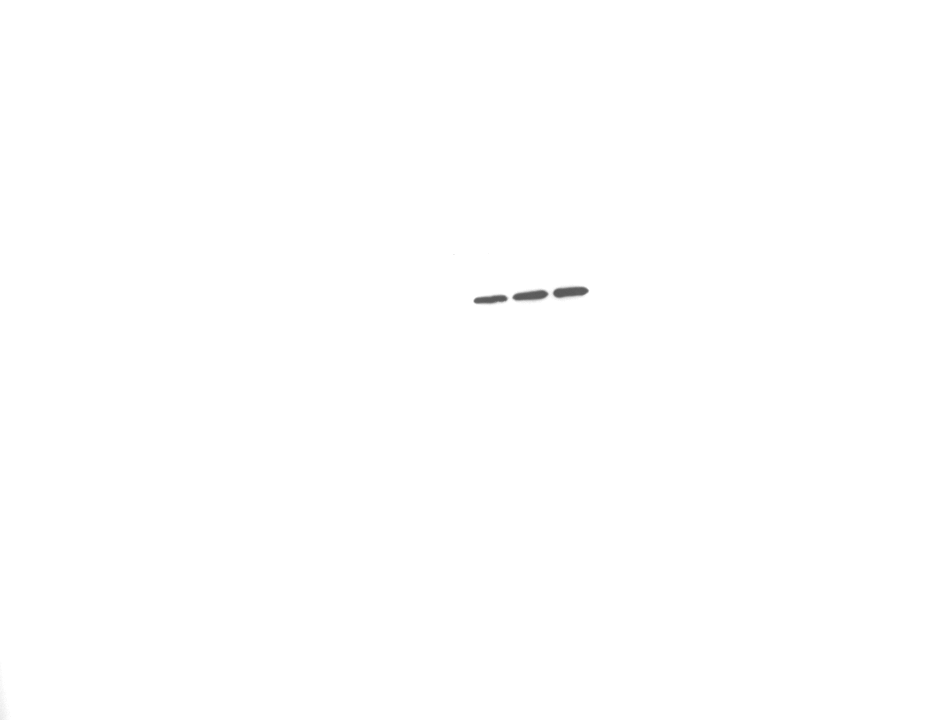

Supplement: Supplementary file 1 [file cancers-14-05976-s001.zip › Original images for blots or gels/Fig5-C/PI3K-3.tif]

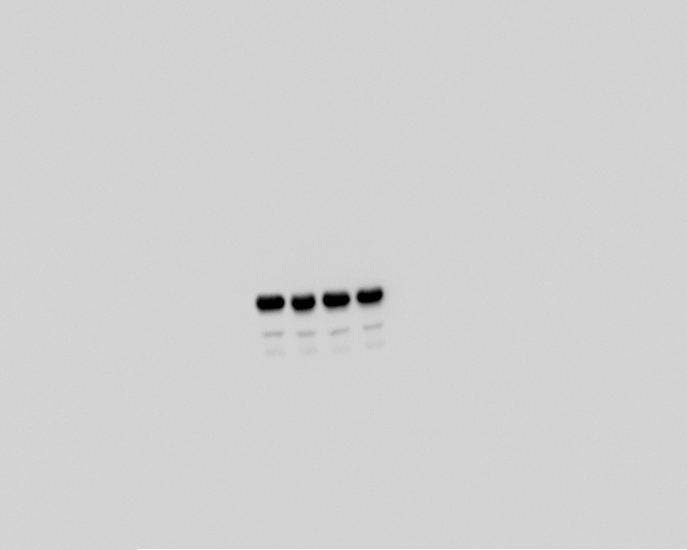

Supplement: Supplementary file 1 [file cancers-14-05976-s001.zip › Original images for blots or gels/Fig5-D/GAPDH-1.tif]

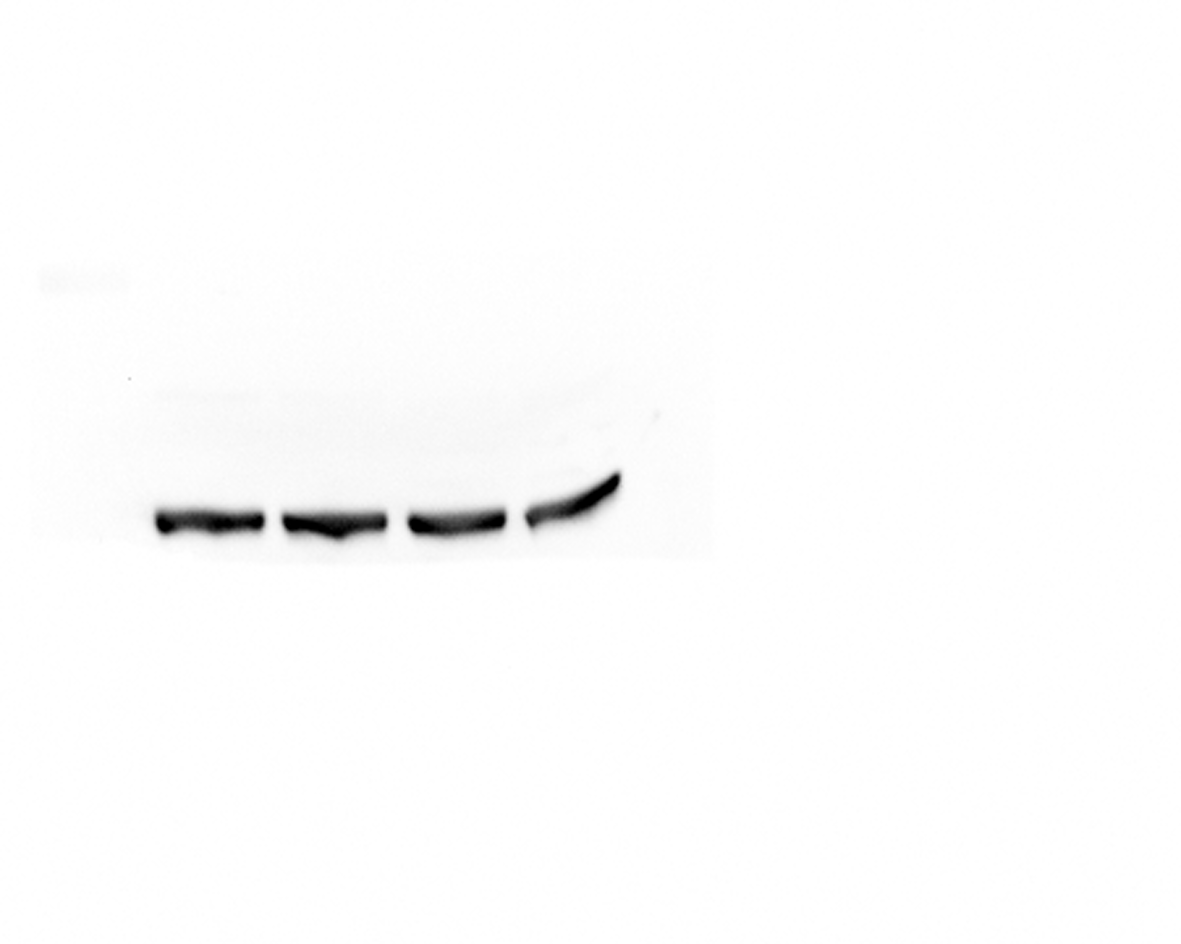

Supplement: Supplementary file 1 [file cancers-14-05976-s001.zip › Original images for blots or gels/Fig5-D/GAPDH-2.tif]

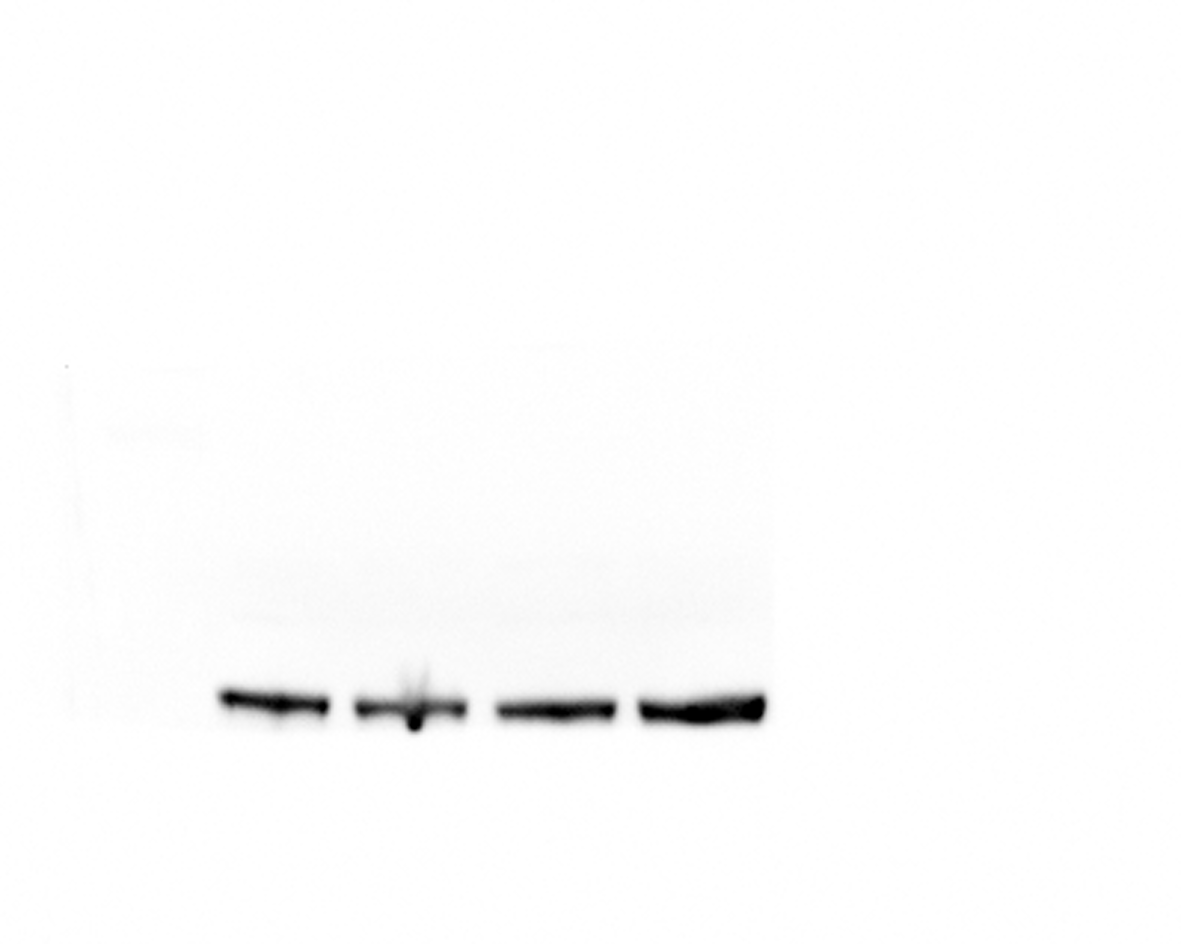

Supplement: Supplementary file 1 [file cancers-14-05976-s001.zip › Original images for blots or gels/Fig5-D/GAPDH-3.tif]

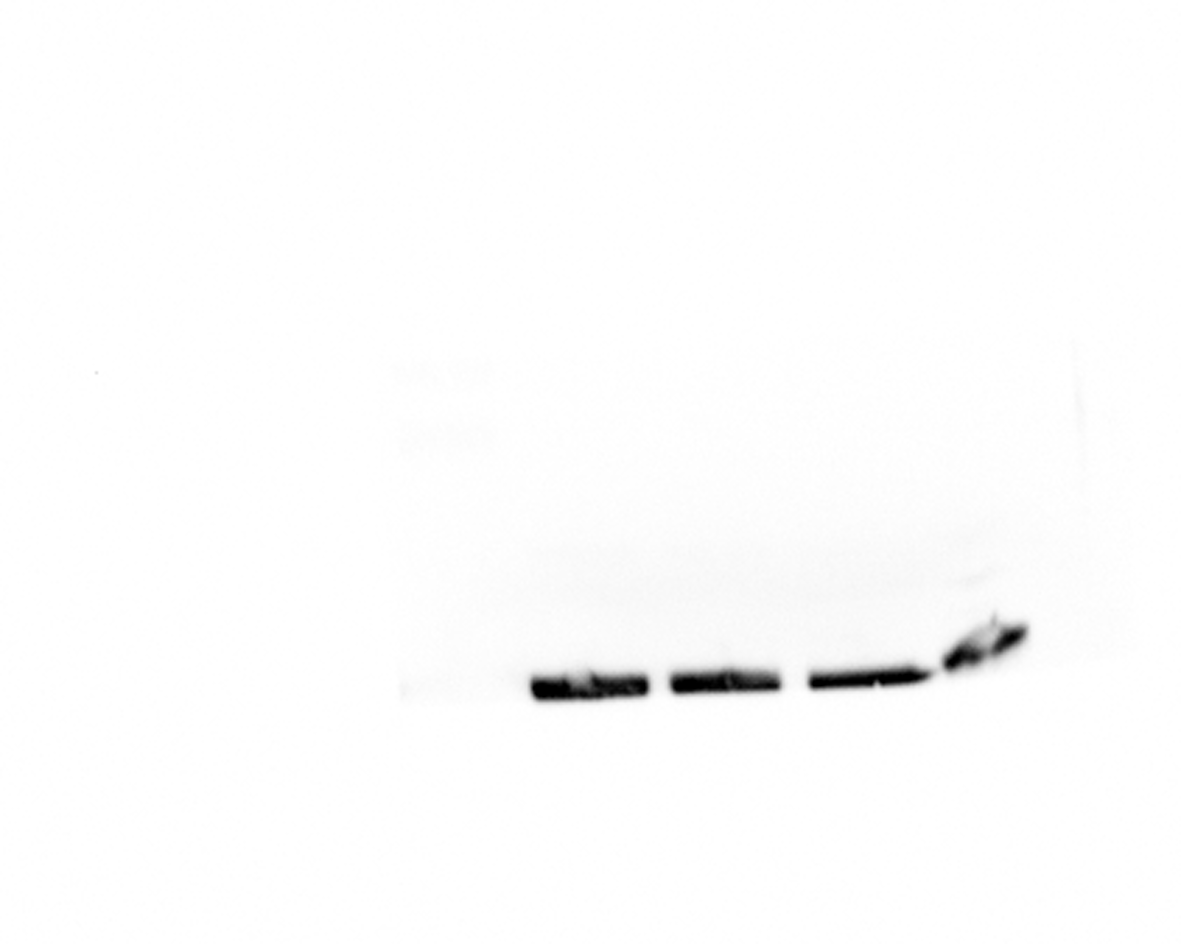

Supplement: Supplementary file 1 [file cancers-14-05976-s001.zip › Original images for blots or gels/Fig5-D/GAPDH-4.tif]

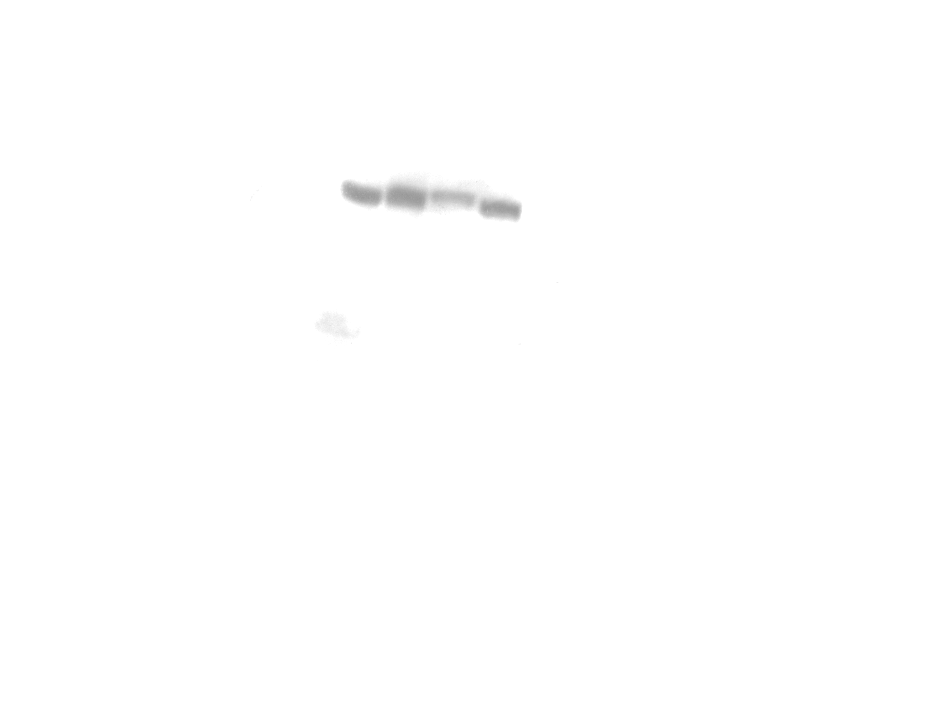

Supplement: Supplementary file 1 [file cancers-14-05976-s001.zip › Original images for blots or gels/Fig5-D/MMP-9-1.tif]

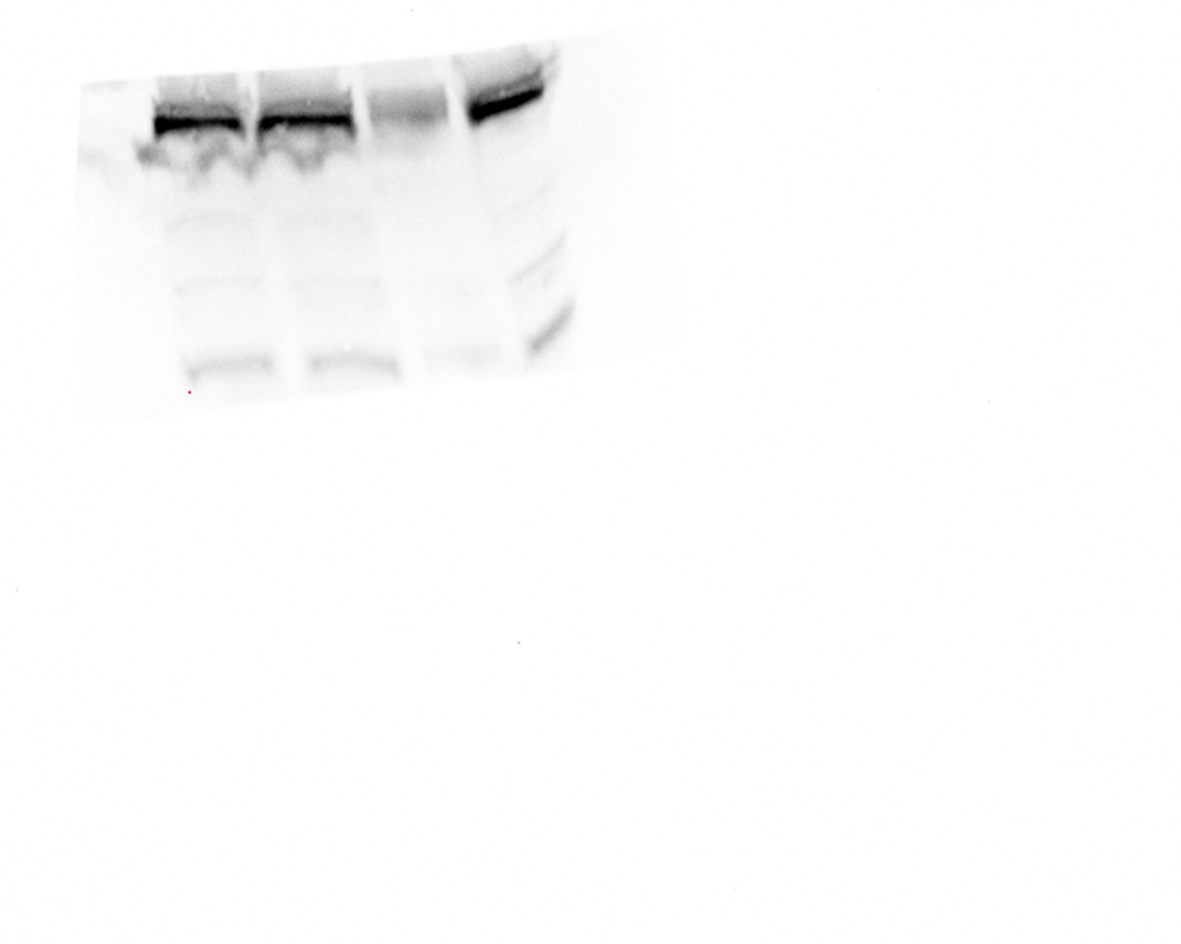

Supplement: Supplementary file 1 [file cancers-14-05976-s001.zip › Original images for blots or gels/Fig5-D/MMP-9-2.tif]

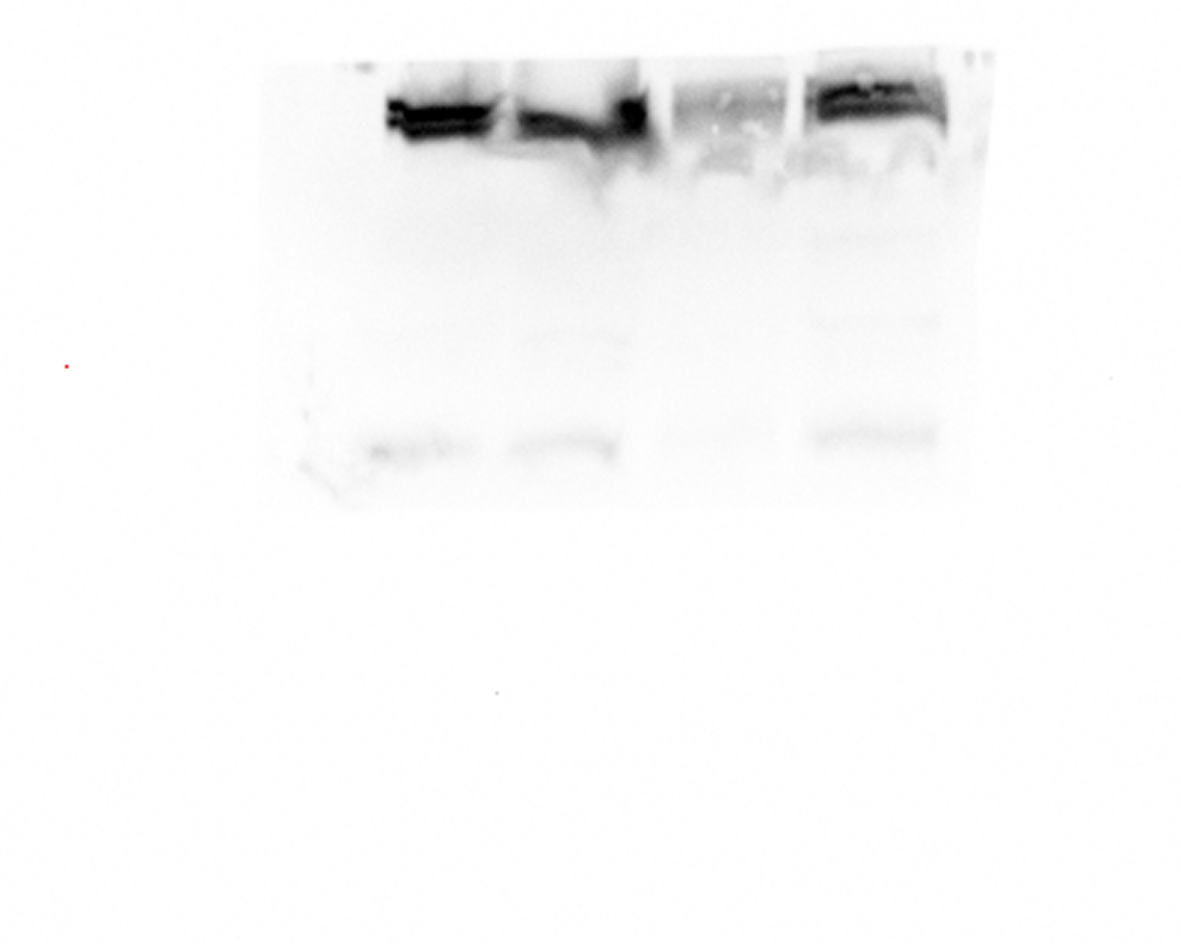

Supplement: Supplementary file 1 [file cancers-14-05976-s001.zip › Original images for blots or gels/Fig5-D/MMP-9-3.tif]

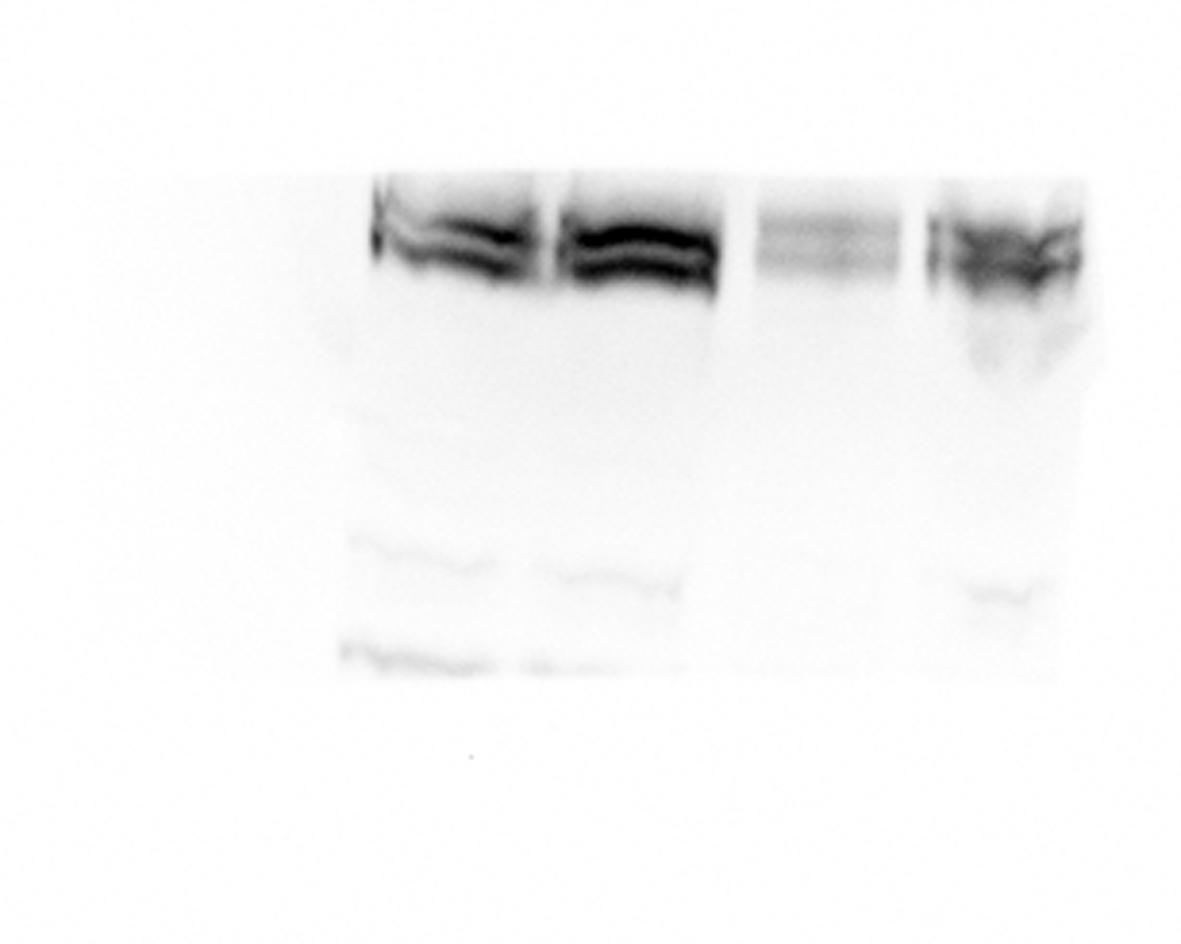

Supplement: Supplementary file 1 [file cancers-14-05976-s001.zip › Original images for blots or gels/Fig5-D/MMP-9-4.tif]

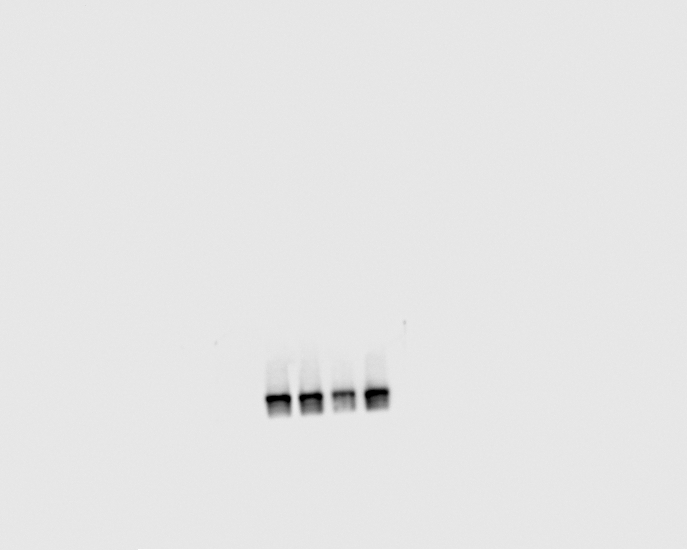

Supplement: Supplementary file 1 [file cancers-14-05976-s001.zip › Original images for blots or gels/Fig5-D/p-AKT-1.tif]

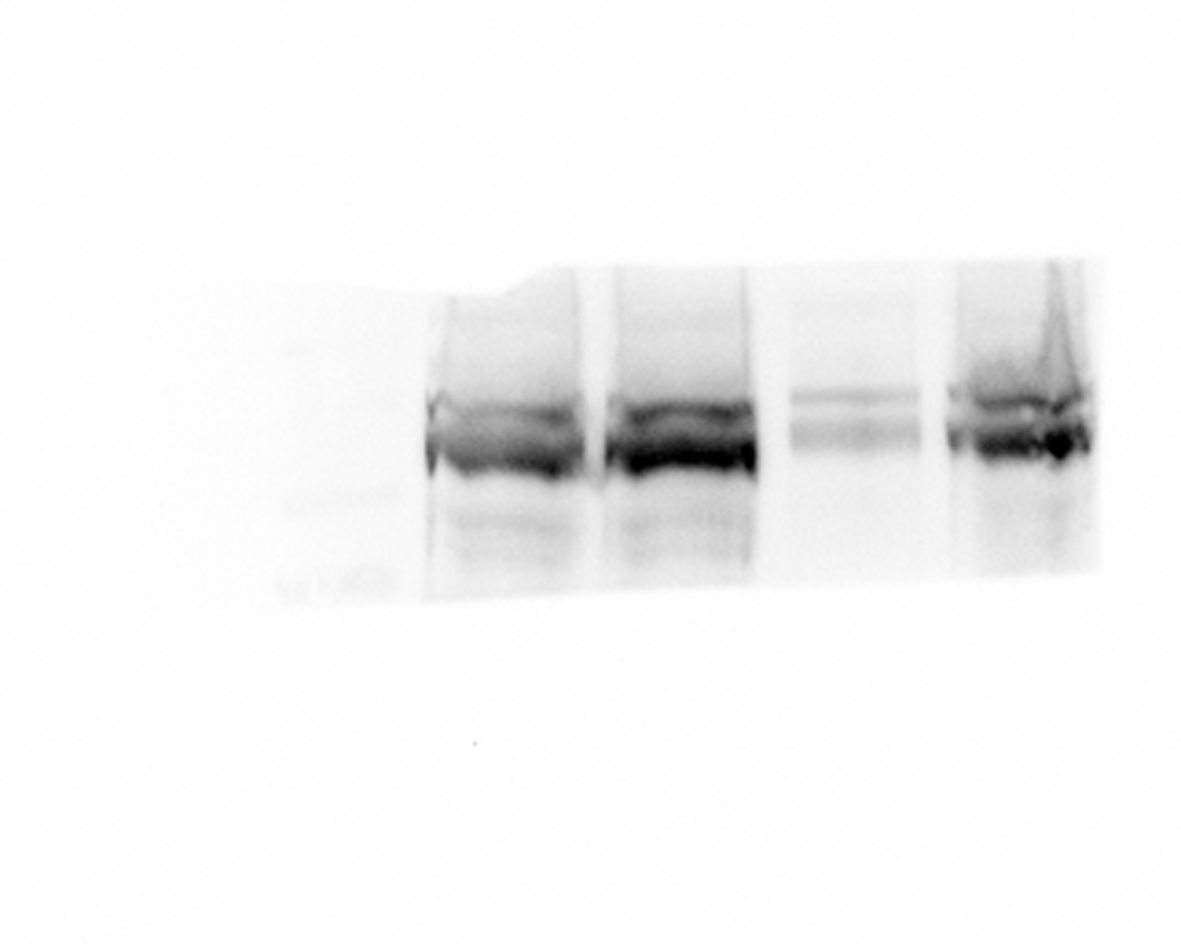

Supplement: Supplementary file 1 [file cancers-14-05976-s001.zip › Original images for blots or gels/Fig5-D/p-AKT-2.tif]

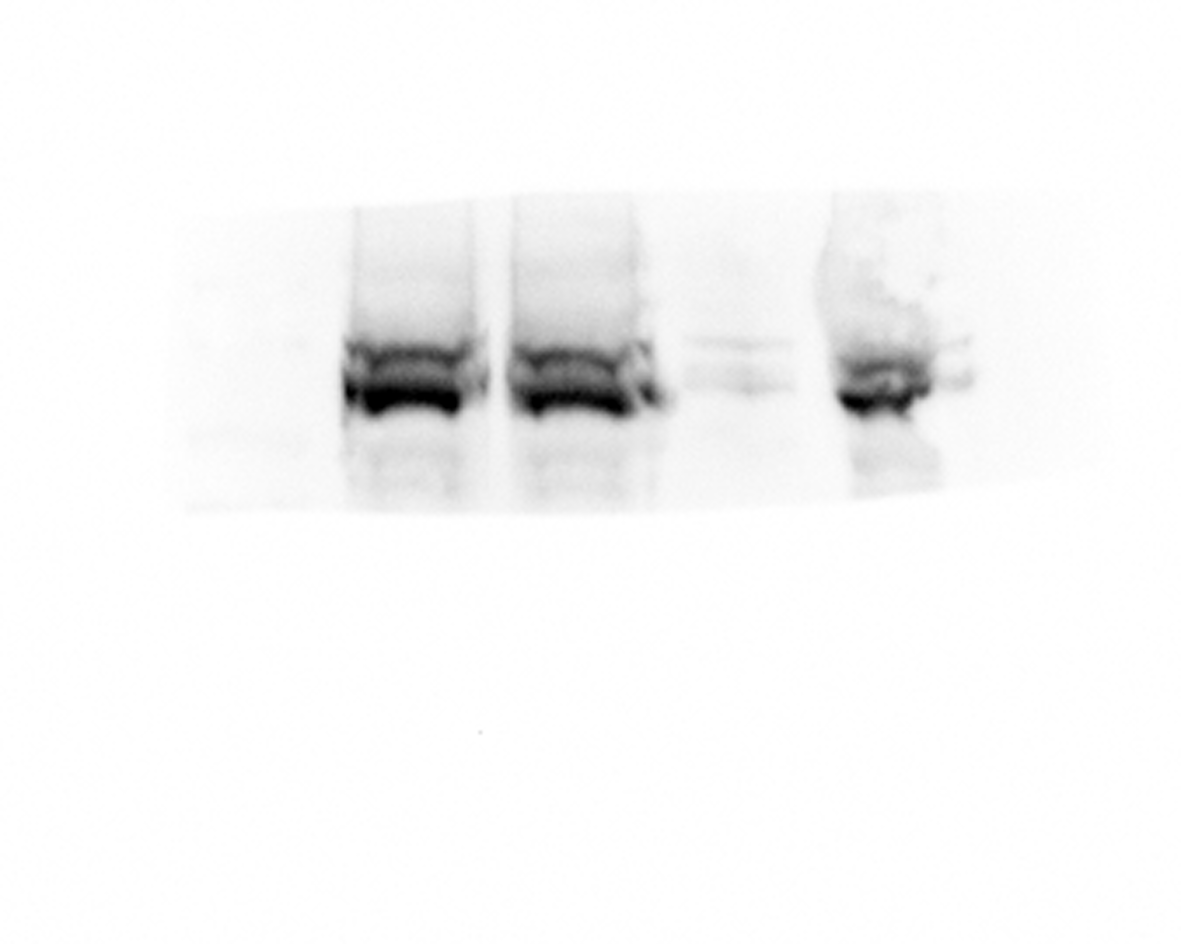

Supplement: Supplementary file 1 [file cancers-14-05976-s001.zip › Original images for blots or gels/Fig5-D/p-AKT-3.tif]

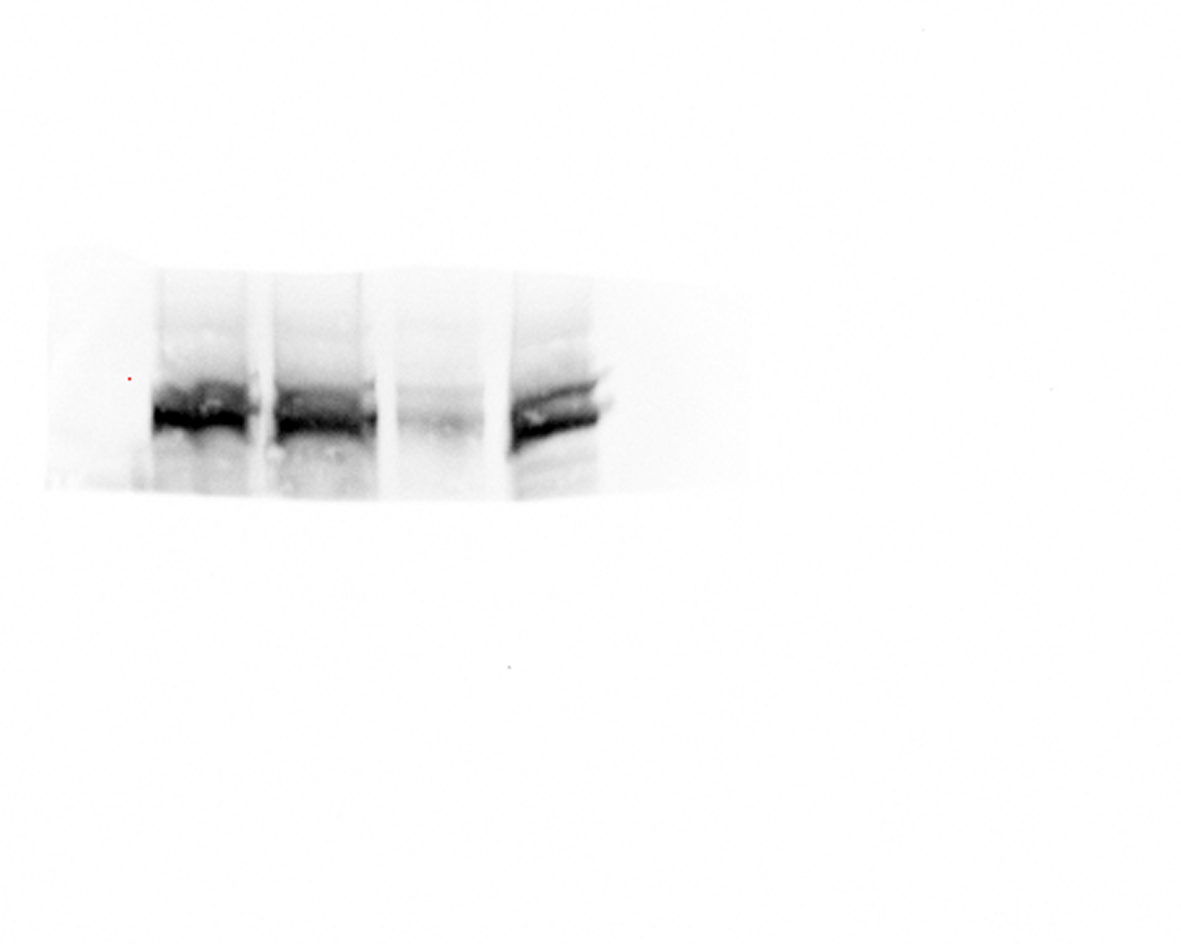

Supplement: Supplementary file 1 [file cancers-14-05976-s001.zip › Original images for blots or gels/Fig5-D/p-AKT-4.tif]

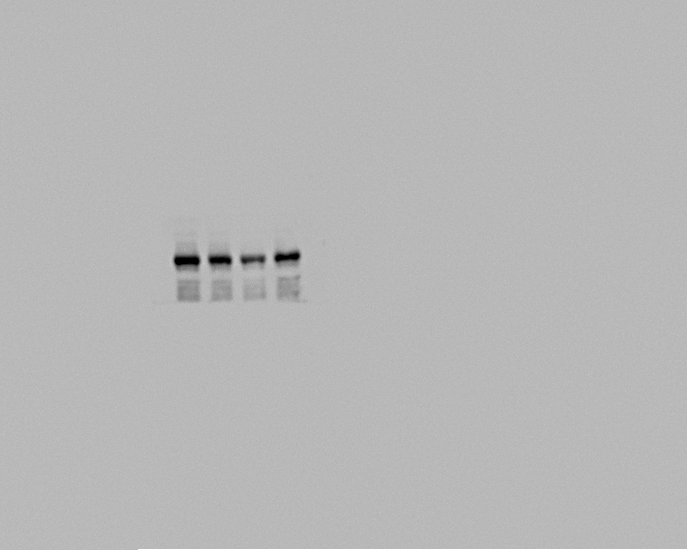

Supplement: Supplementary file 1 [file cancers-14-05976-s001.zip › Original images for blots or gels/Fig5-D/p-FAK(Y397)-1.tif]

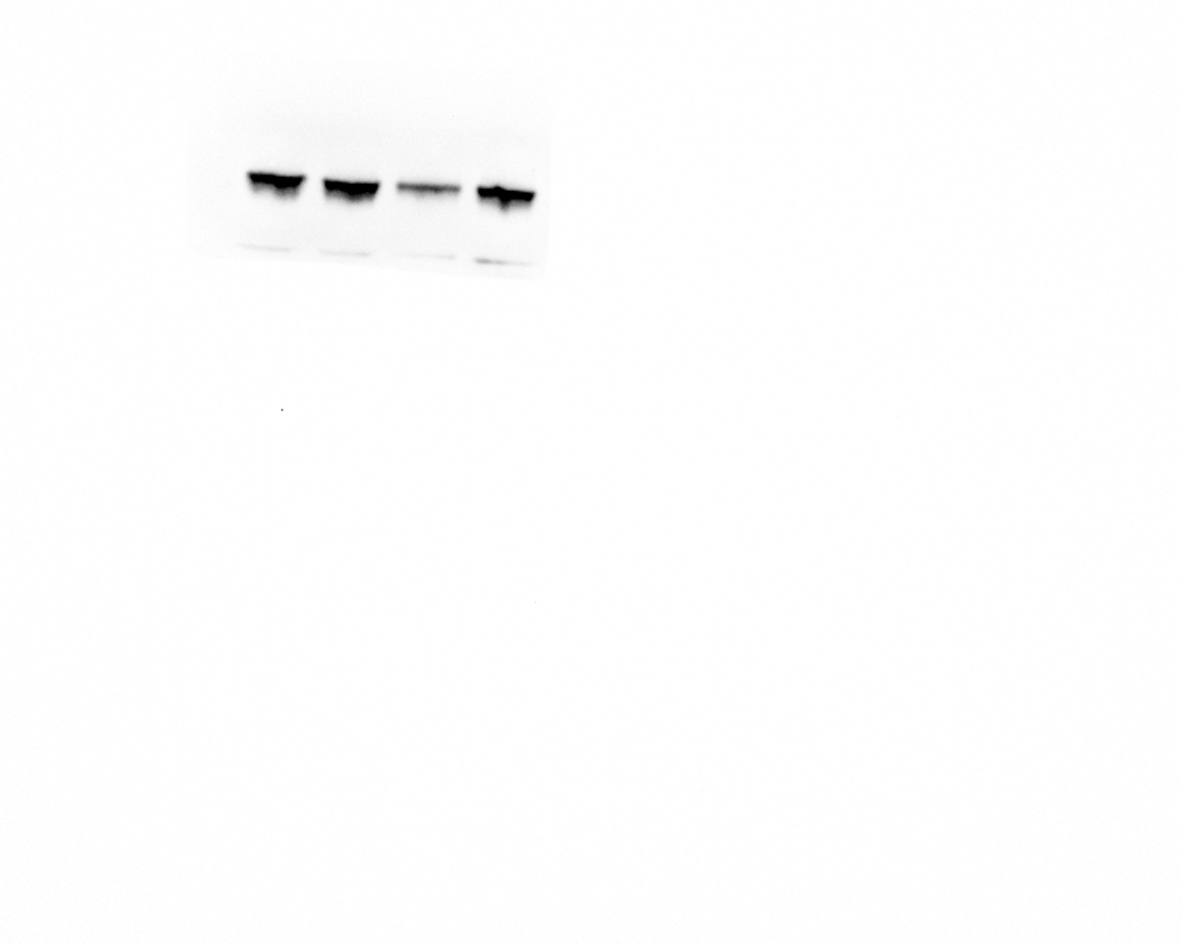

Supplement: Supplementary file 1 [file cancers-14-05976-s001.zip › Original images for blots or gels/Fig5-D/p-FAK(Y397)-2.tif]

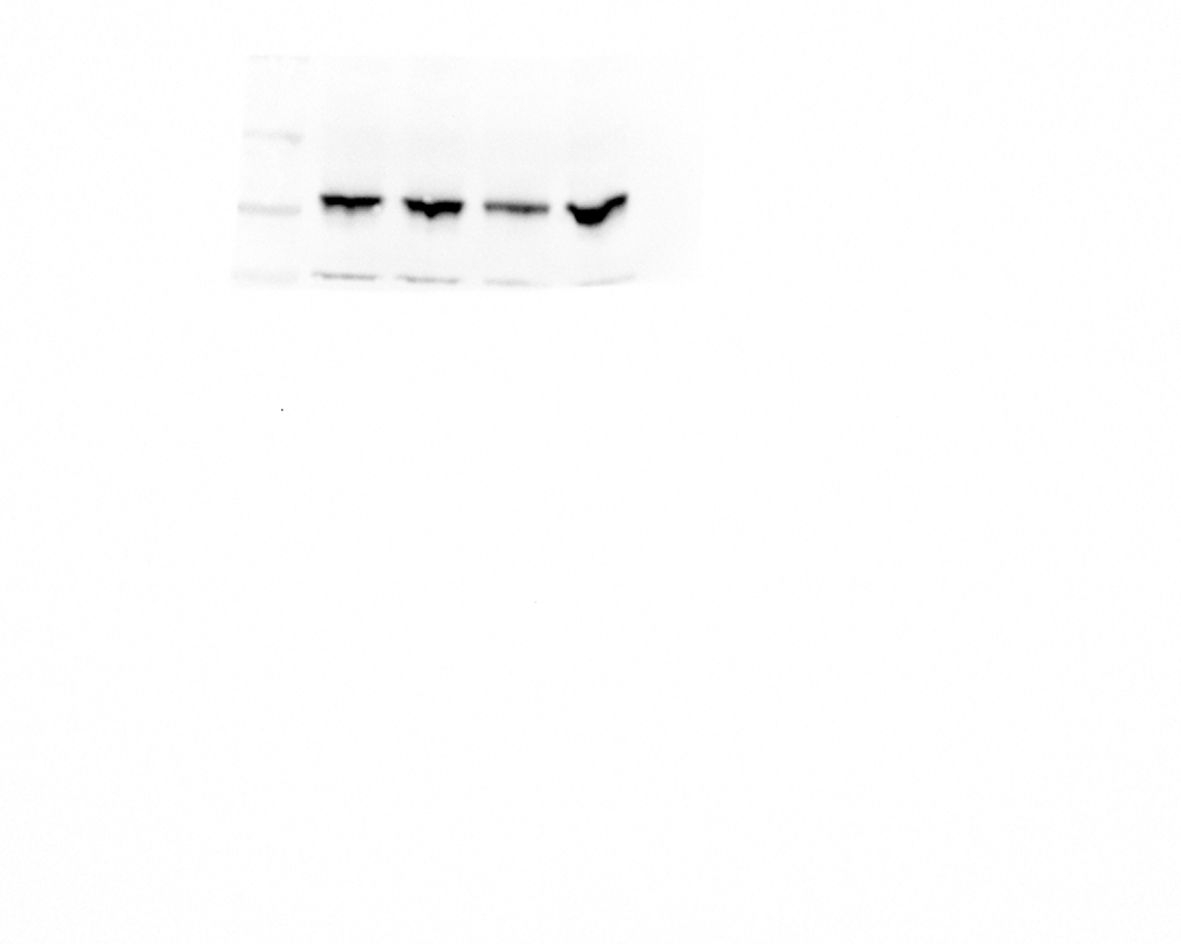

Supplement: Supplementary file 1 [file cancers-14-05976-s001.zip › Original images for blots or gels/Fig5-D/p-FAK(Y397)-3.tif]

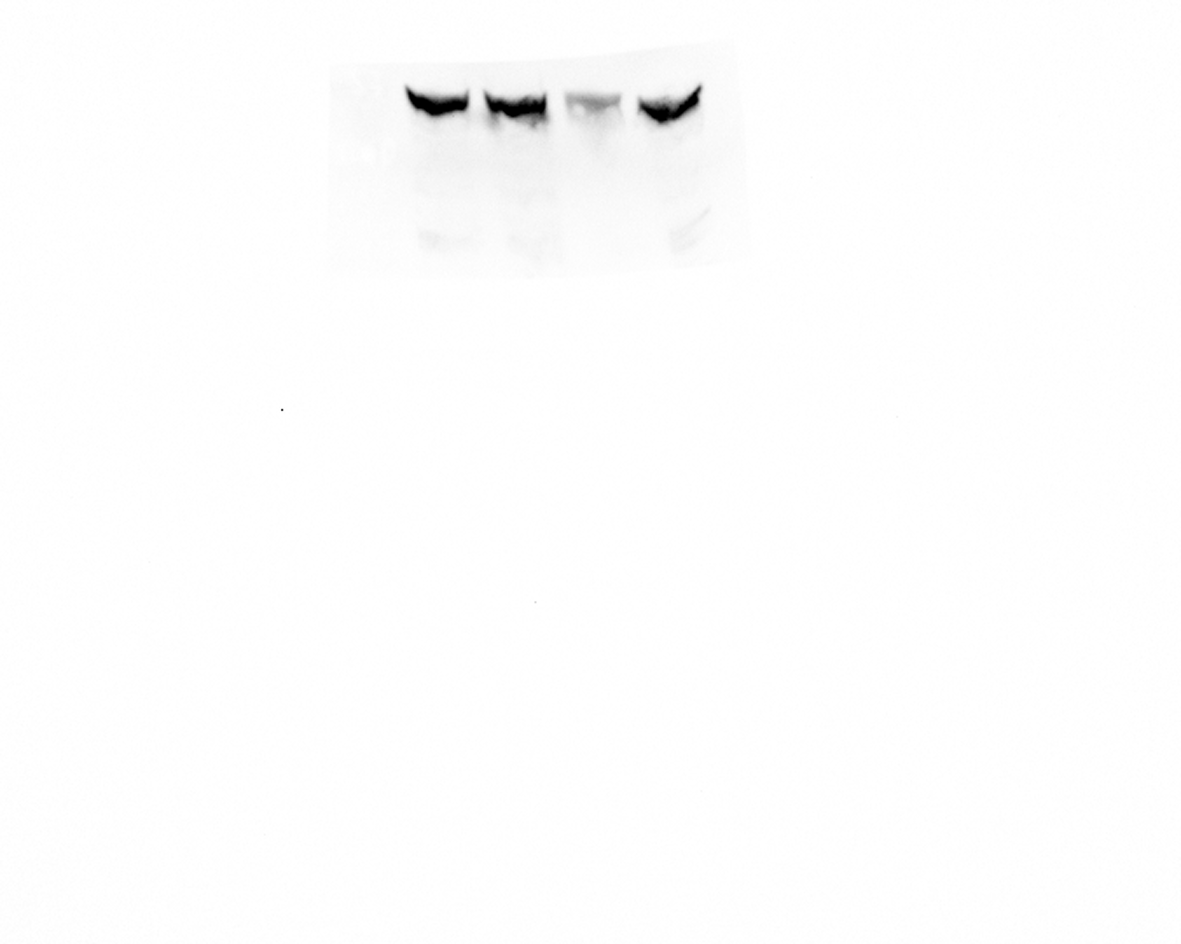

Supplement: Supplementary file 1 [file cancers-14-05976-s001.zip › Original images for blots or gels/Fig5-D/p-FAK(Y397)-4.tif]

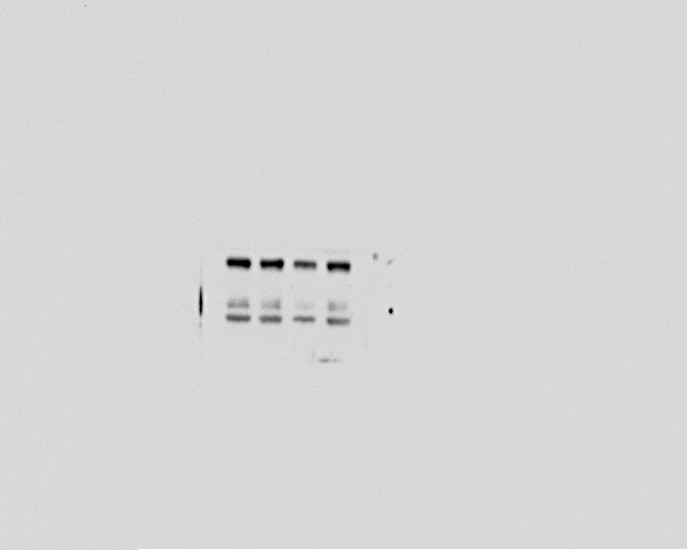

Supplement: Supplementary file 1 [file cancers-14-05976-s001.zip › Original images for blots or gels/Fig5-D/p-PI3K(607)-1.tif]

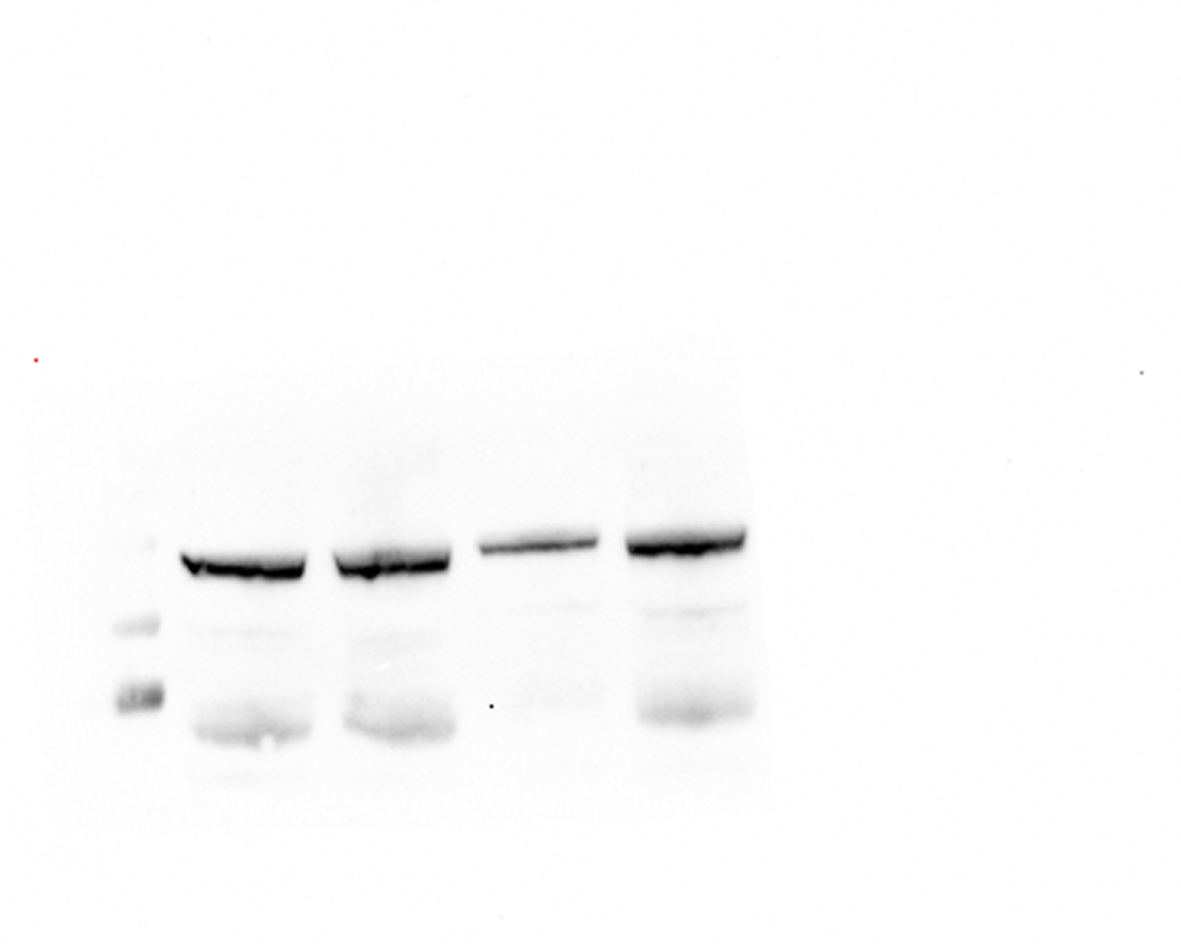

Supplement: Supplementary file 1 [file cancers-14-05976-s001.zip › Original images for blots or gels/Fig5-D/p-PI3K(607)-2.tif]

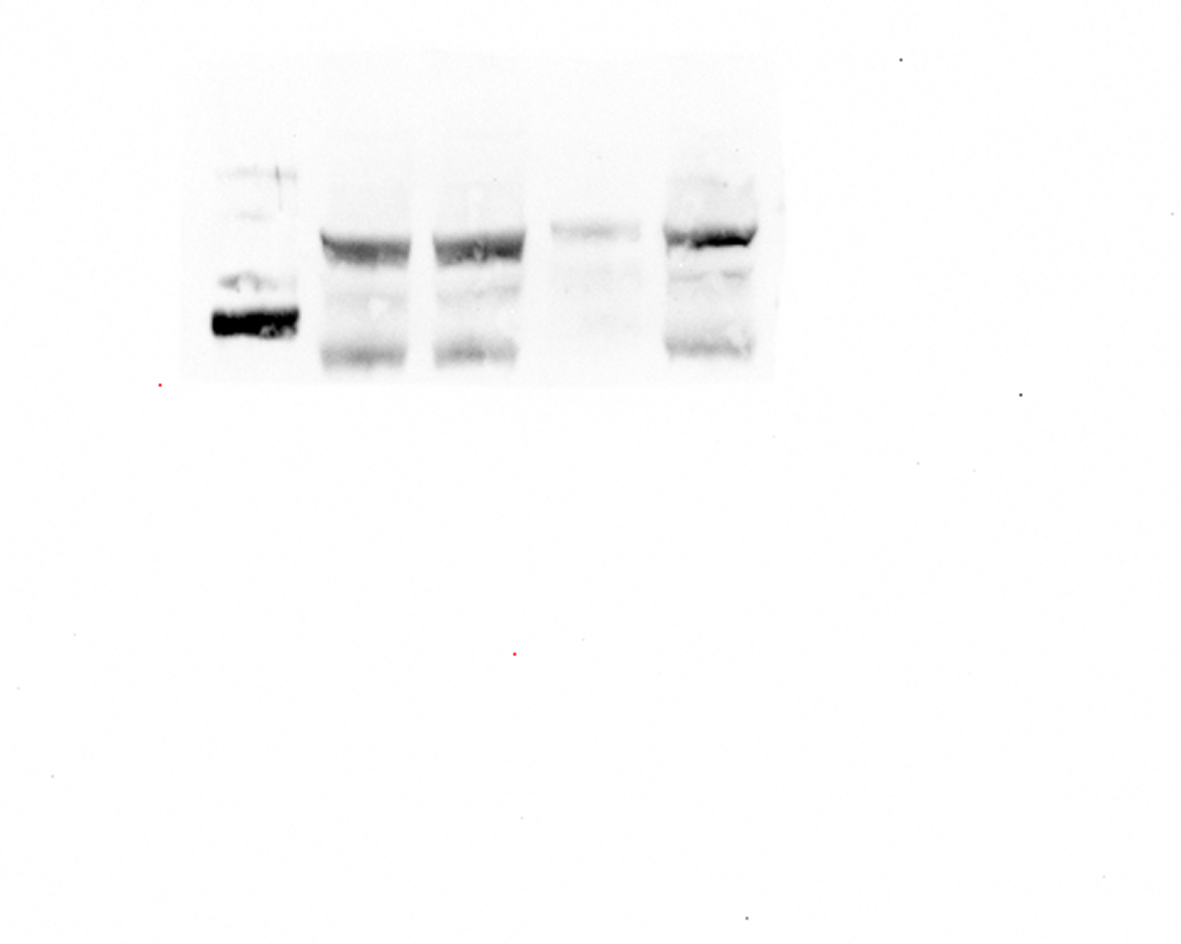

Supplement: Supplementary file 1 [file cancers-14-05976-s001.zip › Original images for blots or gels/Fig5-D/p-PI3K(607)-3.tif]

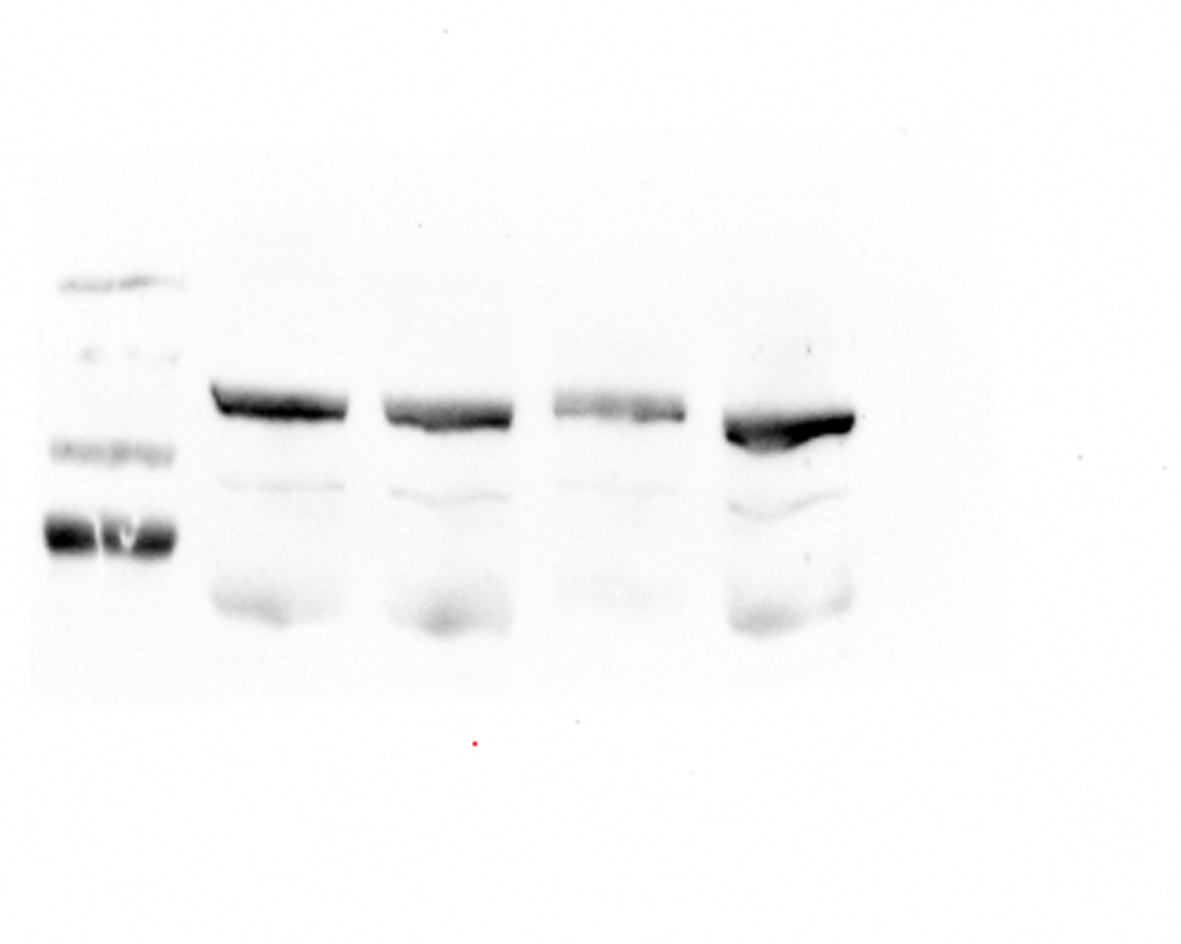

Supplement: Supplementary file 1 [file cancers-14-05976-s001.zip › Original images for blots or gels/Fig5-D/p-PI3K(607)-4.tif]
